# Do community pharmacies unnecessarily recommend oral emergency contraception? A nationwide mystery caller investigation in Germany

**DOI:** 10.1371/journal.pone.0361027

**Published:** 2026-10-23

**Authors:** Bernhard Langer, Anne Beck, Antonia M. Dreier, Rosa Gabrieljan, Hanna Hilgenhof, Johanna Latuske, Carolin Priepke, Katharina Schaihadinow, Helene Schwarz, Christian Kunow

**Affiliations:** Department of Health, Nursing, Management, Neubrandenburg University of Applied Sciences, Neubrandenburg, Germany; Connexi, INDIA

## Abstract

**Background:**

Since 2015, oral emergency contraceptives (ECs) have been available in German community pharmacies without prescription as over-the-counter medicines. Given the financial burden, unnecessary hormonal exposure and associated side effects, systematic information gathering is crucial to prevent unnecessary recommendations. This study aimed to determine whether pharmacy staff ask the checklist questions recommended by the German Federal Chamber of Pharmacists (BAK) guideline and provide correct recommendations when oral EC is not indicated (appropriate outcome), and to examine associated factors.

**Methods:**

A nationwide study using the simulated patient methodology – considered the “gold standard” – was conducted with a stratified random sample of 376 community pharmacies across Germany. Eight trained female mystery callers aged 21–24 simulated a scenario in which a combined oral contraceptive was forgotten once after sexual intercourse. However, the pill could still be taken within the 12-hour window, making oral EC unnecessary according to the BAK guideline. To ensure quality, each call was monitored by a second observer.

**Results:**

Of 376 mystery calls conducted, 322 were included in the primary analysis. An appropriate outcome was achieved in 44.4% (143/322), while oral EC was unnecessarily recommended in 49.1% (158/322). The median questioning score was 2.0 of 4.0 points. A multivariable logistic regression model showed that a higher questioning score and mystery caller identity were significantly associated with the appropriate outcome. Qualitative findings indicated both positive counseling practices and notable deficiencies among pharmacy staff.

**Conclusion:**

The results show deficits in information gathering and appropriate recommendations. To improve counseling, the consistent use of the BAK checklist during telephone consultations should be explicitly recommended in the relevant guideline. Targeted training for pharmacy staff may help address professional and communication deficits. Surprisingly, some mystery callers were significantly associated with the appropriate outcome, highlighting the need for further research on the influence of vocal characteristics and last names in telephone consultations.

## Introduction

Globally, an estimated 48.0% of all pregnancies are unintended [1]. In Germany, the corresponding proportion is lower, at 37.0% [2]. Although this proportion is approximately eleven percentage points below the international average, unintended pregnancies remain a substantial burden for affected women, with the potential for serious physical, emotional, and social consequences [3–5]. To prevent pregnancy after unprotected sexual intercourse (UPSI), the World Health Organization recommends the use of emergency contraception (EC) [6].

In Germany, the most widely known form of EC is oral EC [7]. Two oral EC preparations are available, containing either the active ingredients levonorgestrel (LNG) or ulipristal acetate (UPA) [8]. LNG is effective for up to 72 hours after UPSI, whereas UPA has a longer window of effectiveness of up to 120 hours and is considered more effective overall [9,10]. Irrespective of the preparation, effectiveness increases the sooner oral EC is taken after UPSI [6].

To ensure rapid access, oral ECs have been available in Germany without prescription as over-the-counter (OTC) medicines since 2015 [11], although dispensing is restricted to community pharmacies (CPs) [12]. In 2024, 96.6% of the 895,000 packages sold were dispensed OTC [13], and sales volume has increased by 85.2% since 2015 [14]. This increase reflects improved access following the switch to OTC status, accompanied by a marked shift from prescription-based dispensing to self-medication [14]. Although pharmacy and OTC access may facilitate more timely use of oral EC, available evidence has not demonstrated a clear reduction in unintended pregnancy compared with prescription-only access [15]. This development underscores the increasingly important role of CPs in access to and counseling on oral EC. In Germany, counseling may be provided not only by pharmacists but also by non-pharmacists, such as pharmaceutical engineers and pharmaceutical technical assistants, if authorized by the pharmacy manager [16].

German pharmacists are legally required to provide “adequate” counseling [16]. To support this, the German Federal Chamber of Pharmacists (BAK) published a guideline, most recently updated in 2021 [17]. This guideline recommends “personal” counseling and includes a checklist intended to ensure that relevant information is obtained prior to a recommendation. However, the term “personal” counseling remains insufficiently defined, particularly with regard to whether it includes telephone consultations. This lack of clarity is especially relevant given the increasing use of telephone-based counseling in CP practice [18]. The checklist questions are designed to collect the relevant information needed to determine whether oral EC is indicated and were therefore used to assess information gathering during counseling, but not to determine the appropriateness of the recommendation itself. However, the use of the checklist is neither mandatory nor explicitly recommended in the guideline. Importantly, the guideline also provides clear recommendations for cases of missed intake of a combined oral contraceptive: if the pill is taken less than 12 hours late, oral EC is not required [17].

An unnecessary recommendation and subsequent purchase of oral EC as an OTC medicine may impose a financial burden on the women concerned [19,20]. This is because the cost of oral EC is relatively high [21,22] compared to other OTC medicines, especially for minor ailments [23–26]. Furthermore, in Germany, statutory health insurance only reimburses the cost (minus a copayment) for women up to their 22nd birthday and only if a prescription is issued [27]. Possible financial consequences may be accompanied by unnecessary hormonal exposure and associated side effects for affected women. Common side effects occurred at similar frequencies with UPA and LNG. In a randomized trial, headache was reported by 19.3% and 18.9%, dysmenorrhoea by 12.9% and 14.3%, nausea by 12.8% and 11.3%, and abdominal pain by 5.1% and 6.7% of women receiving UPA and LNG, respectively [28]. Furthermore, the use of oral EC can be associated with shame, stigma, and fear of judgment for the women affected [29–31]. These financial, physical, and psychosocial implications underscore the importance of “adequate” counseling.

Nevertheless, consistent with findings from international studies [32–40], counseling deficits regarding oral ECs have also been identified in German CPs [41–43]. Importantly, the latter studies did not examine questions relating to correct recommendations. In another German study, however, pharmacists surveyed stated that in cases of questionable need (e.g., when combined oral contraceptives were taken less than 12 hours late), they still dispensed oral ECs in almost half of all cases [44]. Given that this was a self-reported survey and therefore susceptible to social desirability bias [45], the true prevalence is likely underestimated [46,47].

The simulated patient methodology (SPM), implemented either through covert on-site visits or via covert mystery callers (MCs), helps to avoid these disadvantages [45]. Two SPM visit studies conducted in Australia showed that pharmacists frequently provided unnecessary recommendations and dispensed oral EC [48,49]. To date, the only SPM visit study on oral EC in Germany examined the converse scenario, in which UPA dispensing was indicated, and reported incorrect recommendations [50]. The first MC study on oral EC conducted in Europe – and therefore also in Germany – identified certain counseling problems [21], but did not address unnecessary recommendations. These documented counseling deficits, along with possible commercial incentives [51], suggest that oral ECs may be recommended (and sold) unnecessarily. To our knowledge, no prior MC study worldwide has explicitly quantified the extent of unnecessary oral EC recommendations.

Using an MC approach, this study therefore aimed to investigate whether pharmacy staff ask the questions included in the BAK checklist and make appropriate recommendations in a scenario in which oral EC is not indicated (appropriate outcome, AO). Secondarily, we aimed to assess the influence of independent variables on the AO.

## Materials and methods

### Study design

This cross-sectional study was based on the internationally established SPM in the CP setting [52,53]. Mystery calls were used as the specific form of SPM [18,54,55]. In this context, MCs contacted a CP by telephone as covert participant observers. Based on a predefined scenario, the MCs simulated a realistic conversation. Each call was subsequently evaluated using a standardized assessment form containing predefined items, enabling the identification of potential quality deficits [56].

Reporting of this study followed the STROBE statement for cross-sectional studies [57]. In addition, the simulation research guidelines in health care [58], and the “Checklist for Reporting Research using Simulated Patient methodology in Health” (CRiSPHe) [59] were followed.

### Mystery callers

Eight female master’s students (ABe, AMDr, RGa, HHi, JLa, CPr, KSc, and HSc) from the Department of Health, Nursing, Management at Neubrandenburg University of Applied Sciences served as MCs as part of a three-semester research project free of charge. All MCs spoke standard German and had no prior experience with mystery calls. The MCs were between 21 and 24 years old at the time of data collection.

### Setting and participation

The mystery calls were conducted nationwide in Germany between July 15, 2024, and August 22, 2024. Due to the covert study design, the state chambers of pharmacists were not contacted to provide a comprehensive list of all CPs. Instead, the publicly accessible software of the Federal Pharmacy Register, version 3.6.0.9 (data status: February 2024), was used to determine the target population [60]. This register contains all German CPs, including name, address (with federal state), and telephone number. A total of 17,583 CPs listed in the register were proportionally stratified by federal state between March 1, 2024, and March 20, 2024.

To date, no MC studies in Germany or internationally have examined unnecessary recommendations of oral ECs by CPs. Consequently, the proportion of the characteristic of interest in the target population was unknown. Setting this parameter to P = 0.5 maximizes the required sample size.

Using the online sample size calculator “Raosoft” [61], the following parameters were applied:

- A population size of N = 17,583,
- A conventional margin of error (e) of 0.05,
- A conventional 95% confidence level, and
- A proportion of the characteristic of interest of P = 0.5.

This calculation resulted in a minimum required sample size (n) of 376 CPs.

The stratified list of 17,583 CPs was assigned random numbers using the Microsoft Excel random number generator (Microsoft® Excel version 2404, Microsoft Corporation, Redmond, USA). Within each stratum, a simple random sample proportional to the stratum’s share of all CPs was drawn, yielding the required 376 CPs (stratified random sampling).

The preliminary sample was further verified as of March 27, 2024, using a Google search performed by the MCs. Only CPs confirmed to be operational were included. This verification ensured the exclusion of CPs that had closed or could not be identified.

For each excluded CP, replacements were drawn for the respective federal state using the Excel random number generator. If, despite verification, CPs were unexpectedly closed or unreachable during the execution of the mystery calls, they were replaced ad hoc by additional CPs drawn from the corresponding stratum.

### Scenario and assessment

In an initial step, the MCs familiarized themselves with the theoretical foundations of the SPM. They were supported in this process by BLa, who already had extensive experience with SPM. The two instruments, scenario and assessment form (Tables 1 and 2), were jointly developed by the MCs and BLa based on the BAK guideline [17]. In particular, the included checklist was used [17]. This ensured that the participating pharmacy staff would be able to respond appropriately to the described situation. Furthermore, the planned scenario and assessment form underwent content validation by CKu, who also had extensive SPM experience. Two pharmacists who had not been involved in the development of the two instruments also participated in the validation. This validation did not result in any need to adapt the instruments.

**Table 1:** Scenario.

The MC began the conversation with: “Hello, this is Ms. [last name of MC], I’m not sure if I need oral EC. Could you please advise me?” In response to possible follow-up questions from the pharmacy staff, the MC provided the following information:	
Possible follow-up questions from pharmacy staff (based on the BAK checklist [17])	Standard answers for the MC
1. “How old are you?“	“I’m 24.“
2. “Why do you need oral EC?”	“I had sexual intercourse and forgot to take the pill.“
3. “What is the brand name of your pill?”	“I take the pill *Maxim*.” (pill with a 12-hour intake window)
4. “Which tablet number did you forget to take?”	“Let me check quickly…it was the fourth tablet.” (21 pills)
5. “How many pills did you forget to take?“	“Just the one from this morning.” (if asked: at 7 a.m.)
6. “When did you last take the pill?”	“Yesterday morning“ (if asked: at 7 a.m.)
7. “When did the sexual intercourse occur?“	“Last night.“
8. “When was your last menstrual period?“	“9 days ago.” (first day of menstruation)
9. “Was the first day of your last period more than 28 days ago?“ 10. “Was the last period lighter than usual?” 11. “Was the last period shorter than usual?“ 12. “Was the last period otherwise unusual?“	Answers to 9–12: “No.”
13. “Do you have any acute health problems or chronic diseases?“	“No.“
14. “Are you currently breastfeeding?“	“No.“
15. “Are you currently taking any medication?“	“No.“
16. “Have you ever used oral EC before?”	“No.“
If the pharmacy staff suggest an in-person visit to the CP, the MC responds according to the following guideline: 1. “I can’t right now, could you please advise me over the telephone?“ 2. If asked why: “I have to look after my little nephew today.“ 3. If asked to bring the nephew along: “He is sick.“ No further information is provided, not even if the mystery call is prematurely terminated by the pharmacy staff (exit).	

**Table 2:** Assessment items.

Items		
	Yes	No
(1) Did the staff ask about the woman’s age?	1	0
(2) Did the staff ask about the reason for requesting oral EC?	1	0
(3) Did the staff ask about the brand name of the pill?	1	0
(4) Did the staff ask about the sequence number of the missed pill(s)?	1	0
(5) Did the staff ask about the total number of missed pills?	1	0
(6) Did the staff ask when the last pill was taken?	1	0
(7) Did the staff ask about the timing of UPSI?	1	0
(8) Did the staff ask about the date of the last menstrual period?	1	0
Did the staff ask whether		
(9) the last menstrual period was more than 28 days ago?	1	0
(10) the last menstrual period was lighter than usual?	1	0
(11) the last menstrual period was shorter than usual?	1	0
(12) the last menstrual period was otherwise unusual?	1	0
(13) Did the staff ask whether acute health problems or chronic conditions are present?	1	0
(14) Did the staff ask whether the woman is currently breastfeeding?	1	0
(15) Did the staff ask whether other medications are being taken?	1	0
(16) Did the staff ask whether oral EC had been used previously?	1	0
(17) Did the staff recommend oral EC?	1	0
(18) Did the staff recommend that oral EC was not needed?	1	0
(19) Did the staff recommend consulting a physician?	1	0
(20) Did the staff recommend an in-person visit to the CP?	1	0
(21) Did the staff recommend the intake of the combined oral contraceptive by		
• immediately,	1	0
• taking the missed pill,	1	0
• and then continuing as scheduled?	1	0
(22) Were there any specific occurrences not captured by the assessment items?	................................	

The scenario (Table 1) was designed so that the MC requested oral EC for herself. To initiate the telephone conversation, the MC stated that she was uncertain whether she needed oral EC. The pharmacy staff were then explicitly asked to provide counseling and were expected, in an ideal consultation, to elicit additional information to support the decision-making process. Based on the typology proposed by Collins et al., this represents a product-based scenario [45]. It was further decided that all MCs would report a uniform age of 24 years during the conversations. This age was considered plausible, given that the primary users of oral ECs are women between 21 and 35 years of age [62].

The scenario depicts a missed intake of a combined oral contraceptive following sexual intercourse. The pill “Maxim” [63] was selected as it is reported to be the most frequently dispensed preparation for combined oral contraceptives in Germany [64]. Requesting oral EC due to a missed intake of the combined oral contraceptive also represents a realistic scenario. For instance, 31.0% of 14- to 25-year-old women surveyed by Scharmanski and Hessling [65] reported having used oral EC at least once because they had forgotten to take their regular pill. In a comparable survey of pharmacists, Said et al. [44] even found a proportion of 66.4%. In the scenario described here, sexual intercourse occurs prior to the missed intake of the combined oral contraceptive. Since sperm can remain active in the female body for up to five days [66,67], pregnancy in this situation would theoretically be possible. However, because in the constructed case only a single pill was missed and less than 12 hours had elapsed, the use of oral EC would not be necessary (AO) [17,68]. At the time of the mystery call, the missed pill had not yet been taken.

In developing the scenario, care was taken to ensure that, apart from the fact that the 12-hour window for delayed intake of the combined oral contraceptive had not yet elapsed, the conditions for recommending oral EC according to the BAK guideline would otherwise have been met. For this reason, the scenario was constructed so that the MC was in the first week of the pill cycle and sexual intercourse had taken place within the previous five days [17].

If the consulted pharmacy staff asked the MCs during the call to visit the pharmacy in person, this was uniformly declined, if necessary, using a three-step sequence of justifications. This procedure was intended to minimize the risk of the telephone consultation being discontinued.

The quantitative assessment form (Table 2) consists of 21 items. The first 16 items capture whether pharmacy staff ask the questions recommended in the BAK checklist [17]. Items 17–21 capture the counseling outcome. The AO in this scenario is the recommendation that oral EC is not required (item 18). Item 21 assesses whether the pharmacy staff provide the correct recommendations regarding the continued intake of the combined oral contraceptive, in accordance with the BAK guideline [17].

Beyond the collection of quantitative data, qualitative data on specific occurrences were also recorded based on free-text responses during the mystery calls (item 22) [69].

In addition to the items included in the assessment form, the MCs collected numerous variables before, during, and after the mystery calls (Table 3), which, according to the international literature, might influence the AO. Since only items 2, 3, 5, and 6 needed to be addressed in order to identify the AO, a “questioning score” consisting solely of these items was created. This score, also in line with the international literature, was considered a potential influencing factor on the AO (Table 3). A differentiated weighting of the individual items was omitted, as this would have depended on subjective considerations.

**Table 3:** Potential influencing factors, time and type of data collection.

Potential influencing factors [Literature source *]	Time of data collection	Type of data collection
MC identity [70]	Before the mystery calls	Exact measurement by randomly assigning CPs to the respective MCs
Mystery call attempts [71]	During the mystery calls	Exact measurement based on the number of call attempts
Time of the mystery calls [72]	During the mystery calls	Exact measurement using the MCs’ watches
Forwarding to other staff [73]	During the mystery calls	Exact measurement by acoustic impression of the MCs
Placed on hold after call acceptance [74]	During the mystery calls	Exact measurement of waiting time using the MCs’ watches
Background noise in the CPs (e.g., other pharmacy staff conversations) [75]	During the mystery calls	Exact measurement by acoustic impression of the MCs
Gender of the pharmacy staff [76]	During the mystery calls	Estimated measurement by acoustic impression of the MCs
Location of the CPs [77] – urban/rural area (2022) [78]	After the mystery calls	Exact measurement by assigning the spatial structure to the corresponding CPs
Purchasing power by cities/districts of the CPs [79] – average per inhabitant in € (2024) [80]	After the mystery calls	Exact measurement by assigning the purchasing power data of the cities/districts to the corresponding CPs
Call duration [81]	During the mystery calls	Exact measurement based on the display of the MCs’ telephones
Questioning score [82]	After the mystery calls	Exact measurement by summing the dichotomous ratings of essential question items 2, 3, 5, and 6 (range: 0–4 points)

* The potential influencing factors were taken from the respective literature sources.

Purchasing power was defined as the disposable income of the population of a region, comprising net income after taxes and social security contributions, including received transfer payments [80]. It was categorized into three groups (≤ €25,500, €25,501–€29,500, and ≥ €29,501). This variable was included based on the hypothesis that higher local purchasing power may be associated with greater incentives for CPs to recommend comparatively expensive oral EC unnecessarily because customers may be better able to afford it, whereas lower purchasing power may be associated with lower incentives.

### Data collection

The allocation of the selected CPs to the MCs was carried out randomly. Each of the eight MCs was therefore assigned 47 CPs (376 CPs / 8 MCs = 47 CPs). In addition to the main study, a pretest with a total of 40 validation calls was initially conducted. The distribution of five validation calls per MC was likewise determined randomly. Subsequently, a workshop was held to exchange experiences and to provide mutual feedback on the specific characteristics of the scenario and the assessment form. The pretests also demonstrated the need to adapt the scenario. Specifically, the introductory statement was modified to emphasize the MCs’ uncertainty about whether oral EC was required and to explicitly request professional counseling from the pharmacy staff. The revised scenario was then re-tested with two validation calls per MC.

Data collection took place on different weekdays and at various times of day within a time window from 12:00 p.m. to 6:00 p.m. This scheduling was based on the scenario in which the regular pill should have been taken at 7:00 a.m. A call placed before 12:00 p.m. could have occurred too close to the missed pill intake, making it realistic that affected women would have simply taken the missed pill instead of contacting a CP. All calls were initiated no later than 6:00 p.m. Thus, even for calls initiated at 6:00 p.m., no more than 11 hours had elapsed since the scheduled intake time of 7:00 a.m., ensuring that the MC was still within the 12-hour window. Calls were not initiated after 6:00 p.m. to avoid approaching the 12-hour threshold more closely.

Each CP was initially contacted once by its assigned MC. Mystery calls were placed from the MCs’ private mobile telephones with the caller ID withheld, in order to prevent any call-backs and to avoid alerting other CPs to the study. If a CP could not be reached, the MCs made up to three call attempts at five-minute intervals on the same day. This was intended to simulate a realistic emergency situation in which, after several unsuccessful attempts, the next CP would be contacted. CPs that could not be reached were marked as unavailable, and replaced by a randomly selected CP from the same stratum.

To ensure quality assurance, a second observer [83] monitored the MC’s call via the mobile telephone’s speaker. Four teams consisting of two MCs each were formed, with one person serving as the second observer for the other. The second observer was responsible for entering the data into the online data collection platform Jotform (Jotform, San Francisco, USA) in parallel with the MC’s conversation. Subsequently, the MC and the second observer cross-checked the digitally completed evaluation form using the four-eyes principle. Any disagreements were resolved between them. If no resolution was possible, the second observer’s assessment was considered decisive. To avoid potential bias in subsequent calls, the pharmacy staff were not informed at the end of the conversation that it was a mystery call. This ensured that advance warning of other CPs was not possible.

### Data management and analysis

Each two-person team conducted logical consistency checks (e.g., verifying that mutually exclusive items, such as Items 17 and 18, were not both selected) on the dataset of another randomly assigned team to further ensure data quality. No data implausibilities were detected during these checks, supporting the consistency of the dataset. Data analysis was performed using SPSS version 29 for Windows (IBM, Armonk, USA). Within descriptive statistics, frequencies and percentages were calculated for categorical data. Both the Kolmogorov-Smirnov and the Shapiro-Wilk tests indicated that the continuous data were not normally distributed. Therefore, the median, interquartile range (IQR), minimum, and maximum were calculated. In addition, 95% confidence intervals for both categorical and continuous data were reported using bootstrapping. As a sensitivity analysis, all prematurely terminated mystery calls (exits) were alternatively treated as inappropriate outcomes to assess the robustness of the estimated proportion of AO to this alternative classification.

A binomial logistic regression model was also developed to identify the influence of various independent variables (Table 3) on the dichotomous AO. The independent variables showed neither outliers nor multicollinearity. Following Hosmer et al. [84], variables with a p-value of less than 0.25 in the bivariate analysis were included in the multivariate analysis. Odds ratios, 95% confidence intervals, and p-values were reported. Nagelkerke’s R² was used to assess model fit. Effect size was measured using Cohen’s f², with values of 0.02, 0.15, and 0.35 indicating small, medium, and large effects, respectively [85]. A two-tailed p-value of less than 0.05 was considered statistically significant in all analyses.

The free-text statements recorded by the second observers were independently coded by three authors, who developed the codes, subcategories, and categories inductively from the material. The resulting codes, subcategories, and categories were compared and discussed, and disagreements were resolved by consensus. The coding framework was subsequently reviewed and validated by an additional author, resulting in minor refinements to the category system. To further illustrate the findings, corresponding anchor examples were also provided [86–88]. No qualitative data analysis software was used.

### Ethical approval

The study protocol was approved by the institutional ethics committee of the Neubrandenburg University of Applied Sciences (registration number: HSNB/220/24). In line with international literature [56], the mystery calls were conducted covertly and without the option for opt-out. This approach was intended to avoid bias from the Hawthorne effect [89] as well as selection bias [90]. However, to ensure transparency, all participating CPs were notified by letter in June 2024 about the conduct of the study, adherence to ethical research principles, and the provision of general, non-pharmacy-specific feedback after completion of the research project. This procedure followed recommendations in international literature [90,91] and was consistent with the implementation in numerous studies [92–94]. Specific information about the scenario used was not disclosed in order to protect the covert study design. For the same reason, the letter indicated only a broad time frame (“The study will be conducted during the course of 2024”) rather than a concrete date. Thus, the mystery calls were carried out at an unknown point in time from the CPs’ perspective. The collected data were pseudonymized and processed in accordance with the Guideline for the Use of Mystery Research in Market and Social Research [95], ensuring that individual CPs and their staff could not be identified. Access to the pseudonymized study data and the linkage list was restricted to the research team, and the data were stored securely. The linkage list will be permanently deleted after publication of the study findings and distribution of the scientific publication to the contacted CPs as general, non-pharmacy-specific performance feedback. The authors also signed a written commitment to treat all experiences and data with strict confidentiality both during and after the study [91]. The students who served as MCs likewise signed a declaration confirming their consent to act as MCs.

## Results

During data collection, 23 of the 376 CPs had to be replaced. The reasons for this were as follows: inability to reach the CP after three call attempts (10 CPs), permanent closure of the CP (7 CPs), unplanned deviation from the scenario (2 CPs), closure of the CP at the time of the call (2 CPs), and a callback request outside the specified time window (1 CP). In addition, one CP was duplicated in the sample (1 CP). In the end, as planned, 376 mystery calls were successfully conducted (target sample achieved: 100.0%). Of these, 54 calls (14.4%) were classified as exits, resulting in a final analytic sample of 322 calls (85.6%) (Fig 1).

**Fig 1:**
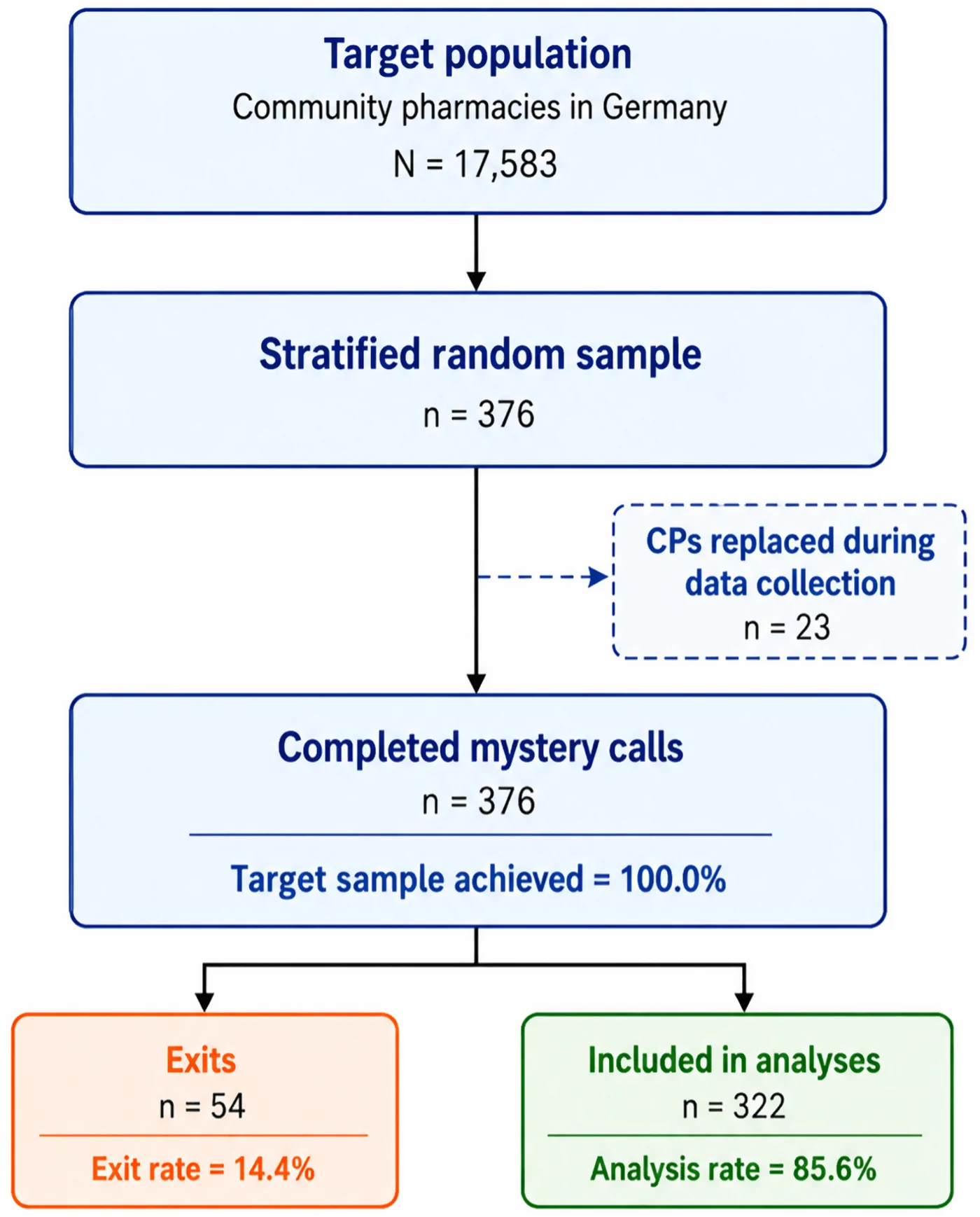
Flow of CPs and mystery calls through the study.

Characteristics of the MCs, mystery calls, pharmacy staff, and CPs are presented in Table 4.

**Table 4:** Characteristics of MCs, mystery calls, pharmacy staff, and CPs.

	Frequency (n)	Percentage (%)
	Total (376)	Total (100)
MC identity		
1	47	12.5
2	47	12.5
3	47	12.5
4	47	12.5
5	47	12.5
6	47	12.5
7	47	12.5
8	47	12.5
Mystery call attempts		
1st attempt	333	88.6
2nd attempt	37	9.8
3rd attempt	6	1.6
Time of the mystery calls		
12:00 p.m. - 3:00 p.m.	177	47.1
3:01 p.m. - 6:00 p.m.	199	52.9
Forwarding to other pharmacy staff		
No	248	66
Yes	128	34
Placed on hold after call acceptance		
No	280	74.5
≤ 1:00 min	68	18.1
> 1:00 min	28	7.4
Background noise in the CPs		
(e.g., other pharmacy staff conversations)		
No	359	95.5
Yes	17	4.5
Gender of the pharmacy staff		
Female	307	81.6
Male	69	18.4
Location of the CPs		
Rural	131	34.8
Urban	245	65.2
Purchasing power		
≤ €25,500	66	17.6
€25,501 - €29,500	220	58.5
≥ €29,501	90	23.9
Call duration		
≤ 1:30 min	76	20.2
1:31 - 3:00 min	144	38.3
> 3:00 min	156	41.5

Some interlocutors terminated the telephone conversation prematurely (exit) (14.4%, 54/376), which meant that not all items of the assessment form could be completed for all 376 mystery calls. Consequently, the sample sizes for items 1–21 differ. The AO was achieved in 44.4% of mystery calls (143/322). In the sensitivity analysis, the proportion of mystery calls achieving the AO decreased to 38.0% (143/376) when all 54 exits were treated as inappropriate outcomes. In 46.0% of mystery calls (148/322), the advice was given to take the missed combined oral contraceptive. In 37.0% of mystery calls (119/322), pharmacy staff additionally emphasized doing so immediately and continuing regular intake. In contrast, a recommendation to use oral EC was given in 49.1% of mystery calls (158/322) (Table 5).

**Table 5:** Assessment items.

	Total (n)	Frequency (n)^a^	Percentage (%)^a^	95% CI
(1) Did the staff ask for the woman’s age?	322	46	14.3	10.6–18.6
(2) Did the staff ask about the reason for requesting oral EC?	324	227	70.1	65.1–75.0
(3) Did the staff ask about the brand name of the pill?	322	140	43.5	37.9–48.8
(4) Did the staff ask about the sequence number of the missed pill(s)?	322	62	19.3	14.6–23.6
(5) Did the staff ask about the total number of missed pills?	323	142	44.0	39.0–49.8
(6) Did the staff ask when the last pill was taken?	323	155	48.0	42.4–53.3
(7) Did the staff ask about the timing of UPSI?	326	234	71.8	66.9–77.0
(8) Did the staff ask about the date of the last menstrual period?	325	170	52.3	47.1–57.5
Did the staff ask whether				
(9) the last menstrual period was more than 28 days ago?	322	2	0.6	0.0–1.6
(10) the last menstrual period was lighter than usual?	322	6	1.9	0.6–3.4
(11) the last menstrual period was shorter than usual?	322	5	1.6	0.3–3.1
(12) the last menstrual period was otherwise unusual?	322	15	4.7	2.5–6.8
(13) Did the staff ask whether acute health problems or chronic diseases are present?	323	36	11.1	7.7–14.9
(14) Did the staff ask whether the woman is currently breastfeeding?	322	16	5.0	2.8–7.5
(15) Did the staff ask whether any medications are being taken?	322	38	11.8	8.1–15.5
(16) Did the staff ask whether oral EC had been used previously?	323	20	6.2	3.7–9.0
(17) Did the staff recommend oral EC?	322	158	49.1	43.5–54.3
(18) Did the staff recommend that oral EC was not needed?	322	143	44.4	38.8–49.7
(19) Did the staff recommend consulting a physician?	324	29	9.0	5.9–12.0
(20) Did the staff recommend an in-person visit to the CP?	375	84	22.4	18.1–26.4
(21) Did the staff recommend the intake of the combined oral contraceptive by				
• immediately,	322	119	37.0	31.7–42.2
• taking the missed pill,	322	148	46.0	40.7–51.2
• and then continuing as scheduled?	322	119	37.0	31.7–42.2

^a^Results present the frequency and percentage of mystery calls with assessment “Yes”.

The median questioning score was 2.0 [IQR 1.0–3.0]. A minimum score of 0 was observed in 23.3% of mystery calls (75/322). Conversely, the maximum score of 4 was achieved in 24.5% of mystery calls (79/322), although the AO was not reached in 12.7% of these cases (10/79). Within the questioning score, the most frequently asked item concerned the reason for requesting oral EC (70.1%, 227/324). Moreover, the timing of UPSI – which was not essential for achieving the AO and therefore not part of the questioning score – was the most frequently asked question overall (71.8%, 234/326) (Table 5).

Table 6 presents the binary logistic regression model. In the bivariate analysis, six potential influencing factors (MC identity, mystery call attempts, forwarding to other pharmacy staff, placed on hold after call acceptance, call duration, and questioning score) showed a p-value of < 0.25. These variables were subsequently included in the multivariate logistic regression model. The analysis revealed that MC 2 (AOR = 9.88; 95% CI: 2.00–48.77), MC 3 (AOR = 6.31; 95% CI: 1.33–29.93), MC 4 (AOR = 24.19; 95% CI: 3.85–151.89), and MC 5 (AOR = 9.87; 95% CI: 2.15–45.40) were associated with a significantly higher likelihood of achieving the AO compared with MC 1. Furthermore, a significant positive association was observed between the questioning score (AOR = 5.32; 95% CI: 3.66–7.74) and the AO. All other variables showed no significant effects on the AO. The model reached a Nagelkerke R² value of 0.705, corresponding to a Cohen’s f² of 2.399, indicating a “large” effect size [85].

**Table 6:** Potential influencing factors on the AO.

Potential influencing factors	n (%) Total 322 (100)	n (%) Appropriate outcome 143 (44.4)	n (%) Inappropriate outcome 179 (55.6)	COR (95% CI)	*p-* Value	AOR (95% CI)	*p-* Value
MC identity							
1	41 (100)	9 (22.0)	32 (78.0)	1		1	
2	40 (100)	19 (47.5)	21 (52.5)	3.22 (1.23–8.45)	0.018	9.88 (2.00–48.77)	0.005*
3	44 (100)	17 (38.6)	27 (61.4)	2.24 (0.86–5.83)	0.099	6.31 (1.33–29.93)	0.020*
4	38 (100)	28 (73.7)	10 (26.3)	9.96 (3.54–27.99)	<0.001	24.19 (3.85–151.89)	<0.001*
5	40 (100)	24 (60.0)	16 (40.0)	5.33 (2.02–14.11)	<0.001	9.87 (2.15–45.40)	0.003*
6	40 (100)	19 (47.5)	21 (52.5)	3.22 (1.23–8.45)	0.018	1.90 (0.42–8.51)	0.403
7	40 (100)	13 (32.5)	27 (67.5)	1.71 (0.64–4.62)	0.288	1.51 (0.33–6.89)	0.598
8	39 (100)	14 (35.9)	25 (64.1)	1.99 (0.74–5.35)	0.172	2.51 (0.52–12.10)	0.252
Mystery call attempts							
1st attempt	281 (100)	121 (43.1)	160 (56.9)	1		1	
2nd attempt	35 (100)	19 (54.3)	16 (45.7)	1.57 (0.78–3.18)	0.210	2.03 (0.65–6.30)	0.221
3rd attempt	6 (100)	3 (50.0)	3 (50.0)	1.32 (0.26–6.67)	0.735	0.78 (0.02–30.48)	0.895
Time of the mystery calls							
12:00 p.m. - 3:00 p.m.	150 (100)	70 (46.7)	80 (53.3)	1			
3:01 p.m. - 6:00 p.m.	172 (100)	73 (42.4)	99 (57.6)	0.84 (0.54–1.31)	0.447		
Forwarding to other pharmacy staff							
No	200 (100)	81 (40.5)	119 (59.5)	1		1	
Yes	122 (100)	62 (50.8)	60 (49.2)	1.52 (0.97–2.39)	0.071	1.98 (0.84–4.70)	0.121
Placed on hold after call acceptance							
No	231 (100)	103 (44.6)	128 (55.4)	1		1	
≤ 1:00 min	65 (100)	23 (35.4)	42 (64.6)	0.68 (0.39–1.20)	0.186	1.18 (0.40–3.54)	0.763
> 1:00 min	26 (100)	17 (65.4)	9 (34.6)	2.35 (1.01–5.48)	0.049	1.15 (0.29–4.64)	0.845
Background noise in the CPs (e.g., other pharmacy staff conversations)							
No	306 (100)	134 (43.8)	172 (56.2)	1			
Yes	16 (100)	9 (56.2)	7 (43.8)	1.65 (0.60–4.55)	0.333		
Gender of the pharmacy staff							
Female	259 (100)	117 (45.2)	142 (54.8)	1			
Male	63 (100)	26 (41.3)	37 (58.7)	0.85 (0.49–1.49)	0.576		
Location of the CPs							
Rural	107 (100)	46 (43.0)	61 (57.0)	1			
Urban	215 (100)	97 (45.1)	118 (54.9)	1.09 (0.68–1.74)	0.718		
Purchasing power							
≤ €25,500	53 (100)	27 (50.9)	26 (49.1)	1			
€25,501 - €29,500	191 (100)	82 (42.9)	109 (57.1)	0.72 (0.39–1.33)	0.300		
≥ €29,501	78 (100)	34 (43.6)	44 (56.4)	0.74 (0.37–1.50)	0.408		
Call duration							
≤ 1:30 min	35 (100)	4 (11.4)	31 (88.6)	1		1	
1:31 - 3:00 min	131 (100)	42 (32.1)	89 (67.9)	3.66 (1.21–11.03)	0.021	1.39 (0.33–5.90)	0.655
> 3:00 min	156 (100)	97 (62.2)	59 (37.8)	12.74 (4.28–37.91)	<0.001	1.53 (0.34–6.89)	0.577
Questioning score ^a^	2.0 (1.0–3.0)	3.0 (3.0–4.0)	1.0 (0.0–2.0)	4.63 (3.43–6.26)	<0.001	5.32 (3.66–7.74)	<0.001*

^a^Median (IQR); Abbreviations: COR = Crude Odds Ratio, AOR = Adjusted Odds Ratio; *Significant at *p* < 0.05.

In the exploratory qualitative assessment of notable occurrences, a total of 99 statements made by pharmacy staff were recorded by the second observer. Through inductive categorization, four main categories and eleven subcategories were derived. Of the 11 subcategories identified, five reflected positive counseling aspects: “Recommendations against taking oral EC”, “Information on price and mode of action of oral EC”, “Additional contraceptive advice”, “Use of checklists and guidelines”, and “Follow-up questions / callback requests”. These subcategories comprised 35 of the 99 statements (35.4%). In contrast, six subcategories reflected problematic counseling aspects: “Recommendations for taking oral EC”, “No clear recommendation”, “Misinformation”, “Refusal to provide telephone counseling”, “Inappropriate remarks / morally questionable comments”, and “Unfriendliness on the part of the pharmacy staff”. These subcategories comprised 64 statements (64.6%). A detailed overview of all categories and subcategories, including anchor examples and absolute and relative frequencies, is provided in Table 7.

**Table 7:** Evaluation of the qualitative item with main categories, subcategories and anchor examples.

Main categories	Subcategories	(Mystery calls No.) anchor examples
Counseling outcomes and recommendations	Recommendations for taking oral EC (13; 13.1%)	(255) Paraphrased: “Better to take oral EC once too often than once too little.”
	Recommendations against taking oral EC (3; 3.0%)	(216) Based on the information that the first day of the last menstrual period was 9 days ago and sexual intercourse occurred the previous evening, it was stated that nothing could happen and that oral EC was not necessary.
	No clear recommendation (22; 22.2%)	(70) No clear recommendation was made, leaving the decision to the MC.
Knowledge transfer and education	Information on price and mode of action of oral EC (11; 11.1%)	(306) The MC was informed about different types of oral ECs and their prices.
	Additional contraceptive advice (6; 6.1%)	(016) Paraphrased: “After taking oral EC, also use condoms.”
	Misinformation (2; 2.0%)	(179) Paraphrased: “The 12‑hour window for taking the pill has already passed; it has already been 24 hours.”
Organizational aspects of counseling	Use of checklists and guidelines (12; 12.1%)	(226) Pharmacy staff went through the BAK checklist point by point.
	Refusal to provide telephone counseling (13; 13.1%)	(265) Paraphrased: “We do not do that over the telephone – go to a gynecologist or come in. I will definitely not do this over the telephone.”
	Follow‑up questions / callback requests (3; 3.0%)	(023) A call‑back was requested so that the pharmacy staff could take time to read the package leaflet.
Inappropriate communication	Inappropriate remarks / morally questionable comments (8; 8.1%)	(022) Paraphrased: “We sold oral EC nine times on Sunday while on duty. It’s our bestseller when we’re on duty!“
	Unfriendliness on the part of the pharmacy staff (6; 6.1%)	(244) Paraphrased: The pharmacy staff sigh when told that the MC has not yet read the package leaflet: “And you didn’t take the pill again?!” When the caller responds: “No”, the staff ask: “Why not?” and “Why didn’t you call this morning?”

## Discussion

### Appropriate outcome

The AO – in this case, not recommending oral EC – was achieved in just under half of all mystery calls. In contrast, a nationwide survey among pharmacists from CPs in Germany reported that, in a comparable scenario, oral EC was not dispensed in 56.3% of cases [44]. This higher value is not surprising [46,47] and is likely attributable to the survey design, which is prone to social desirability bias [45], as well as to voluntary CP participation (selection bias) [90].

An Australian SPM visit study using a scenario in which dispensing of oral EC was not indicated due to a missed inactive regular pill near the end of the menstrual cycle reported a substantially lower AO of 24.0% [48]. This is surprising, as the pharmacy staff in that study asked considerably more questions than in the present investigation. In another Australian SPM visit study by Smith et al. [49], using a scenario in which dispensing of oral EC was not indicated because the effective time window for oral EC had elapsed, the AO – i.e., refraining from dispensing – occurred in only 20.5% of cases. Differences in study design and counseling context may account for these discrepancies.

In this MC study, oral EC was explicitly recommended in 49.1% of cases, which is problematic in view of the potential financial [19,20], physical [28], and psychosocial consequences for affected women [29–31]. This is particularly concerning given that German citizens place substantial trust in counseling provided in CPs [96].

At the same time, CPs can play an important role in facilitating access to oral EC and other contraceptive services [71,94]. Given this important role, ensuring appropriate recommendations and high-quality counseling is essential to avoid unnecessary use. Such consultations may also provide an opportunity to reinforce adherence to regular contraception and, where appropriate, to discuss longer-term contraceptive options.

The specific reasons for the poor results cannot be determined using an MC approach. Therefore, further research employing other methods, such as questionnaires and interviews, is required. However, as demonstrated in the literature, several factors contributing to inadequate counseling quality can be inferred. Internationally, these include insufficient knowledge, lack of time and information among pharmacy staff, as well as limited human resources [46,97–99]. In contrast, pharmacy staff in Germany reported in a comparatively older study that they were concerned that customers might feel patronized by counseling [100]. A more recent study additionally identified concerns among pharmacists regarding the effective and safe use of oral EC, e.g., in cases of questionable need for oral EC, which were frequently associated with referrals to a gynecologist [44]. Such concerns may arise not only from insufficient knowledge or training, but also from a perception among pharmacists that oral EC counseling falls outside their professional scope of practice. This may also help explain why some pharmacy staff in the present study recommended consulting a physician. Another possible explanation is a precautionary approach by pharmacy staff. In a large German survey of 18,521 women, nearly half of oral contraceptive users reported having forgotten the pill at least once during the previous three months [101]. Pharmacy staff may therefore be particularly aware of adherence problems in everyday contraceptive use and may recommend oral EC as a precaution when a missed pill is reported, even when it is not indicated in the specific situation. Moreover, pharmacy staff have reported concerns that customers might feel uncomfortable when asked numerous questions during counseling [102]. Limited customer interest and time, as well as insufficient information about customers, have further been cited as reasons in the national literature [102–104]. However, this contrasts with survey findings showing that counseling is often desired, even when customers do not actively request it [105].

### Interventions for improvement

Considering that the AO was achieved in only 44.4% of mystery calls, as well as the uncertainties, unclear recommendations, and incorrect statements identified in the qualitative item of pharmacy staff, targeted training may represent a useful measure to enhance relevant professional competencies [104,106,107].

A heightened awareness in dealing with affected women should also be fostered in training programs in order to counteract inappropriate remarks by pharmacy staff, which were likewise identified in the qualitative item. A central focus should be on destigmatizing oral EC and preventing statements, behaviors, or value-laden attitudes that may lead to shame, stigma, or perceived judgment among the women concerned [29–31]. In this context, appropriate communication should already be practiced during vocational training for pharmaceutical technical assistants and pharmacy degree programs [104,108,109]. Given that continuing professional education is part of pharmacists’ professional obligations in Germany [110], oral EC counseling could also be incorporated into ongoing professional education programs.

Another possible intervention involves the use of a checklist [36,43,50,107,109], which should be readily available during telephone consultations. This is further supported by the significant positive association between the questioning score and the AO. This association underscores the importance of asking questions. Nevertheless, the findings also demonstrate that even asking essential questions does not necessarily prevent incorrect conclusions and thus the failure to achieve the AO. Moreover, empirical evidence from a German SPM visit study indicates that the BAK checklist is used in practice in only about half of counseling interactions [50], and some pharmacy staff perceive it as too extensive, overly complex, or insufficiently practice-oriented [41]. Accordingly, in addition to an explicit recommendation for the checklist’s use within the BAK guideline [50], an adaptation of this guideline also appears warranted to promote the continuous optimization of counseling practices [43,104].

In this context, for example, the term “personal” counseling should be specified more clearly to indicate that such counseling can also take place over the telephone. The need for this clarification is supported by the overall study results, as both the quantitative and qualitative findings show that telephone counseling frequently led to a premature end of the consultation (exit). This ambiguity also affects the interpretation of the AO. If telephone counseling is considered to fall within the BAK guideline’s concept of “personal” counseling, these exits may be regarded as a failure to achieve the AO. Accordingly, the proportion of mystery calls achieving the AO decreased from 44.4% to 38.0% in the sensitivity analysis. Conversely, if “personal” counseling is interpreted as requiring an in-person consultation, the AO cannot be assessed for these prematurely terminated calls. In addition to the aspects already discussed, the issue of access to CPs must also be addressed. Several CPs could not be reached even after three call attempts. This represents a barrier to patient care, particularly when medications are urgently needed, such as oral EC. The state chambers of pharmacists should therefore encourage CPs to ensure unrestricted telephone accessibility in light of their important role in patient care. Moreover, future research could explore the potential of digital decision-support tools, including AI-assisted counseling applications, to support oral EC counseling.

### Influencing factors

The regression analysis revealed a significant association between the AO and some of the MCs. Nationally and internationally, there are no studies with a comparable scenario that have identified such an association, which prevents direct comparison of the results [111]. Because the data were collected through mystery calls and all eight MCs followed a standardized scenario, it is reasonable to assume that the pharmacy staff could perceive only the MCs’ voices and last names as distinguishing features.

This raises the question of how the voices of four MCs differ compared with a reference MC. Possible differences between voices may include audibility, clarity, pace, or warmth [112]. The voice is therefore a highly individual human characteristic and can vary considerably from person to person [113]. Furthermore, in communication, vocal quality and speaking style are often attributed to greater importance than the actual content of what is said [114]. In general, higher-pitched voices are perceived more quickly than lower-pitched ones. However, lower-pitched voices tend to be perceived as more trustworthy and competent, whereas higher-pitched voices are often associated with childlike or emotional qualities [115,116]. To identify specific vocal factors that may facilitate a counseling conversation, further research using additional methods – such as questionnaires or interviews – would be required. In addition to the voice, it is also conceivable that the MCs’ last names contributed to the significant association observed between some MCs and the AO. However, the literature has not yet examined any relationship between last names and the quality of counseling. For this reason, further research is warranted in this area.

Consistent with other international SPM visit studies [50,82], the number of questions asked was significantly associated with the AO. This finding underscores the importance of asking questions to support an appropriate recommendation. Among the four essential questions (items 2, 3, 5, 6), the reason for the request (item 2) was asked most frequently – analogous to the Australian SPM visit study with a highly similar scenario, in which it was asked in 96.0% of cases [48]. In addition, the question about the timing of the UPSI (item 7) was the most frequently asked question overall. SPM visit studies conducted in Switzerland, Belgium, and Australia reported somewhat higher scenario-dependent rates – 100.0% [36], 96.1% [40], 95.0% [48], and 88.0% [34] – compared with this study (71.8%). This illustrates the basic reflex of pharmacy staff to ask this question [117]. Such a question is crucial in other scenarios that focus on the supply of oral EC. For example, the corresponding value in the German SPM visit study is 93.5% [50]. This shows that the question is often asked even when it is not relevant (as in our scenario). To ensure that the appropriate recommendation is given for each scenario, it is essential that pharmacy staff ask all questions specified in the BAK checklist.

### Strengths and limitations

The data were collected using the “gold standard” [45,118], the SPM, applied here in the form of mystery calls. This approach avoids both the Hawthorne effect and social desirability bias. Furthermore, for the first time worldwide – and thus also for the first time in Germany – an MC-based approach was used to examine whether oral EC is being recommended unnecessarily in CPs. Selection bias related to voluntary opt-out was avoided because CPs had no possibility to opt- out.

In addition, measures were taken to ensure that the MCs behaved as uniformly as possible during the mystery calls to avoid any distortion of the results. As is customary in SPM studies [45], this was achieved through standardized MC responses during the mystery calls, as well as through extensive MC training in the form of multiple pretests and a dedicated workshop. Another strength is the use of a second observer, which minimizes faulty recall during the call and thereby largely rules out recall bias [119,120]. However, formal inter-rater reliability coefficients were not calculated because ratings were cross-checked and reconciled rather than recorded independently. Furthermore, because the second observers were aware of the study aims and assessment criteria, observer expectancy bias cannot be completely excluded, although this knowledge facilitated accurate real-time assessment of the telephone conversations using the standardized assessment form.

Due to the cross-sectional design, temporal and causal relationships between the studied variables cannot be established. A further notable limitation is that pharmacy staff terminated the mystery call prematurely in 54 cases (exit). While the target sample of 376 mystery calls was achieved, exits led to reduced sample sizes for individual analyses. Because these premature terminations may not have occurred at random, selection bias in the final analytic sample cannot be completely excluded. Furthermore, because the initial sample size was not inflated to account for premature terminations, the minimum required sample size of 376 was not reached for the primary outcome analysis. This may have reduced the precision of the corresponding estimate. Future studies should consider prospectively inflating the sample size to account for anticipated exits or drawing additional CPs until the predefined number of evaluable mystery calls is achieved.

In addition, the preliminary verification of the operational status of sampled CPs relied on Google searches, which may have provided incomplete or outdated information and thus may have resulted in occasional misclassification. In this context, systematic differences between unreachable and successfully contacted CPs beyond the documented reasons for replacement cannot be excluded. Furthermore, the standardized three-attempt rule may not fully reflect real-world help-seeking behavior, as actual patients may discontinue their attempts or contact another CP after an unsuccessful call.

Other scenarios (e.g., a symptom-based scenario or one requiring a mandatory recommendation to consult a physician) could lead to different results, even in cases of identical indication [97,121]. Moreover, as mystery calls were restricted to the period between 12:00 p.m. and 6:00 p.m. for scenario-related reasons, the findings cannot be generalized to counseling provided outside this time window. Furthermore, due to the student research project setting, all MCs were young female university students. Therefore, it remains unclear whether callers of different ages, genders, educational backgrounds, or other sociodemographic characteristics would have received different counseling. This limits the generalizability of the findings to other caller populations.

Although numerous measures were taken to standardize MC behavior, complete standardization of all caller characteristics across multiple MCs is virtually impossible. However, additional standardization measures could have been considered. For example, the same surname could have been used by all MCs, and voice characteristics could theoretically have been further standardized using technical voice modification. In the present study, no objective measures of voice characteristics were collected. Therefore, the contribution of objectively measurable voice characteristics to the observed mystery caller effect could not be evaluated. In addition, although MC identity was included as a fixed effect in the regression analysis, residual within-MC correlation cannot be completely excluded. The pharmacy-specific performance feedback recommended in the international literature [56] was not provided in this MC study, as the very large sample size – compared with SPM visit studies – would have made this approach highly labor-intensive. Instead, the participating CPs will receive general, non-pharmacy-specific feedback in the form of a notification that the study results have been published in an international, peer-reviewed journal. Furthermore, it should be noted that counseling provided during mystery calls is likely not delivered to the same extent as during in-person visits [71]. One possible explanation is that pharmacy staff may assume that the MC would have to pick up oral EC at the CP anyway, making telephone counseling unnecessary [18]. In addition, mystery calls involved only one member of the pharmacy staff, implying that other staff members might have provided different counseling [122].

With regard to the additional collection of qualitative results, it should be noted that the content of some categories (“inappropriate remarks / morally questionable statements”, “unfriendliness on the part of the pharmacy staff”) could only be assessed subjectively. Moreover, the qualitative data were not collected systematically but only when the MC and the second observer judged an event to be noteworthy. Furthermore, no measures were taken to systematically assess whether pharmacy staff suspected that the calls were part of a mystery caller study. Such an assessment would have required cooperation with the CPs [123], which was incompatible with the study design (no opt-out). Although none of the pharmacy staff explicitly indicated such suspicion during the calls, undetected recognition cannot be excluded.

## Conclusion

The results indicate insufficient information gathering and a lack of appropriate recommendations. For this reason, the consistent use and explicit recommendation of the BAK checklist during telephone consultations should be considered in the relevant guideline. Appropriate training for pharmacy staff may help address professional and communicative deficits. Despite numerous standardization measures, some MCs showed a surprisingly significant association with the AO. There is a need for further research, particularly regarding the influence of the MCs’ voice and last name.
