# Supplementary material for: Do community pharmacies unnecessarily recommend oral emergency contraception? A nationwide mystery caller investigation in Germany
Source: PLoS One. 2026 Oct 23;21(10):e0361027. doi: 10.1371/journal.pone.0361027 (PMC13693899; doi:10.1371/journal.pone.0361027)
Supplement: Supplementary file 1 [file pone.0361027.s003.pdf]

PLOS One

Do community pharmacies unnecessarily recommend oral emergency contraception? A nationwide mystery caller investigation in Germany

--Manuscript Draft--

**Manuscript Number:**	PONE-D-26-20770
**Article Type:**	Research Article
**Full Title:**	Do community pharmacies unnecessarily recommend oral emergency contraception? A nationwide mystery caller investigation in Germany
**Short Title:**	Unnecessary recommendation of oral emergency contraception – a German mystery caller investigation
**Corresponding Author:**	Bernhard Langer<br>University of Applied Sciences Neubrandenburg: Hochschule Neubrandenburg<br>Neubrandenburg, GERMANY
**Keywords:**	Contraception; Community pharmacy services; Patient Simulation; self-medication; Mystery calls; Germany
**Abstract:**	Background: Since 2015, oral emergency contraceptives (ECs) have been available in German community pharmacies (CPs) without prescription as over-the-counter medicines. Given the financial burden, comparatively high hormone levels and possible side effects, systematic information gathering is crucial to prevent unnecessary recommendations of oral ECs. This study aimed to determine whether pharmacy staff ask the checklist questions recommended by the German Federal Chamber of Pharmacists (BAK) guideline and provide correct recommendations when oral EC is not indicated (appropriate outcome (AO)), and to examine factors associated with this outcome.<br>Methods: A nationwide study using the simulated patient methodology – considered the “gold standard” – was conducted with a representative, stratified random sample of 376 CPs across Germany. Eight trained female mystery callers (MCs) aged 21 to 24 simulated a scenario in which a combined oral contraceptive was forgotten once after sexual intercourse. However, the pill could still be taken within the 12-hour window, making oral EC unnecessary according to the BAK guideline. To ensure quality, each call was monitored by a second observer.<br>Results: 322 mystery calls were included in the final analysis. In 44.4% (143/322) of cases, the AO was achieved. The median questioning score was 2.0 out of 4.0 points. A multivariate logistic regression model showed that a higher questioning score and MC identity were significant factors of the AO. Qualitative findings indicated both positive counseling practices and notable deficiencies among pharmacy staff.<br>Conclusion: The results show deficits in information gathering and appropriate recommendations. To improve counseling, the consistent use of the BAK checklist during telephone consultations should be explicitly recommended in the relevant guideline. Targeted training for pharmacy staff could address professional and communication deficits. Surprisingly, some MCs significantly influenced the AO, highlighting the need for further research on the influence of vocal characteristics and last names in telephone consultations.
**Order of Authors:**	Bernhard Langer
	Anne Beck
	Antonia M. Dreier
	Rosa Gabrieljan
	Hanna Hilgenhof
	Johanna Latuske
	Carolin Priepke
	Katharina Schaihadinow
	Helene Schwarz

Powered by Editorial Manager® and ProduXion Manager® from Aries Systems Corporation

	Christian Kunow
**Additional Information:**	
**Question**	**Response**
**Financial Disclosure**<br><br>Enter a financial disclosure statement that describes the sources of funding for the work included in this submission. Review the submission guidelines for detailed requirements. View published research articles from *PLOS ONE* for specific examples.<br><br>This statement is required for submission and **will appear in the published article** if the submission is accepted. Please make sure it is accurate.<br><br>**Funded studies**<br>Enter a statement with the following details:<br>• Initials of the authors who received each award<br>• Grant numbers awarded to each author<br>• The full name of each funder<br>• URL of each funder website<br>• Did the sponsors or funders play any role in the study design, data collection and analysis, decision to publish, or preparation of the manuscript?<br><br>Did you receive funding for this work?	The author(s) received no specific funding for this work.
**Competing Interests**<br><br>Use the instructions below to enter a competing interest statement for this submission. On behalf of all authors, disclose any competing interests that could be perceived to bias this work—acknowledging all financial support and any other relevant financial or non-financial competing interests.<br><br>This statement is required for submission and **will appear in the published article** if the submission is accepted. Please make sure it is accurate and that any funding	The authors have declared that no competing interests exist.

sources listed in your Funding Information later in the submission form are also declared in your Financial Disclosure statement.<br><br>View published research articles from *PLOS ONE* for specific examples.<br><br>**NO authors have competing interests**<br><br>Enter: *The authors have declared that no competing interests exist.*<br><br>**Authors with competing interests**<br><br>Enter competing interest details beginning with this statement:<br><br>*I have read the journal's policy and the authors of this manuscript have the following competing interests: [insert competing interests here]*<br><br>* typeset	
**Ethics Statement**<br><br>Enter an ethics statement for this submission. This statement is required if the study involved:<br><br>• Human participants<br>• Human specimens or tissue<br>• Vertebrate animals or cephalopods<br>• Vertebrate embryos or tissues<br>• Field research<br><br>Write "N/A" if the submission does not require an ethics statement.<br><br>General guidance is provided below. Consult the submission guidelines for detailed instructions. **Make sure that all information entered here is included in the Methods section of the manuscript.**	The study protocol was approved by the institutional ethics committee of the Neubrandenburg University of Applied Sciences (registration number: HSNB/220/24). Due to the nature of the study, the mystery calls were conducted covertly and without the option for opt-out in order to minimize potential bias, including the Hawthorne effect and selection bias. To ensure transparency, all participating community pharmacies were notified by letter in June 2024 about the conduct of the study, adherence to ethical research principles, and the provision of general, non-pharmacy-specific feedback after completion of the research project.<br><br>Specific details about the study scenario were not disclosed in order to preserve the covert design. For the same reason, the notification letter indicated only a broad time frame ("The study will be conducted during the course of 2024") rather than a specific date, ensuring that the mystery calls occurred at an unknown point in time from the perspective of the pharmacies.<br><br>All collected data were pseudonymized and processed in accordance with established guidelines for mystery research in market and social research, ensuring that individual pharmacies and their staff could not be identified. The authors signed a written commitment to maintain strict confidentiality of all data and experiences during and after the study. Additionally, the students who served as mystery callers provided written declarations confirming their informed consent to participate in this role.

Powered by Editorial Manager® and ProduXion Manager® from Aries Systems Corporation

**Format for specific study types**<br><br>**Human Subject Research (involving human participants and/or tissue)**<br>• Give the name of the institutional review board or ethics committee that approved the study<br>• Include the approval number and/or a statement indicating approval of this research<br>• Indicate the form of consent obtained (written/oral) or the reason that consent was not obtained (e.g. the data were analyzed anonymously)<br><br>**Animal Research (involving vertebrate animals, embryos or tissues)**<br>• Provide the name of the Institutional Animal Care and Use Committee (IACUC) or other relevant ethics board that reviewed the study protocol, and indicate whether they approved this research or granted a formal waiver of ethical approval<br>• Include an approval number if one was obtained<br>• If the study involved *non-human primates*, add *additional details* about animal welfare and steps taken to ameliorate suffering<br>• If anesthesia, euthanasia, or any kind of animal sacrifice is part of the study, include briefly which substances and/or methods were applied<br><br>**Field Research**<br><br>Include the following details if this study involves the collection of plant, animal, or other materials from a natural setting:<br>• Field permit number<br>• Name of the institution or relevant body that granted permission	
**Data Availability**<br><br>Authors are required to make all data underlying the findings described fully available, without restriction, and from the time of publication. PLOS allows rare exceptions to address legal and ethical concerns. See the PLOS Data Policy and FAQ for detailed information.	Yes - all data are fully available without restriction

A Data Availability Statement describing where the data can be found is required at submission. Your answers to this question constitute the Data Availability Statement and **will be published in the article**, if accepted.<br><br>**Important:** Stating 'data available on request from the author' is not sufficient. If your data are only available upon request, select 'No' for the first question and explain your exceptional situation in the text box.<br><br>Do the authors confirm that all data underlying the findings described in their manuscript are fully available without restriction?	
**Describe where the data may be found in full sentences. If you are copying our sample text, replace any instances of XXX with the appropriate details.**<br><br>• If the data are **held or will be held in a public repository**, include URLs, accession numbers or DOIs. If this information will only be available after acceptance, indicate this by ticking the box below. For example: *All XXX files are available from the XXX database (accession number(s) XXX, XXX.).*<br>• If the data are all contained **within the manuscript and/or Supporting Information files**, enter the following: *All relevant data are within the manuscript and its Supporting Information files.*<br>• If neither of these applies but you are able to provide **details of access elsewhere**, with or without limitations, please do so. For example:<br><br>*Data cannot be shared publicly because of [XXX]. Data are available from the XXX Institutional Data Access / Ethics Committee (contact via XXX) for researchers who meet the criteria for access to confidential data.*<br><br>*The data underlying the results presented in the study are available from (include the name of the third party*	All relevant data are within the manuscript.

and contact information or URL).<br>• This text is appropriate if the data are owned by a third party and authors do not have permission to share the data.<br><br>* typeset	
Additional data availability information:	

Manuscript
Click here to access/download;Manuscript;2026-04-27 Manuscript MC EC Nationwide Germany.docx

Do community pharmacies unnecessarily recommend oral emergency contraception? A nationwide mystery caller investigation in Germany

Bernhard Langer, Professor of Health Care Management.[1,*]
https://orcid.org/0000-0001-8410-5229

Anne Beck, Research Associate.[1]
https://orcid.org/0009-0000-7616-9599

Antonia M. Dreier, Research Associate.[1]
https://orcid.org/0009-0008-4547-971X

Rosa Gabrieljan, Research Associate.[1]
https://orcid.org/0009-0008-4777-1175

Hanna Hilgenhof, Research Associate.[1]
https://orcid.org/0009-0004-9259-501X

Johanna Latuske, Research Associate.[1]
https://orcid.org/0009-0005-2400-7025

Carolin Priepke, Research Associate.[1]
https://orcid.org/0009-0004-5036-1741

Katharina Schaihadinow, Research Associate.[1]
https://orcid.org/0009-0009-2985-7378

Helene Schwarz, Research Associate.[1]
https://orcid.org/0009-0001-9024-4425

Christian Kunow, Research Associate.[1]
https://orcid.org/0000-0002-2674-0344

[1] Neubrandenburg University of Applied Sciences
Department of Health, Nursing, Management
Neubrandenburg
GERMANY

* Corresponding author:
Prof. Dr. Bernhard Langer
Neubrandenburg University of Applied Sciences
Department of Health, Nursing, Management
Brodaer Straße 2
17033 Neubrandenburg

GERMANY
Tel.: +49 (0)395/5693-3107
Email: langer@hs-nb.de

SHORT TITLE

Unnecessary recommendation of oral emergency contraception – a German mystery caller investigation

ABSTRACT

Background: Since 2015, oral emergency contraceptives (ECs) have been available in German community pharmacies (CPs) without prescription as over-the-counter medicines. Given the financial burden, comparatively high hormone levels and possible side effects, systematic information gathering is crucial to prevent unnecessary recommendations of oral ECs. This study aimed to determine whether pharmacy staff ask the checklist questions recommended by the German Federal Chamber of Pharmacists (BAK) guideline and provide correct recommendations when oral EC is not indicated (appropriate outcome (AO)), and to examine factors associated with this outcome.

Methods: A nationwide study using the simulated patient methodology – considered the “gold standard” – was conducted with a representative, stratified random sample of 376 CPs across Germany. Eight trained female mystery callers (MCs) aged 21 to 24 simulated a scenario in which a combined oral contraceptive was forgotten once after sexual intercourse. However, the pill could still be taken within the 12-hour window, making oral EC unnecessary according to the BAK guideline. To ensure quality, each call was monitored by a second observer.

Results: 322 mystery calls were included in the final analysis. In 44.4% (143/322) of cases, the AO was achieved. The median questioning score was 2.0 out of 4.0 points. A multivariate logistic regression model showed that a higher questioning score and MC identity were significant factors of the AO. Qualitative findings indicated both positive counseling practices and notable deficiencies among pharmacy staff.

Conclusion: The results show deficits in information gathering and appropriate recommendations. To improve counseling, the consistent use of the BAK checklist during telephone consultations should be explicitly recommended in the relevant guideline. Targeted training for pharmacy staff could address professional and communication deficits. Surprisingly, some MCs significantly influenced the AO,

highlighting the need for further research on the influence of vocal characteristics and last names in telephone consultations.

KEYWORDS

- Contraception
- Community pharmacy services
- Patient Simulation
- Self-medication
- Mystery calls
- Germany

Introduction

Globally, an estimated 48.0% of all pregnancies are unintended [**Bearak et al. 2020**]. In Germany, the corresponding proportion is lower, at 37.0% [**Guttmacher Institute 2025**]. Although this proportion is approximately eleven percentage points below the international average, unintended pregnancies continue to represent a substantial burden for affected women, with the potential for serious physical, emotional, and social consequences [**Yazdkhasti et al. 2015; Abajobir et al. 2017; Nelson et al. 2022**]. To prevent pregnancy after unprotected sexual intercourse (UPSI), the World Health Organization recommends the use of emergency contraception (EC) [**WHO 2021**].

In Germany, the most widely known form of EC is the so-called “morning-after pill” (oral EC) [**Renner 2015**]. Besides the combined oral contraceptive (Yuzpe method), which is not recommended in Germany, two preparations are available: Oral ECs containing either the active ingredients levonorgestrel (LNG) or ulipristal acetate (UPA) [**Rabe et al. 2013**]. LNG is effective for up to 72 hours after UPSI, whereas UPA has a longer window of effectiveness of up to 120 hours and is considered more effective overall [**Brache et al. 2013; Shen et al. 2019**]. Irrespective of the preparation, effectiveness increases the sooner oral EC is taken after UPSI [**WHO 2021**].

To ensure rapid access, oral ECs have been available in Germany without prescription as over-the-counter (OTC) medicines since 2015 [**AMVV 2024**], although dispensing is restricted to community pharmacies (CPs) [**ApoG 2023**]. In 2024, 96.6% of the 895,000 packages sold were dispensed OTC [**ABDA 2025**], and sales volume has increased by 85.2% since 2015 [**ABDA 2024**]. This development underscores the increasingly important role of CPs in counseling on oral EC. In Germany, counseling may be provided not only by pharmacists but also by non-pharmacists, such as pharmaceutical engineers and pharmaceutical technical assistants, if authorized by the pharmacy manager [**ApBetrO 2023**].

German pharmacists are legally required to provide “adequate” counseling [**ApBetrO 2023**]. To support this, the German Federal Chamber of Pharmacists (BAK) published a guideline, most recently updated in 2021 [**BAK 2021**]. This guideline recommends “personal” counseling and includes a checklist intended to ensure that relevant information is obtained prior to a recommendation. However, the term “personal” counseling remains insufficiently defined, particularly with regard to whether it includes

telephone consultations. This lack of clarity is especially relevant given the increasing role of telephone-based counseling in community pharmacy (CP) practice [**Kunow/Langer 2023**]. The checklist questions are designed to identify whether oral EC is indicated. However, the use of the checklist is neither mandatory nor explicitly recommended in the guideline. Importantly, the guideline also provides clear recommendations for cases of missed intake of a combined oral contraceptive: if the pill is taken less than 12 hours late, oral EC is not required [**BAK 2021**].

An unnecessary recommendation and subsequent purchase of oral EC as an OTC medicine may impose a financial burden on the women concerned [**Elmes et al. 2022; Torres et al. 2026**]. This is because the cost of oral EC is relatively high [**Lungfiel et al. 2023; Kunow et al. 2025**] compared to other OTC medicines, especially for minor ailments [**Langer et al. 2019; Kunow/Langer 2021; Langer et al. 2024; Langer et al. 2025**]. Furthermore, in Germany, statutory health insurance only reimburses the cost (minus a copayment) for women up to their 22nd birthday and only if a prescription is issued [**BMG 2021**]. Possible financial consequences may be accompanied by an unnecessary hormonal exposure and associated side effects for affected women. For LNG, these may include nausea, vomiting, abdominal pain, and dizziness. For UPA, delayed menstruation, abdominal pain, headache, and fatigue are reported [**Salcedo et al. 2023; Li et al. 2024**]. Furthermore, the use of oral EC can be associated with shame, stigma, and fear of judgment for the women affected [**Eastham et al. 2020; Chan et al. 2021; Szajbely et al. 2025**]. These financial, physical, and psychosocial implications underscore the importance of “adequate” counseling.

Nevertheless, consistent with findings from international studies [**Obare et al. 2013; Saxena et al. 2016; Collins et al. 2018; Hernandez et al. 2018; Haag et al. 2019; Uzun et al. 2019; Glasier et al. 2021; O’Neil/Aldanmaz 2022; Vander Steen 2024**], counseling deficits regarding oral ECs have also been identified in German CPs [**Bruhns 2015; Dierolf/Freytag 2017; Pro familia Bundesverband 2018**]. Importantly, the latter studies did not examine questions relating to correct recommendations. In another study, however, pharmacists surveyed stated that in cases of questionable need (e.g., when combined oral contraceptives were taken less than 12 hours late), they still dispensed oral ECs in almost half of all cases [**Said et al. 2019**]. Given that this was a self-reported survey and therefore susceptible to social

desirability bias [**Collins et al. 2021**], the true prevalence is likely underestimated [**Surur et al. 2017; Mengistu et al. 2019**].

The simulated patient methodology (SPM), implemented either through covert on-site visits or via covert mystery callers (MCs), helps to avoid these disadvantages [**Collins et al. 2021**]. Two SPM visit studies conducted in Australia showed that pharmacists frequently provided unnecessary recommendations and dispensed oral EC [**Schneider et al. 2013; Smith et al. 2019**]. To date, the only SPM visit study on oral EC in Germany has reported incorrect recommendations in cases where UPA dispensing would have been necessary [**Langer et al. 2020**]. The first MC study conducted in Europe - and therefore also in Germany - identified certain counseling problems [**Lungfiel et al. 2023**], but did not address unnecessary recommendations. These documented counseling deficits - along with possible commercial incentives [**Torres et al. 2020**] - suggest that oral ECs may be recommended (and sold) unnecessarily. To our knowledge, no prior MC study worldwide has explicitly quantified the extent of unnecessary oral EC recommendations.

Using an MC approach, this study therefore aimed to investigate whether pharmacy staff ask the questions included in the BAK checklist and make appropriate recommendations in a scenario in which oral EC is not indicated (appropriate outcome, AO). Secondarily, we aimed to assess the influence of independent variables on the AO.

Materials and methods

Study design

This cross-sectional study was based on the internationally established SPM in the CP setting [**Björnsdottir et al. 2020; Kunow/Langer 2025**], using mystery calls as the specific form of SPM [**Rankin et al. 2022; Kunow/Langer 2023; Madson et al. 2025**]. In this context, MCs contacted a CP by telephone as covert participant observers. Based on a predefined scenario, the MCs simulated a realistic conversation. Each call was subsequently evaluated using a standardized assessment form containing predefined items, enabling the identification of potential quality deficits [**Xu et al. 2012**].

Reporting of this study followed the STROBE Statement – Checklist of items that should be included in reports of cross-sectional studies [**STROBE Statement 2025**],

the simulation research guidelines in health care [**Cheng et al. 2016**], and the "Checklist for Reporting Research using Simulated Patient methodology in Health" (CRiSPHe) [**Park et al. 2024**].

Mystery callers

Eight female master's students (ABe, AMDr, RGa, HHi, JLa, CPr, KSc, and HSc) from the Department of Health, Nursing, Management at Neubrandenburg University of Applied Sciences served as MCs as part of a three-semester research project free of charge. All MCs spoke standard German and had no prior experience with mystery calls. The MCs were between 21 and 24 years old at the time of data collection.

Setting and participation

The mystery calls were conducted nationwide in Germany between July 15, 2024, and August 22, 2024. Due to the covert study design, the state chambers of pharmacists were not contacted to provide a comprehensive list of all CPs. Instead, the publicly accessible software of the Federal Pharmacy Register, version 3.6.0.9 (data status: February 2024), was used to determine the target population [**Deutscher Apotheker Verlag 2024**]. This register contains all German CPs, including name, address (with federal state), and telephone number. A total of 17,583 CPs listed in the register were proportionally stratified by federal state between March 1, 2024, and March 20, 2024.

To date, no MC studies in Germany or internationally have examined unnecessary recommendations of oral ECs by CPs. Consequently, the proportion of the characteristic of interest in the target population was unknown. Setting this parameter to P = 0.5 maximizes the required sample size.

Using the online sample size calculator "Raosoft" [**Raosoft 2004**], the following parameters were applied:

- a population size of N = 17,583,
- a conventional margin of error (e) of 0.05,
- a conventional 95% confidence level, and
- a proportion of the characteristic of interest of P = 0.5.

This calculation resulted in a minimum required sample size (n) of 376 CPs.

The stratified list of 17,583 CPs was assigned random numbers using the Microsoft Excel random number generator (Microsoft® Excel version 2404, Microsoft Corporation, Redmond, USA). Within each stratum, a simple random sample proportional to the stratum's share of all CPs was drawn, yielding the required 376 CPs (stratified random sampling).

The preliminary sample was further validated as of March 27, 2024, using a Google search performed by the MCs. Only CPs confirmed to be operational were included. This validation ensured the exclusion of CPs that had closed or could not be identified. In such cases, replacements were drawn for the respective federal state using the Excel random number generator. If, despite validation, CPs were unexpectedly closed or unreachable during the execution of the mystery calls, they were replaced ad hoc by additional CPs drawn from the corresponding stratum.

Scenario and Assessment

In an initial step, the MCs familiarized themselves with the theoretical foundations of the SPM. They were supported in this process by BLa, who already had extensive experience with SPM. The two instruments, scenario and assessment form (**Tables 1 and 2**), were jointly developed by the MCs and BLa based on the BAK guideline [**BAK 2021**]. In particular, the included checklist was used [**BAK 2021**]. This ensured that the participating pharmacy staff would be able to respond appropriately to the described situation. Furthermore, the planned scenario and assessment form underwent content validation by CKu, who also had extensive SPM experience, as well as by two pharmacists who had not been involved in the development of the two instruments. This validation did not result in any need to adapt the instruments.

The scenario (**Table 1**) was designed so that the MC requested the morning-after pill for herself. To initiate the telephone conversation, the MC stated that she was uncertain whether she needed the morning-after pill. The pharmacy staff were then explicitly asked to provide counseling and were expected, in an ideal consultation, to elicit additional information to support the decision-making process. Based on the typology proposed by Collins et al., this represents a product-based scenario [**Collins et al. 2021**]. It was further decided that all MCs would report a uniform age of 24 years during the conversations. This age was considered plausible, given that the primary users of oral ECs are women between 21 and 35 years of age [**Kiechle/Neuenfeldt 2017**].

The scenario depicts a missed intake of a combined oral contraceptive following sexual intercourse. The pill “Maxim” [**Jenapharm n. d.**], containing the active ingredients dienogest (2 mg) and ethinylestradiol (0.03 mg), was selected as it is reported to be the most frequently dispensed preparation for combined oral contraceptives in Germany [**Glaeske/Thürmann 2015**]. Requesting the morning-after pill due to a missed intake of the combined oral contraceptive also represents a realistic scenario. For instance, 31.0% of 14- to 25-year-old women surveyed by Scharmanski and Hessling [**2021**] reported having used the morning-after pill at least once because they had forgotten to take their regular pill. In a comparable survey of pharmacists, Said et al. [**2019**] even found a proportion of 66.4%. In the scenario described here, sexual intercourse occurs prior to the missed intake of the combined oral contraceptive. Since sperm can remain active in the female body for up to five days [**Suarez et al. 2006, van der Linde et al. 2015**], pregnancy in this situation would theoretically be possible. However, because in the constructed case only a single pill was missed and less than 12 hours had elapsed, the use of the morning-after pill would not be necessary (AO) [**Rabe et al. 2017; BAK 2021**].

In developing the scenario, care was taken to ensure that the use of oral EC would be indicated according to the BAK guideline, provided that the time window for the delayed intake of the combined oral contraceptive had already elapsed. For this reason, the scenario was constructed so that the MC was in the first week of the pill cycle and sexual intercourse had taken place within the previous five days [**BAK 2021**].

If the consulted pharmacy staff asked the MCs during the call to visit the pharmacy in person, this was uniformly declined, if necessary, using a three-step sequence of justifications. This procedure was intended to minimize the risk of the telephone consultation being discontinued.

The MC began the conversation with:

"Hello, this is Ms. [last name of MC], I’m not sure if I need the morning-after pill. Could you please advise me?“

In response to possible follow-up questions from the pharmacy staff, the MC provided the following information:

**Possible follow-up questions from pharmacy staff (based on the BAK checklist [BAK 2021])**	**Standard answers for the MC**
**1.** “How old are you?“	“I’m 24.“

**2.** "Why do you need a morning-after pill?"	"I had sexual intercourse and forgot to take the pill.“
**3.** "What is the brand name of your pill?"	"I take the pill *Maxim*." (pill with a 12-hour intake window)
**4.** "Which tablet number did you forget to take?"	"Let me check quickly…it was the fourth tablet." (21 pills)
**5.** "How many pills did you forget to take?“	"Just the one from this morning." (if asked: at 7 a.m.)
**6.** "When did you last take the pill?"	"Yesterday morning“ (if asked: at 7 a.m.)
**7.** "When did the sexual intercourse occur?“	"Last night.“
**8.** "When was your last menstrual period?“	"9 days ago." (first day of menstruation)
**9.** "Was the first day of your last period more than 28 days ago?“<br>**10.** "Was the last period lighter than usual?"<br>**11.** "Was the last period shorter than usual?“<br>**12.** "Was the last period otherwise unusual?“	Answers to 9–12: "No."
**13.** "Do you have any acute health problems or chronic diseases?“	"No.“
**14.** "Are you currently breastfeeding?“	"No.“
**15.** "Are you currently taking any medication?“	"No.“
**16.** "Have you ever used a morning-after pill before?"	"No.“
If the pharmacy staff suggest an in-person visit to the CP, the MC responds according to the following guideline:<br>1. "I can't right now, could you please advise me over the telephone?“<br>2. If asked why: "I have to look after my little nephew today.“<br>3. If asked to bring the nephew along: "He is sick.“<br>No further information is provided, not even if the mystery call is prematurely terminated by the pharmacy staff (exit).	

Table 1. *Scenario.*

The assessment form (**Table 2**) consists of 21 items. The first 16 items capture whether pharmacy staff ask the questions recommended in the BAK checklist [**BAK 2021**]. Items 17 to 21 capture the counseling outcome. The AO in this scenario is the recommendation that oral EC is not required. In addition, the final item assesses whether the pharmacy staff provide the correct recommendations regarding the continued intake of the combined oral contraceptive, in accordance with the BAK guideline [**BAK 2021**].

Beyond the collection of quantitative data, qualitative data on specific occurrences were also recorded based on free-text responses during the mystery calls (item 22) [**Tavares/Foster 2016**].

**Items**	Yes	No
(1) Did the staff ask about the woman's age?	1	0
(2) Did the staff ask about the reason for requesting oral EC?	1	0
(3) Did the staff ask about the brand name of the pill?	1	0
(4) Did the staff ask about the sequence number of the missed pill(s)?	1	0
(5) Did the staff ask about the total number of missed pills?	1	0
(6) Did the staff ask when the last pill was taken?	1	0
(7) Did the staff ask about the timing of UPSI?	1	0
(8) Did the staff ask about the date of the last menstrual period?	1	0
Did the staff ask whether		
(9) the last menstrual period was more than 28 days ago?	1	0
(10) the last menstrual period was lighter than usual?	1	0
(11) the last menstrual period was shorter than usual?	1	0
(12) the last menstrual period was otherwise unusual?	1	0
(13) Did the staff ask whether acute health problems or chronic conditions are present?	1	0
(14) Did the staff ask whether the woman is currently breastfeeding?	1	0
(15) Did the staff ask whether other medications are being taken?	1	0
(16) Did the staff ask whether oral EC had been used previously?	1	0
(17) Did the staff recommend oral EC?	1	0
(18) Did the staff recommend that oral EC was not needed?	1	0
(19) Did the staff recommend consulting a physician?	1	0
(20) Did the staff recommend an in-person visit to the CP?	1	0
(21) Did the staff recommend the intake of the combined oral contraceptive by		
• immediately,	1	0
• taking the missed pill,	1	0
• and then continuing as scheduled?	1	0
(22) Were there any specific occurrences not captured by the assessment items?	....	

***Table 2**. Assessment items.*

In addition to the items included in the assessment form, the MCs collected numerous variables before, during, and after the mystery calls (**Table 3**), which, according to the international literature, might influence the AO. Since only items 2, 3, 5, and 6 needed to be addressed in order to identify the AO, a "questioning score" consisting solely of these items was created. This score, also in line with the international literature, was considered a potential influencing factor on the AO (**Table 3**). A differentiated weighting of the individual items was omitted, as this would have depended on subjective considerations.

Potential influencing factors [Literature source *]	Time of data collection	Type of data collection
MC identity **[Zapata-Cachafeiro et al. 2019]**	Before the mystery calls	Exact measurement by randomly assigning CPs to the respective MCs
Mystery call attempts **[Stone et al. 2020]**	During the mystery calls	Exact measurement based on the number of call attempts
Time of the mystery calls **[Kashour et al. 2016]**	During the mystery calls	Exact measurement using the MCs‘ watches
Forwarding to other staff **[Wilkinson et al. 2018]**	During the mystery calls	Exact measurement by acoustic impression of the MCs
Placed on hold after call acceptance **[Uysal et al. 2019]**	During the mystery calls	Exact measurement of waiting time using the MCs‘ watches
Background noise in the CPs (e.g., other pharmacy staff conversations) **[Odukoya/Chui 2012]**	During the mystery calls	Exact measurement by acoustic impression of the MCs
Gender of the pharmacy staff **[Hussainy et al. 2015]**	During the mystery calls	Estimated measurement by acoustic impression of the MCs
Location of the CPs **[Alte et al. 2007]** – urban/rural area (2022) **[BBSR 2023]**	After the mystery calls	Exact measurement by assigning the spatial structure to the corresponding CPs
Purchasing power by cities/districts of the CPs **[Kippist et al. 2011]** – average per inhabitant in € (2024) **[MBR 2024]**	After the mystery calls	Exact measurement by assigning the purchasing power data of the cities/districts to the corresponding CPs
Call duration **[Ditmars et al. 2019]**	During the mystery calls	Exact measurement based on the display of the MCs‘ telephones
Questioning score **[Collins et al. 2017]**	After the mystery calls	Exact measurement by summing the dichotomous ratings of essential question items 2, 3, 5, and 6 (range: 0–4 points)

Table 3*. Potential influencing factors, time and type of data collection.*
Note: * The potential influencing factors were taken from the respective literature sources.

Data collection

The allocation of the selected CPs to the MCs was carried out randomly. Each of the eight MCs was therefore assigned 47 CPs (376 CPs / 8 MCs = 47 CPs). In addition to the main study, a pretest with a total of 40 validation calls were initially conducted. The distribution of five validation calls per MC was likewise determined randomly. Subsequently, a workshop was held to exchange experiences and to provide mutual feedback on the specific characteristics of the scenario and the assessment form. The pretests also demonstrated the need to adapt the scenario. Specifically, the

introductory statement was modified to emphasize the MCs‘ uncertainty about whether oral EC was required and to explicitly request professional counseling from the pharmacy staff. The revised scenario was then re-tested with two validation calls per MC.

Data collection took place on different weekdays and at various times of day within a time window from 12:00 p.m. to 6:00 p.m. This scheduling was based on the scenario in which the regular pill should have been taken at 7:00 a.m. A call placed before 12:00 p.m. could have occurred too close to the missed pill intake, making it realistic that affected women would have simply taken the missed pill instead of contacting a CP. Calls after 6:00 p.m. were avoided because the 12-hour window for subsequent pill intake was nearly over, and some CPs might already have recommended oral EC for safety reasons before that period had elapsed.

Each CP was initially contacted once by its assigned MC. Mystery calls were placed from the MCs' private mobile telephones with the caller ID withheld, in order to prevent any call-backs and to avoid alerting other CPs to the study. If a CP could not be reached, the MCs made up to three call attempts at five-minute intervals on the same day. This was intended to simulate a realistic emergency situation in which, after several unsuccessful attempts, the next CP would be contacted. CPs that could not be reached were marked as unavailable, and replaced by a randomly selected CP from the same stratum.

To ensure quality assurance, a second observer [**Khojah 2019**] monitored the MC's call via the mobile telephone's speaker. Four teams consisting of two MCs each were formed, with one person serving as the second observer for the other. The second observer was responsible for entering the data into the online data collection platform Jotform (Jotform, San Francisco, USA) in parallel with the MC's conversation. Subsequently, the MC and the second observer cross-checked the digitally completed evaluation form using the four-eyes principle. Any disagreements were resolved between them. If no resolution was possible, the second observer's assessment was considered decisive. To avoid potential bias in subsequent calls, the pharmacy staff were not informed at the end of the conversation that it was a mystery call. This ensured that advance warning of other CPs was not possible.

Data management and analysis

Each two-person team conducted consistency checks on the dataset of another randomly assigned team to further ensure data quality. These checks demonstrated a very high level of data quality. Data analysis was performed using SPSS version 29 for Windows (IBM, Armonk, USA). Within descriptive statistics, frequencies and percentages were calculated for categorical data. Both the Kolmogorov-Smirnov and the Shapiro-Wilk tests indicated that the continuous data were not normally distributed. Therefore, the median, interquartile range (IQR), minimum, and maximum were calculated. In addition, 95% confidence intervals for both categorical and continuous data were reported using bootstrapping.

A binomial logistic regression model was also developed to identify the influence of various independent variables (**Table 3**) on the dichotomous AO. The independent variables showed neither outliers nor multicollinearity. Following Hosmer et al. [**2013**], variables with a p-value of less than 0.25 in the bivariate analysis were included in the multivariate analysis. Odds ratios, 95% confidence intervals, and p-values were reported. Nagelkerke's R^2 was used to assess model fit. Effect size was measured using Cohen's f^2, with values of 0.02, 0.15, and 0.35 indicating small, medium, and large effects, respectively [**Cohen 1992**]. A two-tailed p-value of less than 0.05 was considered statistically significant in all analyses.

The qualitative data were analyzed through inductive category formation and subdivision into main and subcategories. To further illustrate the findings, corresponding anchor examples were also provided [**Iversen et al. 2014; Guo et al. 2022; Liyange et al. 2024**].

Ethical approval

The study protocol was approved by the institutional ethics committee of the Neubrandenburg University of Applied Sciences (registration number: HSNB/220/24). In line with international literature [**Xu et al. 2012**], the mystery calls were conducted covertly and without the option for opt-out, in order to avoid bias from the Hawthorne effect [**Choi et al. 2019**] as well as selection bias [**Rhodes/Miller 2012**]. However, to ensure transparency, all participating CPs were notified by letter in June 2024 about the conduct of the study, adherence to ethical research principles, and the provision of general, non-pharmacy-specific feedback after completion of the research project. This procedure followed recommendations in international literature [**Rhodes/Miller 2012;**

Fitzpatrick/Tumlinson 2017] and was consistent with the implementation in numerous studies [**Kashyap et al. 2014; da Rocha et al. 2015; Mobark et al. 2019**]. Specific information about the scenario used was not disclosed in order to protect the covert study design. For the same reason, the letter indicated only a broad time frame ("The study will be conducted during the course of 2024") rather than a concrete date, so that the mystery calls were carried out at an unknown point in time from the CPs' perspective. The collected data were pseudonymized and processed in accordance with the Guideline for the Use of Mystery Research in Market and Social Research [**BVM 2022**], ensuring that individual CPs and their staff could not be identified. The authors also signed a written commitment to treat all experiences and data with strict confidentiality both during and after the study [**Fitzpatrick/Tumlinson 2017**]. The students who served as MCs likewise signed a declaration confirming their consent to act as MCs.

Results

During data collection, 23 of the 376 CPs had to be replaced. The reasons for this were as follows: inability to reach the CP after three call attempts (10 CPs), permanent closure of the CP (7 CPs), unplanned deviation from the scenario (2 CPs), closure of the CP at the time of the call (2 CPs), and a callback request outside the specified time window (1 CP). In addition, one CP was duplicated in the sample (1 CP). In the end, as planned, 376 mystery calls were successfully conducted (call completion rate: 100.0%). Characteristics of the MCs, mystery calls, pharmacy staff, and CPs are presented in **Table 4**.

	**Frequency (n)**<br>**Total (376)**	**Percentage (%)**<br>**Total (100)**
MC identity		
1	47	12.5
2	47	12.5
3	47	12.5
4	47	12.5
5	47	12.5
6	47	12.5
7	47	12.5
8	47	12.5
Mystery call attempts		
1st attempt	333	88.6
2nd attempt	37	9.8
3rd attempt	6	1.6
Time of the mystery calls		
12:00 p.m. - 3:00 p.m.	177	47.1

3:01 p.m. - 6:00 p.m.	199	52.9
Forwarding to other pharmacy staff		
No	248	66.0
Yes	128	34.0
Placed on hold after call acceptance		
No	280	74.5
≤ 1:00 min	68	18.1
> 1:00 min	28	7.4
Background noise in the CPs (e.g., other pharmacy staff conversations)		
No	359	95.5
Yes	17	4.5
Gender of the pharmacy staff		
Female	307	81.6
Male	69	18.4
Location of the CPs		
Rural	131	34.8
Urban	245	65.2
Purchasing power		
≤ €25,500	66	17.6
€25,501 - €29,500	220	58.5
≥ €29,501	90	23.9
Call duration		
≤ 1:30 min	76	20.2
1:31 - 3:00 min	144	38.3
> 3:00 min	156	41.5

Table 4. *Characteristics of MCs, mystery calls, pharmacy staff, and CPs.*

Some interlocutors terminated the telephone conversation prematurely (exit) (14.4%, 54/376), which meant that not all items of the assessment form could be completed for all 376 mystery calls. Consequently, the sample sizes for items 1 to 21 differ. The AO was achieved in 44.4% of mystery calls (143/322). In 46.0% of mystery calls (148/322), the advice was given to take the missed combined oral contraceptive. In 37.0% of mystery calls (119/322), pharmacy staff additionally emphasized doing so immediately and continuing regular intake. In contrast, a recommendation to use oral EC was given in 49.1% of mystery calls (158/322) (**Table 5**).

The median questioning score was 2.0 [IQR 1.0–3.0]. A minimum score of 0 was observed in 23.3% of mystery calls (75/322). Conversely, the maximum score of 4 was achieved in 24.5% of mystery calls (79/322), although the AO was not reached in 12.7% of these cases (10/79). Within the questioning score, the most frequently asked item concerned the reason for requesting oral EC (70.1%, 227/324). Moreover, the timing of UPSI – which was not essential for achieving the AO and therefore not part of the questioning score – was the most frequently asked question overall (71.8%, 234/326) (**Table 5**).

	Total *(n)*	Frequency *(n)*[a]	Percentage *(%)*[a]	95% CI
(1) Did the staff ask for the woman‘s age?	322	46	14.3	10.6–18.6
(2) Did the staff ask about the reason for requesting oral EC?	324	227	70.1	65.1–75.0
(3) Did the staff ask about the brand name of the pill?	322	140	43.5	37.9–48.8
(4) Did the staff ask about the sequence number of the missed pill(s)?	322	62	19.3	14.6–23.6
(5) Did the staff ask about the total number of missed pills?	323	142	44.0	39.0–49.8
(6) Did the staff ask when the last pill was taken?	323	155	48.0	42.4–53.3
(7) Did the staff ask about the timing of UPSI?	326	234	71.8	66.9–77.0
(8) Did the staff ask about the date of the last menstrual period?	325	170	52.3	47.1–57.5
Did the staff ask whether				
(9) the last menstrual period was more than 28 days ago?	322	2	0.6	0.0–1.6
(10) the last menstrual period was lighter than usual?	322	6	1.9	0.6–3.4
(11) the last menstrual period was shorter than usual?	322	5	1.6	0.3–3.1
(12) the last menstrual period was otherwise unusual?	322	15	4.7	2.5–6.8
(13) Did the staff ask whether acute health problems or chronic diseases are present?	323	36	11.1	7.7–14.9
(14) Did the staff ask whether the woman is currently breastfeeding?	322	16	5.0	2.8–7.5
(15) Did the staff ask whether any medications are being taken?	322	38	11.8	8.1–15.5
(16) Did the staff ask whether oral EC had been used previously?	323	20	6.2	3.7–9.0
(17) Did the staff recommend oral EC?	322	158	49.1	43.5–54.3
(18) Did the staff recommend that oral EC was not needed?	322	143	44.4	38.8–49.7
(19) Did the staff recommend consulting a physician?	324	29	9.0	5.9–12.0
(20) Did the staff recommend an in-person visit to the CP?	375	84	22.4	18.1–26.4
(21) Did the staff recommend the intake of the combined oral contraceptive by				
• immediately,	322	119	37.0	31.7–42.2
• taking the missed pill,	322	148	46.0	40.7–51.2
• and then continuing as scheduled?	322	119	37.0	31.7–42.2

Table 5. *Assessment items.*

[a] Results present the frequency and percentage of mystery calls with assessment “Yes”.

Table 6 presents the binary logistic regression model. In the bivariate analysis, six potential influencing factors (MC identity, mystery call attempts, forwarding to other pharmacy staff, placed on hold after call acceptance, call duration, and questioning score) showed a p-value of < 0.25. These variables were subsequently included in the multivariate logistic regression model. The analysis revealed that MC 2 (AOR = 9.88; 95% CI: 2.00–48.77), MC 3 (AOR = 6.31; 95% CI: 1.33–29.93), MC 4 (AOR = 24.19; 95% CI: 3.85–151.89), and MC 5 (AOR = 9.87; 95% CI: 2.15–45.40) were associated with a significantly higher likelihood of achieving the AO compared with MC 1. Furthermore, a significant positive association was observed between the questioning score (AOR = 5.32; 95% CI: 3.66–7.74) and the AO. All other variables showed no significant effects on the AO. The model reached a Nagelkerke R^2 value of 0.705, corresponding to a Cohen's f^2 of 2.399, indicating a "large" effect size [**Cohen 1992**].

Potential Influencing Factors	*n (%)* Total	*n (%)* Appropriate outcome	*n (%)* Inappropriate outcome	COR (95% CI)	*p*-Value	AOR (95% CI)	*p*-Value
	**322 (100)**	**143 (44.4)**	**179 (55.6)**				
MC identity							
1	41 (100)	9 (22.0)	32 (78.0)	1		1	
2	40 (100)	19 (47.5)	21 (52.5)	3.22 (1.23–8.45)	.018	9.88 (2.00–48.77)	.005*
3	44 (100)	17 (38.6)	27 (61.4)	2.24 (0.86–5.83)	.099	6.31 (1.33–29.93)	.020*
4	38 (100)	28 (73.7)	10 (26.3)	9.96 (3.54–27.99)	<.001	24.19 (3.85–151.89)	<.001*
5	40 (100)	24 (60.0)	16 (40.0)	5.33 (2.02–14.11)	<.001	9.87 (2.15–45.40)	.003*
6	40 (100)	19 (47.5)	21 (52.5)	3.22 (1.23–8.45)	.018	1.90 (0.42–8.51)	.403
7	40 (100)	13 (32.5)	27 (67.5)	1.71 (0.64–4.62)	.288	1.51 (0.33–6.89)	.598
8	39 (100)	14 (35.9)	25 (64.1)	1.99 (0.74–5.35)	.172	2.51 (0.52–12.10)	.252
Mystery call attempts							
1st attempt	281 (100)	121 (43.1)	160 (56.9)	1		1	
2nd attempt	35 (100)	19 (54.3)	16 (45.7)	1.57 (0.78–3.18)	.210	2.03 (0.65–6.30)	.221
3rd attempt	6 (100)	3 (50.0)	3 (50.0)	1.32 (0.26–6.67)	.735	0.78 (0.02–30.48)	.895
Time of the mystery calls							
12:00 p.m. - 3:00 p.m.	150 (100)	70 (46.7)	80 (53.3)	1			
3:01 p.m. - 6:00 p.m.	172 (100)	73 (42.4)	99 (57.6)	0.84 (0.54–1.31)	.447		
Forwarding to other pharmacy staff							
No	200 (100)	81 (40.5)	119 (59.5)	1		1	
Yes	122 (100)	62 (50.8)	60 (49.2)	1.52 (0.97–2.39)	.071	1.98 (0.84–4.70)	.121
Placed on hold after call acceptance							
No	231 (100)	103 (44.6)	128 (55.4)	1		1	
≤ 1:00 min	65 (100)	23 (35.4)	42 (64.6)	0.68 (0.39–1.20)	.186	1.18 (0.40–3.54)	.763
> 1:00 min	26 (100)	17 (65.4)	9 (34.6)	2.35 (1.01–5.48)	.049	1.15 (0.29–4.64)	.845
Background noise in the CPs (e.g., other pharmacy staff conversations)							
No	306 (100)	134 (43.8)	172 (56.2)	1			
Yes	16 (100)	9 (56.2)	7 (43.8)	1.65 (0.60–4.55)	.333		
Gender of the pharmacy staff							
Female	259 (100)	117 (45.2)	142 (54.8)	1			
Male	63 (100)	26 (41.3)	37 (58.7)	0.85 (0.49–1.49)	.576		
Location of the CPs							

Rural	107 (100)	46 (43.0)	61 (57.0)	1			
Urban	215 (100)	97 (45.1)	118 (54.9)	1.09 (0.68–1.74)	.718		
Purchasing power							
≤ €25,500	53 (100)	27 (50.9)	26 (49.1)	1			
€25,501 - €29,500	191 (100)	82 (42.9)	109 (57.1)	0.72 (0.39–1.33)	.300		
≥ €29,501	78 (100)	34 (43.6)	44 (56.4)	0.74 (0.37–1.50)	.408		
Call duration							
≤ 1:30 min	35 (100)	4 (11.4)	31 (88.6)	1		1	
1:31 - 3:00 min	131 (100)	42 (32.1)	89 (67.9)	3.66 (1.21–11.03)	.021	1.39 (0.33–5.90)	.655
> 3:00 min	156 (100)	97 (62.2)	59 (37.8)	12.74 (4.28–37.91)	<.001	1.53 (0.34–6.89)	.577
Questioning score [a]	2.0 (1.0–3.0)	3.0 (3.0–4.0)	1.0 (0.0–2.0)	4.63 (3.43–6.26)	<.001	5.32 (3.66–7.74)	<.001*

Table 6. *Potential influencing factors on the AO.*

[a] Median (IQR); Abbreviations: COR=Crude Odds Ratio, AOR=Adjusted Odds Ratio; *Significant at $p < 0.05$.

In the qualitative assessment of notable occurrences, a total of 97 statements made by pharmacy staff were recorded by the second observer. Through inductive categorization, four main categories and eleven subcategories were derived. On the one hand, the data revealed positive aspects of counseling, including the use of checklists and guidelines, the provision of additional information on contraception, and requests for callbacks following the pharmacy staff’s thematic preparation. On the other hand, several statements pointed to deficiencies in counseling, including refusal to provide telephone counseling, misinformation, inappropriate remarks and morally questionable comments, as well as unfriendliness on the part of the pharmacy staff. A detailed overview of all categories, including anchor examples, is provided in **Table 7**.

Main categories	Subcategories	(Mystery calls No.) anchor examples
Counseling outcomes and recommendations	Recommendations for taking oral EC	(255) Paraphrased: "Better to take the morning-after pill once too often than once too little."
	Recommendations against taking oral EC	(216) Based on the information that the first day of the last menstrual period was 9 days ago and sexual intercourse occurred the previous evening, it was stated that nothing could happen and that the morning-after pill was not necessary.
	No clear recommendation	(70) No clear recommendation was made, leaving the decision to the MC.
Knowledge transfer and education	Information on price and mode of action of oral EC	(306) The MC was informed about different types of morning-after pills and their prices.
	Additional contraceptive advice	(016) Paraphrased: "After taking the morning-after pill, also use condoms."
	Misinformation	(179) Paraphrased: "The 12-hour window for taking the pill has already passed; it has already been 24 hours."
Organizational aspects of counseling	Use of checklists and guidelines	(226) Pharmacy staff went through the BAK checklist point by point.
	Refusal to provide telephone counseling	(265) Paraphrased: "We do not do that over the telephone – go to a gynecologist or come in. I will definitely not do this over the telephone."
	Follow-up questions / callback requests	(023) A call-back was requested so that the pharmacy staff could take time to read the package leaflet.
Inappropriate communication	Inappropriate remarks / morally questionable comments	(022) Paraphrased: "We sold the morning-after pill nine times on Sunday while on duty. It's our bestseller when we're on duty!“
	Unfriendliness on the part of the pharmacy staff	(244) Paraphrased: The pharmacy staff sigh when told that the MC has not yet read the package leaflet: “And you didn't take the pill again?!” When the caller responds: “No”, the staff ask: “Why not?” and “Why didn't you call this morning?”

Table 7. *Evaluation of the qualitative item with main categories, subcategories and anchor examples.*

Discussion

Appropriate outcome

The AO – in this case, not recommending oral EC – was achieved in just under half of all mystery calls. In contrast, a nationwide survey among pharmacists from CPs in Germany reported that, in a comparable scenario, oral EC was not dispensed in 56.3% of cases [**Said et al. 2019**]. This higher value is not surprising [**Surur et al. 2017; Mengistu et al. 2019**] and is likely attributable to the survey design, which is prone to social desirability bias [**Collins et al. 2021**], as well as to voluntary CP participation (selection bias) [**Rhodes/Miller 2012**].

An Australian SPM visit study using a scenario in which dispensing of oral EC was not indicated due to a missed inactive regular pill near the end of the menstrual cycle reported a substantially lower AO of 24.0% [**Schneider et al. 2013**]. This is surprising, as the pharmacy staff in that study asked considerably more questions than in the present investigation. In another Australian SPM visit study by Smith et al. [**2019**], using a scenario in which dispensing of oral EC was not indicated because the effective time window for oral EC had elapsed, the AO – i.e., refraining from dispensing – occurred in only 20.5% of cases. Differences in study design and counseling context may account for these discrepancies.

In this MC study, oral EC was explicitly recommended in 49.1% of cases, which is problematic in view of the potential financial [**Elmes et al. 2022; Torres et al. 2026**], physical [**Salcedo et al. 2023; Li et al. 2024**], and psychosocial consequences for affected women [**Eastham et al. 2020; Chan et al. 2021; Szajbely et al. 2025**]. This is particularly concerning given that German citizens place substantial trust in counseling provided in CPs [**DAZ 2024**].

The specific reasons for the poor results cannot be determined using an MC approach. Therefore, further research employing other methods, such as questionnaires and interviews, is required. However, as demonstrated in the literature, several factors contributing to inadequate counseling quality can be inferred. Internationally, these include insufficient knowledge, lack of time and information among pharmacy staff, as well as limited human resources [**Horvat et al. 2012; Ayalew 2017; Surur et al. 2017; Ayele et al. 2018**]. In contrast, pharmacy staff in Germany reported in a comparatively

older study that they were concerned that customers might feel patronized by counseling [**Berger et al. 2005**]. More recent studies additionally identified uncertainties [**Said et al. 2019**] and concerns among pharmacy staff that customers might feel uncomfortable when asked numerous questions during counseling [**Seiberth et al. 2022**]. Limited customer interest and time, as well as insufficient information about customers, have further been cited as reasons in the national literature [**Schumacher et al. 2019; Seiberth et al. 2020; Seiberth et al. 2022**]. However, this contrasts with survey findings showing that counseling is often desired, even when customers do not actively request it [**Seiberth et al. 2021**].

Interventions for improvement

Considering that the AO was achieved in only 44.4% of mystery calls, as well as the uncertainties, unclear recommendations, and incorrect statements identified in the qualitative item of pharmacy staff, targeted training represents an effective measure to enhance relevant professional competencies [**Gastelurrutia et al. 2013; Al Aqeel et al. 2018; Seiberth et al. 2020**].

A heightened awareness in dealing with affected women should also be fostered in training programs in order to counteract inappropriate remarks by pharmacy staff, which were likewise identified in the qualitative item. A central focus should be on destigmatizing oral EC and preventing statements, behaviors, or value-laden attitudes that may lead to shame, stigma, or perceived judgment among the women concerned [**Eastham et al. 2020; Chan et al. 2021; Szajbely et al. 2025**]. In this context, appropriate communication should already be practiced during vocational training for pharmaceutical technical assistants and pharmacy degree programs [**Langer et al. 2016; Seiberth et al. 2020; Nona et al. 2025**].

Another possible intervention involves the use of a checklist [**Al Aqeel et al. 2018; Pro Familia Bundesverband 2018; Haag et al. 2019; Langer et al. 2020; Nona et al. 2025**], which should be readily available during telephone consultations. This is further supported by the significant positive association between the questioning score and the AO. This association underscores the importance of asking questions. Nevertheless, the findings also demonstrate that even asking essential questions does not necessarily prevent incorrect conclusions and thus the failure to achieve the AO. Moreover, empirical evidence from a German SPM visit study indicates that the BAK

checklist is used in practice in only about half of counseling interactions [**Langer et al. 2020**], and some pharmacy staff perceive it as too extensive, overly complex, or insufficiently practice-oriented [**Bruhns 2015**]. Accordingly, in addition to an explicit recommendation for the checklist's use within the BAK guideline [**Langer et al. 2020**], an adaptation of this guideline also appears warranted to promote the continuous optimization of counseling practices [**Pro Familia Bundesverband 2018; Seiberth et al. 2020**].

In this context, for example, the term "personal" counseling should be specified more clearly to indicate that such counseling can also take place over the telephone. The need for this clarification is supported by the overall study results, as both the quantitative and qualitative findings show that telephone counseling frequently led to a premature end of the consultation (exit).

In addition to the aspects already discussed, the issue of access to CPs must also be addressed. Several CPs could not be reached even after three call attempts. This represents a barrier to patient care, particularly when medications are urgently needed, such as oral EC. The state chambers of pharmacists should therefore encourage CPs to ensure unrestricted telephone accessibility in light of their important role in patient care.

Influencing factors

The regression analysis revealed a significant association between the AO and some of the MCs. Nationally and internationally, there are no studies with a comparable scenario that have identified such an association, which prevents direct comparison of the results [**Yaffe et al. 2012**]. Because the data were collected through mystery calls and all eight MCs followed a standardized scenario, it is reasonable to assume that the pharmacy staff could perceive only the MCs' voices and last names as distinguishing features.

This raises the question of how the voices of four MCs differ compared with a reference MC. Possible differences between voices may include audibility, clarity, pace, or warmth [**Alnezary et al. 2024**]. The voice is therefore a highly individual human characteristic and can vary considerably from person to person [**Klenk 2020**]. Furthermore, in communication, vocal quality and speaking style are often attributed

to greater importance than the actual content of what is said [**Guyer et al. 2019**]. In general, higher-pitched voices are perceived more quickly than lower-pitched ones. However, lower-pitched voices tend to be perceived as more trustworthy and competent, whereas higher-pitched voices are often associated with childlike or emotional qualities [**Amon 2003; Tsantani et al. 2016**]. To identify specific vocal factors that may facilitate a counseling conversation, further research using additional methods – such as questionnaires or interviews – would be required. In addition to the voice, it is also conceivable that the MCs' last names contributed to the significant association observed between some MCs and the AO. However, the literature has not yet examined any relationship between last names and the quality of counseling. For this reason, further research is warranted in this area.

Consistent with other international SPM visit studies [**Collins et al. 2017; Langer et al. 2020**], the number of questions asked had a significant influence on the AO. This shows that the correct decision – in this case, not recommending oral EC – can only be made when essential questions are asked and, consequently, relevant information about the MC is obtained. Among the four essential questions (items 2, 3, 5, 6), the reason for the request (item 2) was asked most frequently – analogous to the Australian SPM visit study with a highly similar scenario, in which it was asked in 96.0% of cases [**Schneider et al. 2013**]. In addition, the question about the timing of the UPSI (item 7) was the most frequently asked question overall. SPM visit studies conducted in Switzerland, Belgium, and Australia reported somewhat higher scenario-dependent rates – 100.0% [**Haag et al. 2019**], 96.1% [**Vander Steen et al. 2024**], 95.0% [**Schneider et al. 2013**], and 88.0% [**Collins et al. 2018**] – compared with this study (78.1%). This illustrates the basic reflex of pharmacy staff to ask this question [**Ceulemans et al. 2022**]. Such a question is crucial in other scenarios that focus on the supply of oral EC. For example, the corresponding value in the German SPM visit study is 93.5% [**Langer et al. 2020**]. This shows that the question is often asked even when it is not relevant (as in our scenario). To ensure that the appropriate recommendation is given for each scenario, it is essential that pharmacy staff ask all questions specified in the BAK checklist.

Strengths and limitations

The data were collected using the "gold standard" [**Collins et al. 2021; Kunow/Langer 2024**], the SPM, applied here in the form of mystery calls. This approach avoids both the Hawthorne effect and social desirability bias. Furthermore, for the first time worldwide – and thus also for the first time in Germany – an MC-based approach was used to examine whether oral EC is being recommended unnecessarily in CPs. Selection bias was avoided because CPs had no possibility to opt out.

In addition, measures were taken to ensure that the MCs behaved as uniformly as possible during the mystery calls to avoid any distortion of the results. As is customary in SPM studies [**Collins et al. 2021**], this was achieved through standardized MC responses during the mystery calls, as well as through extensive MC training in the form of multiple pretests and a dedicated workshop. Another strength is the use of a second observer, which minimizes faulty recall during the call and thereby largely rules out recall bias [**Saba et al. 2014; Kunow et al. 2021**].

A notable limitation is that pharmacy staff terminated the mystery call prematurely in 54 cases (exit). While the target sample of 376 mystery calls was achieved, exits led to reduced sample sizes for individual analyses. Other scenarios (e.g., a symptom-based scenario or one requiring a mandatory recommendation to consult a physician) could lead to different results, even in cases of identical indication [**Horvat et al. 2012; Langer et al. 2018a**].

The pharmacy-specific performance feedback recommended in the international literature [**Xu et al. 2012**] was not provided in this MC study, as the very large sample size – compared with SPM visit studies – would have made this approach highly labor-intensive. Instead, the participating CPs will receive general, non-pharmacy-specific feedback in the form of a notification that the study results have been published in an international, peer-reviewed journal. Furthermore, it should be noted that counseling provided during mystery calls is likely not delivered to the same extent as during in-person visits [**Stone et al. 2020**]. One possible explanation is that pharmacy staff may assume that the MC would have to pick up the morning-after pill at the CP anyway, making telephone counseling unnecessary [**Kunow/Langer 2023**]. In addition, mystery calls involved only one member of the pharmacy staff, implying that other staff members might have provided different counseling [**Langer et al. 2018b**].

With regard to the additional collection of qualitative results, it should be noted that the content of some categories (“inappropriate remarks/morally questionable statements“, “unfriendliness on the part of the pharmacy staff“) could only be assessed subjectively. Moreover, the qualitative data were not collected systematically but only when the MC and the second observer judged an event to be noteworthy. Furthermore, no measures were taken to examine whether pharmacy staff recognized the mystery calls. Such an investigation would have required cooperation with the CPs [**Obreli-Neto et al. 2013**], which was incompatible with the study design (no opt-out). Nevertheless, none of the CPs indicated that they had recognized the calls as mystery calls.

Conclusion

The results indicate insufficient information gathering and a lack of appropriate recommendations. For this reason, the consistent use and explicit recommendation of the BAK checklist during telephone consultations should be considered in the relevant guideline. Appropriate training for pharmacy staff may help address professional and communicative deficits. Despite numerous standardization measures, some MCs were surprisingly shown to have a significant influence on the AO. There is a need for further research, particularly regarding the influence of the MCs’ voice and last name.

DECLARATIONS

Data Availability

All relevant data are within the manuscript.

Funding

We acknowledge support for the Article Processing Charge from the Open Access Publication Fund of the Neubrandenburg University of Applied Sciences.

Conflicts of Interest

The authors have declared that no competing interests exist.

Author Contributions

Conceptualization: Bernhard Langer, Anne Beck, Antonia Marie Dreier, Rosa Gabrieljan, Hanna Hilgenhof, Johanna Latuske, Carolin Priepke, Katharina Schaihadinow, Helene Schwarz.

Data curation: Bernhard Langer, Anne Beck, Antonia Marie Dreier, Rosa Gabrieljan, Hanna Hilgenhof, Johanna Latuske, Carolin Priepke, Katharina Schaihadinow, Helene Schwarz.

Formal analysis: Bernhard Langer, Anne Beck, Antonia Marie Dreier, Rosa Gabrieljan, Hanna Hilgenhof, Johanna Latuske, Carolin Priepke, Katharina Schaihadinow, Helene Schwarz.

Investigation: Anne Beck, Antonia Marie Dreier, Rosa Gabrieljan, Hanna Hilgenhof, Johanna Latuske, Carolin Priepke, Katharina Schaihadinow, Helene Schwarz.

Methodology: Bernhard Langer, Anne Beck, Antonia Marie Dreier, Rosa Gabrieljan, Hanna Hilgenhof, Johanna Latuske, Carolin Priepke, Katharina Schaihadinow, Helene Schwarz.

Project administration: Bernhard Langer.

Validation: Bernhard Langer, Anne Beck, Antonia Marie Dreier, Rosa Gabrieljan, Hanna Hilgenhof, Johanna Latuske, Carolin Priepke, Katharina Schaihadinow, Helene Schwarz, Christian Kunow.

Visualization: Bernhard Langer, Anne Beck, Antonia Marie Dreier, Rosa Gabrieljan, Hanna Hilgenhof, Johanna Latuske, Carolin Priepke, Katharina Schaihadinow, Helene Schwarz.

Writing – original draft: Bernhard Langer, Anne Beck, Antonia Marie Dreier, Rosa Gabrieljan, Hanna Hilgenhof, Johanna Latuske, Carolin Priepke, Katharina

Schaihadinow, Helene Schwarz.

Writing – review & editing: Bernhard Langer, Anne Beck, Antonia Marie Dreier, Rosa Gabrieljan, Hanna Hilgenhof, Johanna Latuske, Carolin Priepke, Katharina Schaihadinow, Helene Schwarz, Christian Kunow.

References

1. **Abajobir** AA, Alati R, Kisely S, Najman JM. Antecedents and maternal health outcomes of unintended pregnancy: a systematic review. Ethiopian Medical Journal. 2017;55(4):325–54.
2. **ABDA** – Federal Union of German Associations of Pharmacists. German Pharmacies. Figures, Data, Facts 2024. 2024. https://www.abda.de/fileadmin/user_upload/assets/ZDF/Zahlen-Daten-Fakten-24/ABDA_ZDF_2024_Brosch_english.pdf (accessed March 24, 2026).
3. **ABDA** – Federal Union of German Associations of Pharmacists. German Pharmacies. Figures, Data, Facts 2025. 2025. https://www.abda.de/fileadmin/user_upload/assets/ZDF/Jahrbuch-ZDF-2025/ABDA_ZDF_2025_Brosch_english.pdf (accessed March 24, 2026).
4. **Al Aqeel** S, Abanmy N, AlShaya H, Almeshari A. Interventions for improving pharmacist-led patient counselling in the community setting: a systematic review. Systematic Reviews. 2018;7(1):71. https://doi.org/10.1186/s13643-018-0727-4
5. **Alnezary** FS, Alahmadi DA, Abduljaleel FN, Alharbi RG, Alzahrani F, Almutairi MS. Assessing Community Pharmacists' Management of Acute Diarrhea in Saudi Arabia: A Simulated Patient Study. Healthcare (Basel). 2024;12(23):2385. https://doi.org/10.3390/healthcare12232385
6. **Alte** D, Weitschies W, Ritter CA. Evaluation of consultation in community pharmacies with mystery shoppers. Ann Pharmacother. 2007;41(6):1023–30. https://doi.org/10.1345/aph.1H565
7. **Amon** I. Voice power — using your voice for successful communication. Elektrotech Inftech. 2003;120:242–4. https://doi.org/10.1007/BF03054881
8. **AMVV** – Arzneimittelverschreibungsverordnung. Arzneimittelverschreibungsverordnung vom 21. Dezember 2005 (BGBl. I S. 3632), die zuletzt durch Artikel 1 der Verordnung vom 9. Oktober 2025 (BGBl. 2025 I Nr. 236) geändert worden ist. (2025). https://www.gesetze-im-internet.de/amvv/AMVV.pdf (accessed March 24, 2026).
9. **ApBetrO** – Apothekenbetriebsordnung. Apothekenbetriebsordnung in der Fassung der Bekanntmachung vom 26. September 1995 (BGBl. I S. 1195), die zuletzt durch Artikel 8z4 des Gesetzes vom 12. Dezember 2023 (BGBl. 2023 I Nr. 359) geändert worden ist (2023). https://www.gesetze-im-internet.de/apobetro_1987/ApBetrO.pdf (accessed March 24, 2026).
10. **ApoG** – Apothekengesetz. Apothekengesetz in der Fassung der Bekanntmachung vom 15. Oktober 1980 (BGBl. I S. 1993), das zuletzt durch Artikel 3 des Gesetzes vom 22. Dezember 2025 (BGBl. 2025 I Nr. 356) geändert worden ist (2025). https://www.gesetze-im-internet.de/apog/ApoG.pdf (accessed March 24, 2026).
11. **Ayalew** M. Medication Counseling Practice in Ethiopia: A Systematic Review. J Basic Clin Pharma. 2017;8:S001–S005.
12. **Ayele** AA, Mekuria AB, Tegegn HG. Gebresillassie BM, Mekonnen AB, Erku DA. Management of minor ailments in a community pharmacy setting: Findings from

simulated visits and qualitative study in Gondar town, Ethiopia. PLoS One. 2018;13(1): e0190583. https://doi.org/10.1371/journal.pone.0190583

13. **BAK** – Bundesapothekerkammer. Rezeptfreie Abgabe von oralen Notfallkontrazeptiva („Pille danach“). Handlungsempfehlungen der Bundesapothekerkammer. 2021. https://www.abda.de/fileadmin/user_upload/assets/Praktische_Hilfen/Leitlinien/Selbstmedikation/BAK_Handlungsempfehlungen-Checkliste-NFK_20180228_Anpassung_20211123.pdf (accessed March 24, 2026).
14. **BBSR** – Bundesinstitut für Bau-, Stadt- und Raumforschung. BBSR Raumgliederungen: Gebietsstand 31.12.2022. 2023. https://www.bbsr.bund.de/BBSR/DE/forschung/raumbeobachtung/Raumabgrenzungen/downloads/download-referenzen.html (accessed March 24, 2026).
15. **Bearak** J, Popinchalk A, Ganatra B, Moller A-B, Tunçalp Ö, Beavin C, et al. Unintended pregnancy and abortion by income, region, and the legal status of abortion: estimates from a comprehensive model for 1990-2019. Lancet Glob Health. 2020;8(9):e1152–e1161. https://doi.org/10.1016/s2214-109x(20)30315-6
16. **Berger** K, Eickhoff C, Schulz M. Counselling quality in community pharmacies: implementation of the pseudo customer methodology in Germany. J Clin Pharm Ther. 2005;30(1):45–57. https://doi.org/10.1111/j.1365-2710.2004.00611.x
17. **Björnsdottir** I, Granas A, Bradley A, Norris P. A systematic review of the use of simulated patient methodology in pharmacy practice research from 2006 to 2016. Int J Pharm Pract. 2020;28(1):13–25. https://doi.org/10.1111/ijpp.12570
18. **BMG** – Bundesministerium für Gesundheit. Emergency contraception: what you need to know about the “morning after” pill. 2021. https://gesund.bund.de/en/emergency-contraception (accessed March 24, 2026).
19. **Brache** V, Cochon L, Deniaud M, Croxatto HB. Ulipristal acetate prevents ovulation more effectively than levonorgestrel: analysis of pooled data from three randomized trials of emergency contraception regimens. Contraception. 2013;88(5):611–8. https://doi.org/10.1016/j.contraception.2013.05.010
20. **Bruhns** C. Ergebnisse einer bundesweiten Befragung zur aktuellen Abgabepraxis der Pille danach. In: Pro familia Bundesverband. Rezeptfreie Pille danach – Abgabepraxis und Information. 2015. 14–20. https://www.profamilia.de/fileadmin/publikationen/Fachpublikationen/doku_pille__danach-2016_web.pdf (accessed March 24, 2026).
21. **BVM** – Berufsverband Deutscher Markt- und Sozialforscher e.V. Richtlinie für den Einsatz von Mystery Research in der Markt- und Sozialforschung. 2022. https://www.bvm.org/fileadmin/user_upload/Verbandsdokumente/Standesregeln_RL_neu_2021/Richtlinie_fuer_den_Einsatz_von_Mystery_Research_2022.pdf (accessed March 24, 2026).
22. **Ceulemans** M, Brughmans M, Poortmans LL, Spreuwers E, Willekens J, Roose N, et al. Development and Pilot Testing of a Dispensing Protocol on Emergency Contraceptive Pills for Community Pharmacists in Belgium. Pharmacy (Basel). 2022;10(3):58. https://doi.org/10.3390/pharmacy10030058

23. **Chan** MC, Munro S, Schummers L, Albert A, Mackenzie F, Soon JA, et al. Dispensing and practice use patterns, facilitators and barriers for uptake of ulipristal acetate emergency contraception in British Columbia: a mixed-methods study. CMAJ Open. 2021;9(4):E1097–E1104. https://doi.org/10.9778/cmajo.20200193
24. **Cheng** A, Kessler D, Mackinnon R, Chang TP, Nadkarni VM, Hunt E, et al. Simul Healthc. 2016;11(4):238–48. https://doi.org/10.1097/SIH.0000000000000150
25. **Choi** WJ, Jung JJ, Grantcharov TP. Impact of Hawthorne effect on healthcare professionals: a systematic review. Univ Tor Med J. 2019;96(2):21–32.
26. **Cohen** J. A power primer. Psychol Bull. 1992;112(1):155–9. https://doi.org/10.1037//0033-2909.112.1.155
27. **Collins** JC, Schneider CR, Naughtin CL, Wilson F, de Almeida Neto AC, Moles RJ. Mystery shopping and coaching as a form of audit and feedback to improve community pharmacy management of non-prescription medicine requests: An intervention study. BMJ Open. 2017;7(12):e019462. https://doi.org/10.1136/bmjopen-2017-019462
28. **Collins** JC, Schneider CR, Moles RJ. Emergency contraception supply in Australian pharmacies after the introduction of ulipristal acetate: a mystery shopping mixed-methods study. Contraception. 2018;98(3):243–6. https://doi.org/10.1016/j.contraception.2018.05.003
29. **Collins** JC, Chong WW, de Almeida Neto AC, Moles RJ, Schneider CR. The simulated patient method: Design and application in health services research. Res Social Adm Pharm. 2021;17(12):2108–15. https://doi.org/10.1016/j.sapharm.2021.04.021
30. **Da Rocha** CE, Bispo ML, dos Santos ACO, Mesquita AR, Brito GC, de Lyra DP Jr. Assessment of community pharmacists' counseling practices with simulated patients who have minor illness: a pilot study. Simul Healthc. 2015;10(4):227–38. https://doi.org/10.1097/SIH.0000000000000100
31. **DAZ** – Deutsche Apotheker Zeitung. Apotheken sind unverzichtbar für sichere Selbstmedikation. 2024. http://disq.us/t/4sl2av3 (accessed March 24, 2026).
32. **Deutscher Apotheker Verlag**. Bundes-Apotheken-Register. 2024. https://www.deutscher-apotheker-verlag.de/Bundes-Apotheken-Register/141015000 (accessed March 24, 2026).
33. **Dierolf** V, Freytag S. Zugang zur Pille danach in den Apotheken nach der Rezeptfreigabe. Pro Fam Mag. 2017;45(4):9–12.
34. **Ditmars** L, Rafie S, Kashou G, Cleland K, Bayer L, Wilkinson TA. Emergency contraception counseling in California community pharmacies: a mystery caller study. Pharmacy (Basel). 2019;7(2):38. https://doi.org/10.3390/pharmacy7020038
35. **Eastham** R, Milligan C, Limmer M. Qualitative findings about stigma as a barrier to contraception use: the case of Emergency Hormonal Contraception in Britain and implications for future contraceptive interventions. Eur J Contracept Reprod Health Care. 2020;25(5):334–8. https://doi.org/10.1080/13625187.2020.1806998

36. **Elmes** S, Yarger J, Blum M, De La Melena S, Van Liefde D, Hopkins K, et al. Cost barriers to emergency contraception among community college students. Contraception Posters. APHA 2022 Annual Meeting and Expo. https://apha.confex.com/apha/2022/meetingapi.cgi/Session/65586?filename=2022_Session65586.pdf&template=Word (accessed March 24, 2026).
37. **Fitzpatrick** A, Tumlinson K. Strategies for Optimal Implementation of Simulated Clients for Measuring Quality of Care in Low- and Middle-Income Countries. Glob Health Sci Pract. 2017;5(1):108–14. https://doi.org/10.9745/GHSP-D-16-00266
38. **Gastelurrutia** MA, Larrañaga B, Garay A, Echeveste FA, Fernandez-Llimos F. Impact of a program to reduce the dispensing of antibiotics without a prescription in Spain. Pharm Pract (Granada). 2013;11(4):185–90. https://doi.org/10.4321/s1886-36552013000400002
39. **Glaeske** G, Thürmann P. Pillenreport. Ein Statusbericht zu oralen Kontrazeptiva. https://www.tk.de/resource/blob/2043476/e640ad05778a48b6e868a9bbce7922b7/studienband-pillenreport-2015-data.pdf (accessed March 24, 2026).
40. **Glasier** A, Baraitser P, McDaid L, Norrie J, Radley, A, Stephenson JM, et al. Emergency contraception from the pharmacy 20 years on: a mystery shopper study. BMJ Sex Reprod Health. 2021;47(1):55–60. https://doi.org/10.1136/bmjsrh-2020-200648
41. **Guo** H, Hildon Z-J, Chow A. "Antibiotics are for everyone, our past and our future generations, right? If antibiotics are dead, we will be in big trouble": Building on community values for public engagement on appropriate use of antibiotics in Singapore. Front Public Health. 2022;10:1001282. https://doi.org/10.3389/fpubh.2022.1001282
42. **Guttmacher Institute**. Germany. 2025. https://www.guttmacher.org/regions/europe/germany (accessed March 24, 2026).
43. **Guyer** J, Fabrigar LR, Vaughan-Johnston TI. Speech Rate, Intonation, and Pitch: Investigating the Bias and Cue Effects of Vocal Confidence on Persuasion. Pers Soc Psychol Bull. 2019;45(3):389–405. https://doi.org/10.1177/0146167218787805
44. **Haag** M, Gudka S, Hersberger KE, Arnet I. Do Swiss community pharmacists address the risk of sexually transmitted infections during a consultation on emergency contraception? A simulated patient study. Eur J Contracept Reprod Health Care. 2019;24(5):407–12. https://doi.org/10.1080/13625187.2019.1661377
45. **Hernandez** JH, Mbadu MF, Garcia M, Glover A. The provision of emergency contraception in Kinshasa's private sector pharmacies: experiences of mystery clients. Contraception. 2018;97(1):57–61. https://doi.org/10.1016/j.contraception.2017.08.001
46. **Horvat** N, Koder M, Kos M. Using the simulated patient methodology to assess paracetamol-related counselling for headache. PLoS One. 2012;7(12):e52510. https://doi.org/10.1371/journal.pone.0052510

47. **Hosmer** Jr DW, Lemeshow S, Sturdivant RX. Applied Logistic Regression. Hoboken, New Jersey, Wiley 3rd ed. 2013.
48. **Hussainy** SY, Stewart K, Pham M-P. A mystery caller evaluation of emergency contraception supply practices in community pharmacies in Victoria, Australia. Aust J Prim Health. 2015;21(3):310–6. https://doi.org/10.1071/PY14006
49. **Iversen** HH, Bjertnæs OA, Skudal KE. Patient evaluation of hospital outcomes: an analysis of open-ended comments from extreme clusters in a national survey. BMJ Open. 2014;4(5):e004848. https://doi.org/10.1136/bmjopen-2014-004848
50. **Jenapharm** (n. d.). Unser Produktportfolio. https://www.jenapharm.de/unternehmen/produkte (accessed March 24, 2026).
51. **Kashour** TS, Joury A, Alotaibi AM, Althagafi M, Almufleh AS, Hersi A, et al. Quality of assessment and counselling offered by community pharmacists and medication sale without prescription to patients presenting with acute cardiac symptoms: a simulated client study. Eur J Clin Pharmacol. 2016;72(3):321–8. https://doi.org/10.1007/s00228-015-1981-1
52. **Kashyap** KC, Nissen LM, Smith SS, Kyle G. Management of over-the-counter insomnia complaints in Australian community pharmacies: a standardized patient study. Int J Pharm Pract. 2014;22(2):125–34. https://doi.org/10.1111/ijpp.12052
53. **Khojah** HMJ. Privacy level in private community pharmacies in Saudi Arabia: A simulated client survey. Pharmacology & Pharmacy. 2019;10(10):445–55. https://doi.org/10.4236/pp.2019.1010036
54. **Kiechle** M, Neuenfeldt M. Experience with oral emergency contraception since the OTC switch in Germany. Arch Gynecol Obstet. 2017;295(3):651–60. https://doi.org/10.1007/s00404-016-4253-0
55. **Kippist** C, Wong K, Bartlett D, Saini B. How do pharmacists respond to complaints of acute insomnia? A simulated patient study. Int J Clin Pharm. 2011;33(2):237–45. https://doi.org/10.1007/s11096-011-9482-5
56. **Klenk** M. Stimme, Sprechen, Hören: Von der sinnlich-sinnhaften Verfertigung wissenschaftlicher Erkenntnis beim Sprechen und Hören. kommunikation@gesellschaft. 2020;21(2):1–28. https://doi.org/10.15460/kommges.2020.21.2.623
57. **Kunow** C, Bello MA, Diedrich L, Eutin L, Sonnenberg Y, Wachtel N, et al. A Nationwide Mystery Caller Evaluation of Oral Emergency Contraception Practices from German Community Pharmacies: An Observational Study Protocol. Healthcare (Basel). 2021;9(8):945. https://doi.org/10.3390/healthcare9080945
58. **Kunow** C, Langer B. Dispensing and Variabilities in Pricing of Headache OTC Medicines by Community Pharmacies in a German Big City: A Simulated Patient Approach. Clinicoecon Outcomes Res. 2021;13:843–51. https://doi.org/10.2147/ceor.s320728
59. **Kunow** C, Langer B. Using the simulated patient methodology to assess the quality of counselling in German community pharmacies: a systematic review

from 2005 to 2018. Int J Pharm Pharm Sci. 2021;13(1):10–9. http://dx.doi.org/10.22159/ijpps.2021v13i1.40034

60. **Kunow** C, Langer B. Using the Simulated Patient Methodology in the Form of Mystery Calls in Community Pharmacy Practice Research: A Scoping Review Protocol. Pharmacy (Basel). 2023;11(2):47. https://doi.org/10.3390/pharmacy11020047
61. **Kunow** C, Langer B. Simulated patient methodology as a "gold standard" in community pharmacy practice: Response to criticism. World J Methodol. 2024;14(2):93026. https://doi.org/10.5662/wjm.v14.i2.93026
62. **Kunow** C, Langer B. Simulated patient methodology for the investigation of community pharmacy practice: a worldwide success story. J Med Life. 2025;18(9):914–5. https://doi.org/10.25122/jml-2025-0138
63. **Kunow** C, Priepke C, Langer B. Pricing of oral emergency contraception by German community pharmacies – a nationwide mystery caller investigation. Med Pharm Rep. 2025;98(4):535–41. https://doi.org/10.15386/mpr-2860
64. **Langer** B, Bull E, Burgsthaler T, Glawe J, Schwobeda M, Simon K. Using the simulated patient methodology to assess counselling for acute diarrhoea - evidence from Germany. Z Evid Fortbild Qual Gesundhwes. 2016;112:19–26. http://dx.doi.org/10.1016/j.zefq.2016.02.001
65. **Langer** B, Bull E, Burgsthaler T, Glawe J, Schwobeda M, Simon K. Assessment of counselling for acute diarrhoea in German pharmacies: a simulated patient study. Int J Pharm Pract. 2018a;26(4):310–7. https://doi.org/10.1111/ijpp.12405
66. **Langer** B, Kieper M, Laube S, Schramm J, Weber S, Werwath A. Assessment of counselling for acute diarrhoea in North-Eastern German pharmacies—A follow-up study using the simulated patient methodology. Pharmacology & Pharmacy. 2018b;9(7):257–69. https://doi.org/10.4236/pp.2018.97020
67. **Langer** B, Kunow C. Medication dispensing, additional therapeutic recommendations, and pricing practices for acute diarrhoea by community pharmacies in Germany: a simulated patient study. Pharm Pract (Granada). 2019;17(3):1579. https://doi.org/10.18549/PharmPract.2019.3.1579
68. **Langer** B, Grimm S, Lungfiel G, Mandlmeier F, Wenig V. The quality of counselling for oral emergency contraceptive pills - A simulated patient study in German community pharmacies. Int J Environ Res Public Health. 2020;17(18):6720. https://doi.org/10.3390/ijerph17186720
69. **Langer** B, Kunow C, Bolduan J, Sackmann L, Schreiter L, Schüler K, et al. Counselling with a focus on product and price transparency for over-the-counter headache medicines: A simulated patient study in community pharmacies in Munich, Germany. Int J Health Plann Manage. 2024;39(5):1434–55. https://doi.org/10.1002/hpm.3817
70. **Langer** B, Kunow C, Dethloff T, George S. Medication Recommendation, Counseling, and Pricing for Nasal Sprays in German Community Pharmacies: A Simulated Patient Investigation. Adv Respir Med. 2025;93(3):18. https://doi.org/10.3390/arm93030018

71. **Li** RHW, Lo SST, Cameron ST. Hormonal methods for emergency contraception. Best Pract Res Clin Obstet Gynaecol. 2024;97:102550. https://doi.org/10.1016/j.bpobgyn.2024.102550
72. **Liyange** V, Low XR, Park JS, Ngo HC, Clifford R, Seubert L. Exploring the usability of simulated patient methodology in dental clinics in Western Australia: A pilot survey. Clin Exp Dent Res. 2024;10(4):e906. https://doi.org/10.1002/cre2.906
73. **Lungfiel** G, Mandlmeier F, Kunow C, Langer B. Oral emergency contraception practices of community pharmacies: a mystery caller study in the capital of Germany, Berlin. J Pharm Policy Pract. 2023;16(1):68. https://doi.org/10.1186/s40545-023-00565-w
74. **Madson** MJ, Gade Y, Srivastava U. Who's there? Mystery calling for UX research. Journal of User Experience. 2025;20(2):52–64.
75. **MBR** – Michael Bauer Research GmbH. Kaufkraft 2023 in Deutschland. Stadt- und Landkreise – wichtigste Variablen. 2024. https://www.mb-research.de/_download/MBR-Kaufkraft-Kreise.pdf (accessed March 24, 2026).
76. **Mengistu** G, Gietnet K, Amare F, Sisay M, Hagos B, Misganaw D. Self-Reported and Actual Involvement of Community Pharmacy Professionals in the Management of Childhood Diarrhea: A Cross-Sectional and Simulated Patient Study at two Towns of Eastern Ethiopia. Clin Med Insights Pediatr. 2019;13:1179556519855380. https://doi.org/10.1177/1179556519855380
77. **Mobark** DM, Al-Tabakha MM, Hasan S. Assessing hormonal contraceptive dispensing and counseling provided by community pharmacists in the United Arab Emirates: a simulated patient study. Pharm Pract (Granada). 2019;17(2):1465. https://doi.org/10.18549/PharmPract.2019.2.1465
78. **Nelson** HD, Darney BG, Ahrens K, Burgess A, Jungbauer RM, Cantor A, et al. Associations of Unintended Pregnancy With Maternal and Infant Health Outcomes: A Systematic Review and Meta-analysis. JAMA. 2022;328(17):1714–29. https://doi.org/10.1001/jama.2022.19097
79. **Nona** RA, Ray RA, Taylor SM, Glass BD. Knowledge, attitudes, and practices of community pharmacists providing over-the-counter emergency hormonal contraception: a scoping review. Int J Pharm Pract. 2025;33(1):6–18. https://doi.org/10.1093/ijpp/riae062
80. **O'Neil** ML, Aldanmaz B. Identity and the Availability of Emergency Contraception from Pharmacies in Istanbul. Kadın/Woman 2000 J Women's Stud. 2021;21(2):35–45. https://doi.org/10.33831/jws.v22i2.318
81. **Obare** F, Keesbury J, Liambila W. The provision of emergency contraceptives in private sector pharmacies in urban Kenya: Experiences of mystery clients. Afr Popul Stud. 2013;24(1-2):42–52. https://doi.org/10.11564/24-1-2-305
82. **Obreli-Neto** PR, Pereira LR, Guidoni CM, de Oliveira Baldoni A, Marusic S, de Lyra-Júnior DP, et al. Use of simulated patients to evaluate combined oral contraceptive dispensing practices of community pharmacists. PloS one. 2013;8(12):e79875. https://doi.org/10.1371/journal.pone.0079875

83. **Odukoya** OK, Chui MA. Using Think Aloud Protocols to Assess E- Prescribing in Community Pharmacies. Innov Pharm. 2012;3(3):88. https://doi.org/10.24926/iip.v3i3.270
84. **Park** JS, Page A, Clifford R, Bond C, Seubert L. Refining the CRiSPHe (checklist for reporting research using a simulated patient methodology in Health): a Delphi study. Int J Pharm Pract. 2024;32(4):322–8. https://doi.org/10.1093/ijpp/riae019
85. **Pro familia Bundesverband**. Pille danach rezeptfrei: Zugang ohne Hürden? Nutzerrinnenbefragung zur Vergabepraxis in Apotheken. 2018. https://www.profamilia.de/fileadmin/publikationen/Fachpublikationen/Verhuetung/Pille_danach-Zugang-ohne_huerden.pdf (accessed March 24, 2026).
86. **Rabe** T, Albring C, Ahrendt H-J, Mueck AO, Merkle E, König, et al. Postcoital contraception—an update. Gynecologic Endocrinol. 2013;11(3):197–202. https://doi.org/10.1007/s10304-012-0533-3
87. **Rabe** T, Merkle E, Albring C, Bitzer J, Egarter C, Hinney B, et al. The Basics of Emergency Contraception by means of typical cases. J Reproduktionsmed Endokrinol. 2017;14(2):57–74.
88. **Rankin** KA, Mosier-Mills A, Hsiang W, Wiznia DH. Secret shopper studies: an unorthodox design that measures inequities in healthcare access. Arch Public Health. 2022;80(1):226. https://doi.org/10.1186/s13690-022-00979-z
89. **Raosoft**. Sample size calculator by Raosoft. 2004. http://www.raosoft.com/samplesize.html (accessed April 30, 2024).
90. **Renner** I. Informationsstand zur Pille danach – Ergebnisse einer bundesweiten repräsentativen Befragung erwachsener Frauen. In: Pro familia Bundesverband. Rezeptfreie Pille danach – Abgabepraxis und Information. 2015. 11–4. https://www.profamilia.de/fileadmin/publikationen/Fachpublikationen/doku_pille__danach-2016_web.pdf (accessed March 24, 2026).
91. **Rhodes** KV, Miller FG. Simulated patient studies: an ethical analysis: Simulated patient studies: An ethical analysis. Milbank Q. 2012;90(4):706–24. https://doi.org/10.1111/j.1468-0009.2012.00680.x
92. **Saba** M, Diep J, Bittoun R, Saini B. Provision of smoking cessation services in Australian community pharmacies: A simulated patient study. Int J Clin Pharm. 2014;36(3):604–14. https://doi.org/10.1007/s11096-014-9944-7
93. **Said** A, Ganso M, Freudewald L, Schulz M. Trends in dispensing oral emergency contraceptives and safety issues: a survey of German community pharmacists. Int J Clin Pharm. 2019;41(6):1499–506. https://doi.org/10.1007/s11096-019-00911-6
94. **Salcedo** J, Cleland K, Bartz D, Thompson I. Society of Family Planning Clinical Recommendation: Emergency contraception. Contraception. 2023;121:109958. https://doi.org/10.1016/j.contraception.2023.109958
95. **Saxena** P, Mishra A, Nigam A. Evaluation of pharmacists' services for dispensing emergency contraceptive pills in Delhi, India: A mystery shopper study. Indian J Community Med. 2016;41(3):198–202. https://doi.org/10.4103/0970-0218.183595

96. **Scharmanski** S, Hessling A. Notfallkontrazeption. Jugendsexualität 9. Welle. BZgA-Faktenblatt Köln: Bundeszentrale für gesundheitliche Aufklärung (BZgA). 2021. https://doi.org/10.17623/BZgA_SRH:fb_JUS9_Not (accessed March 24, 2026).
97. **Schneider** C, Gudka S, Fleischer L, Clifford R. The use of a written assessment checklist for the provision of emergency contraception via community pharmacies: a simulated patient study. Pharm Pract (Granada). 2013;11(3):127–31. https://doi.org/10.4321/s1886-36552013000300001
98. **Schumacher** PM, Neininger MP, Kaune A, Bertsche T. Counseling patients on correct drug handling in German community pharmacies: experiences and opinions of pharmaceutical staff. Int J Clin Pharm. 2019;41(1):151–8. https://doi.org/10.1007/s11096-018-0757-y
99. **Seiberth** JM, Moritz K, Kücükay N, Schiek S, Bertsche T. What is the attitude towards and the current practice of information exchange during self-medication counselling in German community pharmacies? An assessment through self-report and non-participant observation. PLoS One. 2020;15(10):e0240672. https://doi.org/10.1371/journal.pone.0240672
100. **Seiberth** JM, Moritz K, Vogel CF, Bertsche T, Schiek S. Public's perspectives on guideline-recommended self-medication consultations in German community pharmacies. Health Soc Care Community. 2021;29(1):194–205. https://doi.org/10.1111/hsc.13082
101. **Seiberth** JM, Moritz K, Herrmann NS, Bertsche T, Schiek S. What influences the information exchange during self-medication consultations in community pharmacies? A non-participant observation study. Res Social Adm Pharm. 2022;18(3):2444–56. https://doi.org/10.1016/j.sapharm.2021.03.015
102. **Shen** J, Che Y, Showell E, Chen K, Cheng L. Interventions for emergency contraception. Cochrane Database Syst Rev. 2019;1(1):CD001324. https://doi.org/10.1002/14651858.cd001324.pub6
103. **Smith** H, Whyte S, Chan HF, Kyle G, Lau ETL, Nissen LM, et al. Pharmacist Compliance With Therapeutic Guidelines on Diagnosis and Treatment Provision. JAMA Netw Open. 2019;2(7):e197168. https://doi.org/10.1001/jamanetworkopen.2019.7168
104. **Stone** RH, Rafie S, Ernest D, Scutt B. Emergency contraception access and counseling in urban pharmacies: A comparison between states with and without pharmacist prescribing. Pharmacy (Basel). 2020;8(2):105. https://doi.org/10.3390/pharmacy8020105
105. **STROBE Statement**. STROBE Statement - Checklist of items that should be included in reports of cross-sectional studies. 2025. https://www.strobe-statement.org/fileadmin/Strobe/uploads/checklists/STROBE_checklist_v4_cross-sectional.pdf (accessed March 24, 2026).
106. **Suarez** SS, Pacey AA. Sperm transport in the female reproductive tract. Hum Reprod Update. 2006;12(1):23–37. https://doi.org/10.1093/humupd/dmi047
107. **Surur** AS, Getachew E, Teressa E, Hailemeskel B, Getaw NS, Erku DA. Self-reported and actual involvement of community pharmacists in patient

counseling: a cross-sectional and simulated patient study in Gondar, Ethiopia. Pharm Pract (Granada). 2017;15(1):890. https://doi.org/10.18549/PharmPract.2017.01.890

108. **Szajbely** K, Neiterman E. Exploring the Experiences of Ontario Postsecondary Students With the Emergency Contraception Pill. J Obstet Gynaecol Can. 2025;47(6):102825. https://doi.org/10.1016/j.jogc.2025.102825
109. **Tavares** MP, Foster AM. Emergency contraception in a public health emergency: exploring pharmacy availability in Brazil. Contraception. 2016;94(2):109–14. https://doi.org/10.1016/j.contraception.2016.04.006
110. **Torres** E, Elliott S, Carter G, Woodcock AL, Mullholand M, Turok DK. Enhancing access to emergency contraception: Results from a no-cost program in Utah. Contraception. 2026;154:111288. https://doi.org/10.1016/j.contraception.2025.111288
111. **Torres** NF, Solomon VP, Middleton LE. Pharmacists' practices for non-prescribed antibiotic dispensing in Mozambique. Pharm Pract (Granada). 2020;18(3):1965. https://doi.org/10.18549/pharmpract.2020.3.1965
112. **Tsantani** MS, Belin P, Paterson HM, McAleer P. Low Vocal Pitch Preference Drives First Impressions Irrespective of Context in Male Voices but Not in Female Voices. Perception. 2016;45(8):946–63. https://doi.org/10.1177/0301006616643675
113. **Uysal** J, Tavrow P, Hsu R, Alterman A. Availability and accessibility of emergency contraception to adolescent callers in pharmacies in four Southwestern States. J Adolesc Health. 2019;64(2):219–25. https://doi.org/10.1016/j.jadohealth.2018.08.030
114. **Uzun** GD, Sancar M, Okuyan B. Evaluation of knowledge and attitude of pharmacist and pharmacy technicians on emergency contraception method in Istanbul, Turkey: A simulated patient study. J Res Pharm. 2019;23(3):395–402. https://doi.org/10.12991/jrp.2019.147
115. **van der Linde** M, du Plessis SS. Idiopathic Infertility: Survival and Function of Sperm in the Female Reproductive Tract. In: Schattman GL, Esteves SC, Agarwal A. Unexplained Infertility. Pathophysiology, Evaluation and Treatment. Springer, New York. 2015. 43–51.
116. **Vander Steen** G, Ropers J, Rousseau C, Joris A, Gilles C, Rozenberg S, et al. Pharmacist recommendations for emergency contraception in Belgium: a simulated user study. Eur J Contracept Reprod Health Care. 2024;29(4):177–81. https://doi.org/10.1080/13625187.2024.2345114
117. **WHO** – World Health Organization. Emergency Contraception. 2021. https://www.who.int/news-room/fact-sheets/detail/emergency-contraception (accessed March 24, 2026).
118. **Wilkinson** TA, Rafie S, Clark PD, Carroll AE, Miller E. Evaluating community pharmacy responses about levonorgestrel emergency contraception by mystery caller characteristics. J Adolesc Health. 2018;63(1):32–6. https://doi.org/10.1016/j.jadohealth.2017.11.303
119. **Xu** T, de Almeida Neto AC, Moles RJ. A systematic review of simulated-patient methods used in community pharmacy to assess the provision of non-

prescription medicines. Int J Pharm Pract. 2012;20(5):307–19. https://doi.org/10.1111/j.2042-7174.2012.00201.x

120. **Yaffe** J, Montgomery P, Hopewell S, Shepard LD. Empty reviews: a description and consideration of Cochrane systematic reviews with no included studies. PLoS One. 2012;7(5):e36626. https://doi.org/10.1371/journal.pone.0036626

121. **Yazdkhasti** M, Pourreza A, Pirak A, Abdi F. Unintended Pregnancy and Its Adverse Social and Economic Consequences on Health System: A Narrative Review Article. Iran J Public Health. 2015;44(1):12–21.

122. **Zapata-Cachafeiro** M, Piñeiro-Lamas M, Guinovart MC, López-Vázquez P, Vázquez-Lago JM, Figueiras A. Magnitude and determinants of antibiotic dispensing without prescription in Spain: a simulated patient study. J Antimicrob Chemother. 2019;74(2):511–4. https://doi.org/10.1093/jac/dky440
