# Supplementary material for: Do community pharmacies unnecessarily recommend oral emergency contraception? A nationwide mystery caller investigation in Germany
Source: PLoS One. 2026 Oct 23;21(10):e0361027. doi: 10.1371/journal.pone.0361027 (PMC13693899; doi:10.1371/journal.pone.0361027)
Supplement: Supplementary file 2 [file pone.0361027.s001.docx]

**Reviewer Comments**

| **S/N** | **SECTION** | **DESCRIPTION** |
| --- | --- | --- |
| 1 | **Introduction** | - The Author puts much details on pharmacology (LNG vs UPA timing), about scenario logic (missed pill, sperm survival, etc.) These could sound better more appropriately in the methodological part especially on the scenario section - Limited critical engagement with the guidelines as the Author presented the use of **BAK guideline** as the standard but you briefly mention ambiguity when you write “ personal counselling” however, the Author did not explore fully if the guidelines itself has limitation or variability in interpretation - There is a limited discussion of the existing SPM in Germany as the Author cited two studies one study conducted in Germany and another conducted in Europe, but it could sound more better if the Author could indicate why the author considers those studies are insufficient and what exactly your study adds methodologically - The introduction is very rich in information but very dense. Some sentences are very long, heavy referencing and there are multiple ideas per paragraph. All these can reduce clarity for readers and affect readability. - Also there are some repetition ( e.g, counseling importance). |
|  |  |  |
| 2 | **Methods** | Generally on Methods   - The description is well detailed but somehow very dense, therefore consider:   - Breaking long sentences into shorter sentences   - Separating sampling, validation and replacement procedures more clearly - Also, some sections are overly detailed especially on pharmacological explanation, which might be better suited for background and not methods. - The description could be streamlined for clarity and flow - Avoid heavy abbreviation use early on (e.g., SPM, MCs, CP ) unless clearly defined once and used consistently. - Ensure consistent formatting of references and guideline names. - Deficiency of clear limitation discussion   - The Limitations are handled **covertly through design choices,** but not **explicitly acknowledged** - Generally the Methodology is **strong in design and execution**, but:   - Some **important biases are insufficiently addressed or acknowledged**   However, the author need to address the following concern;   - Interviewer (MC) effect   - MC identity significantly affects the outcomes. This is a critical limitation, not just a findings, as it indicates measurement bias but also suggest lack of full standardization as the Author did not indicate clustering adjustment any where. Therefore interviewer effect not adequately controlled   - Although CPs were randomly assigned to MCs and each MC handles 47 calls, this is likely to create a risk of systematic differences between callers interms of tone, confidence and communication style. Therefore there is a need for cluster effects adjustement and inter – caller variability analysis - Limited mystery callers (MC) diversity   - All MCs were female, young 21- 24 years, and students in health - related field. This may introduce potential **systematic bias** and limits generalizability of the results and may influence the responses - Single scenario design   - The Author use only one scenario/ clinical case. This limits the scope of inference and the ability to generalize across EC situation. - Replacement and non – response bias   - The Author declared that the “CPs were replaced if unreachable” but the Author did not report any response rate and how many CPs were replaced and Whether unreachable CPs differ systematically   - Replacement strategies may introduce selection bias if replacement differ systematically as the author using additional random draws within the strata   - The author did not report of **how often the replacement occurred** and no analysis of potential bias   - Also, the author did not indicate the reasons for non-contact - Observer and measurement bias - According to the report the second observer seems were not blinded as they knows the study aims, records data in real time and also has the final decision in disagreements which may leads to systematic bias risk and Potential **observer expectancy bias** - Lack of reliability metrics   - The Author did not comments anything regarding inter – rater reliability and also, on inter – caller consistency measures - Reliance on google for validation   - This approach may not be fully reliable or standardized as it could introduce information bias, therefore Author may consider to acknowledge this as a limitation - Limited calling window   - The author restrict calls 12.00pm – 6.00pm which is justified clinically, but; it may exclude morning staff, with difference experience levels and late hours practices which may limit generalizability across working hours - The author declared that “ if CP was unreachable the MCs made up three calls attempt at 5 minutes interval on the same day. (three – attempt rule). This may not fully reflect real patient behavior and reduce natural variability in help – seeking behavior - Insufficient details in qualitative analysis   - The author mention Inductive category formation but information regarding: coding procedure (number of coders), reliability ( e.g intercoder agreement, and software (if used) are missing leads to weakens the trustworthiness of the qualitative findings - No clear handling of clustering in statistical analysis   - The data are clustered but analysis assumes independent observation, which may leads to underestimate standard errors and Inflate statistical significance   - Clustering not addressed statistically as the data are nested ( calls within MCs) if ignored may lead to bias standard error and affect significance testing - Detection bias   - The author declared some mitigation but no discussion of whether calls were suspected - Inconsistency in sample size reporting   - The author state “ 376 CPs are sampled but only 322 CPs included in analysis and you do not explain the drop either non-response or exclusion which raises the question about selection bias and data completeness - Slight over interpretation in conclusions   - The author recommend the use of checklist and training, these are reasonable, but; the study is observational (cross-sectional), therefore causal should be cautious - Improve neutrality and precision in interpretation |
| 3 | **RESULTS** | Generally, this is a comprehensive and data-rich results section with:   - Clear reporting of sample flow and exclusions - Robust descriptive statistics - Appropriate regression analysis - Inclusion of qualitative findings   However, the needs to clarify on the some aspects stipulated below:   - Critical inconsistency “100% call completion rate versus replacements. “ “376 mystery calls were successfully conducted (call completion rate: 100.0%)”   - **The Author reported earlier that 23 CPs replaced, 54 exits, duplication and deviations. This makes 100% completion rate misleading** - Minor inconsistency in denominators   Such as:   - 70.1% (227/324) - 71.8% (234/326) - This indicates varying missing data per item but not clearly explained - Qualitative results are too compressed. The Author summarize 97 statement to 4 categories but; there is no proportion of positive versus negative categories and also no weighting of the themes |
|  |  |  |
| 4 | **General comments** | - Clear and relevant research question - Good sample size justification - Strong sampling and study design - Incredible use of SPM methodology - Good statistical analysis - High practical relevance |
|  |  |  |
