# Supplementary material for: Do community pharmacies unnecessarily recommend oral emergency contraception? A nationwide mystery caller investigation in Germany
Source: PLoS One. 2026 Oct 23;21(10):e0361027. doi: 10.1371/journal.pone.0361027 (PMC13693899; doi:10.1371/journal.pone.0361027)
Supplement: Supplementary file 3 [file pone.0361027.s002.docx]

**Reviewer Feedback**

**Manuscript Number: PONE-D-26-20770**

**Title: Do community pharmacies unnecessarily recommend oral emergency contraception? A nationwide mystery caller investigation in Germany**

**General Evaluation**

This is a well-designed national mystery-caller study using a recognised and robust methodology. The study addresses an important aspect of emergency contraception (EC) counselling in community pharmacies and provides useful information about guideline adherence and information gathering by pharmacy staff.

The manuscript could be strengthened by explaining more clearly why this research question is clinically important. The discussion could also place the findings in a broader reproductive health context and provide a more balanced overview of the role of community pharmacists in contraceptive care. In addition to avoiding unnecessary EC use, pharmacy consultations may provide opportunities to improve contraceptive adherence, prevent unintended pregnancy, and discuss long-term contraceptive options.

**Major Comments**

I offer these comments as constructive suggestions rather than a criticism of the study. The authors have undertaken a substantial amount of work using a robust methodology to address an important aspect of emergency contraception provision.

My suggestions are to direct the manuscript to more clearly highlight the broader public health relevance of the findings. This may help engage a wider readership, including policy makers, gynaecologists, family planning providers, and others interested in reducing unintended pregnancy rates.

1. Clinical importance of the study

The authors should explain more clearly why they chose to study unnecessary recommendations of emergency contraception rather than failure to provide emergency contraception when it is indicated.

The introduction highlights that 37% of pregnancies in Germany are unintended. Readers may therefore wonder why the main focus is on avoiding unnecessary EC rather than ensuring that women who need EC receive it.

Please explain the clinical importance of this research question and how it complements previous studies in this area.

The authors have previously published work examining situations where EC was indicated. It may be useful to briefly mention this work and explain how the present study investigates a different but complementary aspect of pharmacy practice.

2. Broader contraceptive counselling

The study scenario involves a woman who has forgotten a combined oral contraceptive pill.

This consultation could be viewed as an opportunity not only to determine whether EC is required, but also to discuss contraceptive adherence and future pregnancy prevention.

The discussion could briefly consider whether pharmacists can use these consultations to:

- improve pill-taking adherence;
- discuss reasons for missed pills;
- promote long-acting reversible contraception (LARC);
- support long-term reproductive health.

3. Balance of the discussion

At times, the discussion focuses mainly on deficiencies in pharmacy practice.

While identifying areas for improvement is important, it may also be helpful to acknowledge the positive role pharmacists play in improving access to contraception and emergency contraception.

The authors may wish to consider literature relating to:

- pharmacist-led contraceptive services;
- pharmacist prescribing initiatives;
- patient satisfaction with pharmacy-based contraceptive care;
- improved access to timely emergency contraception through community pharmacies.

This may provide a more balanced perspective.

4. Scope of practice

Some pharmacy staff recommended referral to a physician or gynaecologist.

The discussion mainly attributes these findings to knowledge or training gaps. Could the authors also consider whether some pharmacists may view emergency contraception counselling as outside their professional scope of practice?

Literature on pharmacists' attitudes toward emergency contraception, both in Germany and internationally, may be relevant.

**Minor Comments**

Abstract

1. Consider explaining more clearly why unnecessary recommendation of emergency contraception is clinically important.
2. The title focuses on unnecessary recommendation of EC. However, the results are presented as the percentage achieving the appropriate outcome (44.4%).

It may be easier for readers if the authors also report the percentage of consultations in which EC was recommended unnecessarily.

Introduction

3. Lines 98–106

The authors discuss the role of emergency contraception in preventing unintended pregnancy.

It may be useful to briefly discuss whether easier access to EC through community pharmacies has been shown to improve reproductive health outcomes or reduce unintended pregnancy rates.

4. Line 108

The discussion of the Yuzpe method may not be essential because this method is no longer routinely recommended in many countries due to lower efficacy and a higher rate of side effects.

Consider shortening this section and focusing on currently recommended EC methods.

It may also be useful to briefly describe the side-effect profile of current EC regimens, as this would help readers understand the rationale for avoiding unnecessary EC use.

5. Lines 118–119

The increase in over-the-counter EC use is interesting.

The authors may wish to discuss whether this increase is mainly due to improved access, financial factors, or other influences.

6. Lines 145–148

Consider including the frequency of common adverse effects of levonorgestrel and ulipristal acetate. This would help readers assess the clinical relevance of unnecessary exposure.

7. Line 158

Please clarify in which country the cited study was conducted.

Methods

8. Lines 270–274

This section may require clarification because it appears inconsistent with earlier statements that EC was not indicated in the study scenario.

9. Table 4

Please define "purchasing power" for readers who may not be familiar with this term.

Discussion

10. The discussion would benefit from additional information regarding pharmacists' attitudes toward providing emergency contraception and whether perceived scope-of-practice limitations may influence counselling behaviour.
11. Is there literature regarding the frequency of missed contraceptive pills?

Pharmacists may be aware that typical-use failure rates are substantially higher than perfect-use failure rates. This may influence counselling behaviour and may partly explain why some pharmacists recommend EC as a precaution.

12. The discussion on empathetic and non-judgemental counselling is important and should be retained.

The qualitative findings support the need to reduce stigma and judgement during EC consultations.

13. If continuing professional development (CPD) is required for pharmacists in Germany, the authors may wish to discuss whether EC counselling could be included within ongoing professional education programmes.

Conclusion

14. Future research could explore the role of digital decision-support tools, mobile applications, or AI-assisted counselling systems that use guideline-based checklists.

Such tools may improve consistency of information gathering and adherence to emergency contraception guidelines.

The authors may also wish to discuss whether there is existing evidence regarding eHealth or mHealth interventions for emergency contraception counselling.
