# Supplementary material for: Do community pharmacies unnecessarily recommend oral emergency contraception? A nationwide mystery caller investigation in Germany
Source: PLoS One. 2026 Oct 23;21(10):e0361027. doi: 10.1371/journal.pone.0361027 (PMC13693899; doi:10.1371/journal.pone.0361027)
Supplement: Supplementary file 4 [file pone.0361027.s005.docx]

Additional Editor Comments (if provided):

Requesting authors to address comments and suggestions from the reviewer and submit a revised version of the manuscript.

**Dear Editor,**

**Thank you for your comments and for handling our manuscript. Please find our point-by-point responses to the reviewers’ comments below in bold. All page and line numbers refer to the Word document with Track Changes enabled.**

Reviewer #1:

This manuscript addresses an important public health and pharmacy practice question: whether German community pharmacies recommend oral emergency contraception when it is not indicated.

**We sincerely thank the reviewer for the positive overall assessment of our study. We greatly appreciate the reviewer’s careful evaluation and constructive comments. We have addressed each of the issues raised in detail below and revised the manuscript accordingly where appropriate. We believe that these revisions have further improved the clarity and quality of the manuscript.**

Find below comments to improve the manuscript

1. Sample size calculation used P=0.5 (maximum variance), which is appropriate for an unknown proportion. However, with an observed AO of 44%, the margin of error around this estimate should be reported.

**We thank the reviewer for this comment. The precision of the observed AO estimate is already reported in Table 5 as a 95% confidence interval (AO: 44.4%; 95% CI: 38.8–49.7). As 95% confidence intervals are consistently reported for all assessment items in Table 5 and directly quantify the uncertainty around the respective estimates, we have retained this consistent presentation rather than additionally reporting a margin of error for the AO in the main text.**

2. The study reports 376 completed mystery calls, but the primary outcome analysis is based on 322 calls, as 54 were terminated prematurely. This is a major analytic decision and requires clearer justification.

The authors should explain whether excluding these 54 “exit” calls could bias the estimate of the appropriate outcome. In real-world care, premature refusal or inability to counsel is itself a clinically relevant counseling outcome, especially for emergency contraception. Excluding these cases may overestimate the proportion of appropriate recommendations.

**We thank the reviewer for raising this important point. We agree that excluding the 54 prematurely terminated calls may have affected the estimated proportion of appropriate outcomes. We now explicitly acknowledge in the Strengths and limitations section that, because these premature terminations may not have occurred at random, selection bias in the final analytic sample cannot be completely excluded (page 30, lines 682–683).**

**At the same time, classifying all exits as inappropriate outcomes is not straightforward. The BAK guideline recommends “personal” counseling, but does not clearly define whether this term refers exclusively to in-person counseling or also includes telephone counseling. This ambiguity is already explicitly addressed in the Introduction (page 6, lines 135–141). If telephone counseling is considered to fall within the BAK guideline’s concept of “personal” counseling, the exits may reasonably be regarded as a failure to achieve the AO, and their exclusion could indeed result in an overestimation of the AO, as suggested by the reviewer. Conversely, if “personal” counseling is interpreted as requiring an in-person consultation, the AO cannot be assessed for these prematurely terminated calls.**

**To address this uncertainty quantitatively, we have added a sensitivity analysis in which all 54 exits were alternatively treated as inappropriate outcomes (page 16, lines 391–394). Under this assumption, the proportion of mystery calls achieving the AO decreased from 44.4% (143/322) to 38.0% (143/376) (page 19, lines 457–459). We have also incorporated the interpretation of this sensitivity analysis and its relationship to the ambiguity of “personal” counseling into the Discussion (page 28, lines 598–604).**

The manuscript reports that 376 mystery calls were conducted successfully and that the call completion rate was 100.0%. Yet 54 calls ended prematurely, and 23 pharmacies had to be replaced. This could be misunderstood. Please define “completion rate” precisely. A flow diagram would greatly improve transparency: sampled pharmacies, excluded/replaced pharmacies, attempted calls, reached pharmacies, premature exits, and the final analytic sample.

**We thank the reviewer for this helpful comment. We agree that the term “call completion rate” could be misunderstood, particularly in light of the prematurely terminated calls. We have therefore replaced this term with “target sample achieved,” indicating that all 376 planned mystery calls were successfully conducted after replacements. We have also clarified that 54 calls (14.4%) were classified as exits, resulting in a final analytic sample of 322 calls (85.6%). In addition, we have added a flow diagram (Figure 1) showing the target population, stratified random sample, replaced CPs, successfully conducted mystery calls, exits, and final analytic sample (page 18, Figure 1).**

3. Similarly, oral EC recommendation, physician referral, and in-person visit recommendation should be clearly distinguished between “not asked/not reached because call ended” and true “No.” A sensitivity analysis treating exits as inappropriate outcomes would be useful.

**We agree that premature call exits should not be interpreted as a true “No.” However, these calls were not included in the primary analysis of oral EC recommendation, physician referral, or recommendation of an in-person visit. The primary analyses were restricted to the 322 calls that were completed sufficiently to permit assessment of these outcomes. Thus, “No” represents an observed absence of the respective recommendation and does not include calls in which the relevant point was not reached because the call ended prematurely. To assess the potential impact of the excluded premature exits, we additionally performed the requested sensitivity analysis in which these exits were classified as inappropriate outcomes.**

4. The ethical reporting requires more detail. The study used covert mystery calls without opt-out, although pharmacies were notified in general terms. This may be ethically acceptable, but the authors should provide more detail on safeguards.

**Thank you for this important comment. We agree that the safeguards implemented in the context of the covert mystery caller design should be reported in sufficient detail. Several safeguards were already described in the manuscript, including prior ethics approval, advance notification of all participating CPs, pseudonymization of the collected data, measures to prevent identification of individual CPs and their staff, and written confidentiality commitments by the authors.**

**To provide further detail, we have now expanded the Ethical approval section by specifying that access to the pseudonymized study data and the linkage list was restricted to the research team and that the data were stored securely. We have also clarified that the linkage list will be permanently deleted after publication of the study findings and distribution of the scientific publication to the contacted CPs as general, non-pharmacy-specific performance feedback (pages 17–18, lines 432–437).**

5. The manuscript reports 97 statements and presents categories and anchor examples, but the qualitative analysis is underdescribed. It is unclear who coded the data, whether more than one coder was involved, how disagreements were resolved, whether the categories were developed from transcripts/field notes, and whether any effort was made to assess coding consistency.

**Thank you for this helpful comment. We have expanded the Methods section to provide further details on the qualitative analysis, including the number of coders, the coding and category development process, the resolution of disagreements, additional validation, and the use of qualitative data analysis software (page 17, lines 405-413).**

Because the qualitative data were not systematically collected and covered only noteworthy events, the authors should avoid language that implies prevalence or comprehensive qualitative coverage. The table is useful, but the analysis should be explicitly labeled as exploratory and illustrative.

**Thank you for this important comment. We agree that the qualitative findings should not be interpreted as providing prevalence estimates or comprehensive qualitative coverage. This limitation was already explicitly acknowledged in the Limitations section, where we stated that “the qualitative data were not collected systematically but only when the MC and the second observer judged an event to be noteworthy.” To further clarify the intended scope of these findings, we have now explicitly labeled the qualitative analysis as exploratory and illustrative and revised the wording in the Results section to avoid any implication of prevalence or comprehensive coverage (page 23, line 489).**

6. The scenario hinges on a single missed combined oral contraceptive within the 12-hour window, with sexual intercourse the prior evening. The authors cite BAK guidance that oral EC is not required when the pill is taken less than 12 hours late. However, the scenario could be misunderstood by pharmacy staff if timing is not elicited precisely.
The authors should clarify whether the caller had not yet taken the missed pill at the time of the call, and whether the time of the call always ensured the 12-hour window had not elapsed. Since calls occurred between 12:00 and 18:00 and the missed pill was due at 07:00, calls near 18:00 occurred very close to the 12-hour threshold. The manuscript says calls after 18:00 were avoided because the 12-hour window was nearly over, but at 18:00, the elapsed time is already 11 hours. This should be stated more explicitly, including how calls at exactly or near 18:00 were handled.

**Thank you for this important comment. We have clarified the timing of the scenario in the manuscript. We now explicitly state that the missed combined oral contraceptive had not yet been taken at the time of the mystery call (page 11, lines 292–293). We have also clarified the handling of the 12-hour window. All mystery calls were initiated no later than 6:00 p.m. Thus, even for calls initiated at 6:00 p.m., no more than 11 hours had elapsed since the scheduled intake time of 7:00 a.m., ensuring that the MC was still within the 12-hour window. Calls were not initiated after 6:00 p.m. to avoid approaching the 12-hour threshold more closely (page 15, lines 353–357).**

7. Use one term consistently: “oral emergency contraception,” “oral EC,” or “morning-after pill.” The manuscript moves between these terms; this is understandable, but should be standardized.

**Thank you for this helpful comment. We have standardized the terminology throughout the manuscript by consistently using “oral EC” instead of “morning-after pill” after introducing the term “oral EC” earlier in the manuscript. The term “oral emergency contraception” has been retained in the title, where it refers to the contraceptive approach rather than the individual medicinal product.**

8. The finding that specific mystery callers (MC 2-5) had significantly higher odds of achieving the appropriate outcome (AORs ranging from 6.31 to 24.19) is striking, but the explanation remains speculative. The authors discuss voice characteristics and last names, but no objective voice measurements were collected.

**Thank you for this very helpful comment. We have added the following statement to the Strengths and limitations section (page 31, lines 708–717): “Although numerous measures were taken to standardize MC behavior, complete standardization of all caller characteristics across multiple MCs is virtually impossible. However, additional standardization measures could have been considered. For example, the same surname could have been used by all MCs, and voice characteristics could theoretically have been further standardized using technical voice modification. In the present study, no objective measures of voice characteristics were collected. Therefore, the contribution of objectively measurable voice characteristics to the observed mystery caller effect could not be evaluated. In addition, although MC identity was included as a fixed effect in the regression analysis, residual within-MC correlation cannot be completely excluded.”**

Reviewer #2: 

**We sincerely thank the reviewer for the careful evaluation of our study and the constructive comments. We have addressed each of the issues raised in detail below and revised the manuscript accordingly where appropriate. We believe that these revisions have further improved the clarity and quality of the manuscript.**

I would appreciate clarification regarding the following points:

Conceptual concern: The study appears to interpret the failure to follow a checklist as indicative of inappropriate practice. However, as stated in Line 133, the checklist items are neither mandatory nor explicitly recommended by the relevant guideline. Therefore, the rationale for considering non-adherence to this checklist as evidence of inappropriate recommendations requires further justification. Please clarify the basis for this assumption and provide supporting evidence from the literature or professional standards.

**We thank the reviewer for this helpful comment and would like to clarify a possible misunderstanding. In our study, failure to address individual checklist items was not interpreted as evidence of an inappropriate recommendation. Rather, two distinct aspects were assessed. First, the checklist was used to assess information gathering during counseling. The German Federal Chamber of Pharmacists explicitly describes this checklist as an instrument for quality assurance in counseling. Second, the appropriateness of the recommendation was assessed separately based on the recommendations provided in the guideline. For the scenario used in our study, the guideline explicitly states that emergency contraception is not required when a combined oral contraceptive has been taken less than 12 hours late and recommends taking the missed pill immediately and continuing regular use. Thus, classification of the recommendation as appropriate or inappropriate was based on the guideline recommendation, not on adherence to individual checklist items. We have clarified this distinction in the revised manuscript to avoid any misunderstanding (page 6, lines 141–144) by explicitly stating that the checklist was used to assess information gathering, but not to determine the appropriateness of the recommendation itself.**

Page 7, Lines 170–173: The statement “The first MC study … but did not address unnecessary recommendations” is not sufficiently clear. Please clarify what is meant by “unnecessary recommendations” and provide examples to help readers better understand this concept.

**We thank the reviewer for this comment. To clarify what the term “unnecessary recommendations” refers to, we have added “oral EC” to the respective statement. We would also like to point out that the meaning of unnecessary oral EC recommendations and a corresponding example are already provided in the preceding paragraphs. Specifically, we state that, according to the BAK guideline, oral EC is not required when a combined oral contraceptive is taken less than 12 hours late. We subsequently provide an empirical example from a German study in which pharmacists reported dispensing oral EC in cases of questionable need, including when a combined oral contraceptive had been taken less than 12 hours late. We therefore considered an additional definition or repetition of this example at the respective position unnecessary and retained the otherwise original wording to avoid redundancy.**

Page 8, Line 221: The sample size obtained using the Raosoft calculator represents the minimum required sample size. In survey based studies, it is common practice to inflate the calculated sample size (e.g., by 10–20%) to account for anticipated non-response or incomplete responses. As indicated on Page 17 (Line 398), non-response appears to have affected the sample size available for Items 1–29. Please justify the sampling strategy adopted and discuss the potential implications of the reduced sample size on the study findings.

**We thank the reviewer for this important comment and agree that the implications of the reduced analytic sample should be addressed more explicitly. At the planning stage, the calculated minimum sample size of 376 was used as the target number of mystery calls, without inflating this number to account for possible premature terminations. Although the predefined target sample of 376 mystery calls was achieved, 54 calls were terminated prematurely by pharmacy staff, resulting in a final analytic sample of 322 calls for the primary outcome. Thus, the calculated minimum sample size was not reached for the primary outcome analysis, which may have reduced the precision of the corresponding estimate. We have now explicitly acknowledged this limitation in the Strengths and limitations section. In addition, because these premature terminations may not have occurred at random, we now also acknowledge that selection bias in the final analytic sample cannot be completely excluded. Future studies should consider prospectively inflating the sample size to account for anticipated exits or drawing additional CPs until the predefined number of evaluable mystery calls is achieved (page 30, lines 682–689).**

Page 15, Line 342: The statement “These checks demonstrated a very high level of data quality” requires further support. Please provide the evidence, criteria, or results that substantiate this conclusion.

**Thank you for this very helpful comment. We have revised the Data management and analysis section to provide more specific information on the procedures used to assess data quality. In particular, we now describe the logical consistency checks performed by the research teams and clarify that no data implausibilities were identified during these checks (page 16, lines 381–384).**

Page 17, Table 4: The variable “Purchasing Power Characteristic” requires further explanation. Please clarify how this variable was calculated or categorized, what it represents, and its relevance to the study objective of investigating unnecessary recommendations of oral emergency contraception.

**Thank you for this helpful comment. As shown in Table 3, purchasing power was measured as the average purchasing power per inhabitant in 2024 and assigned according to the respective city or district in which the CP was located. We have now further clarified the categorization and rationale for including this variable. Purchasing power was categorized into three groups (≤ €25,500, €25,501–€29,500, and ≥ €29,501). The variable was included based on the hypothesis that higher local purchasing power may be associated with greater incentives for CPs to recommend comparatively expensive oral EC unnecessarily because customers may be better able to afford it, whereas lower purchasing power may be associated with lower incentives (page 14, lines 329–336).**

Reviewer #3:

**Reviewer Feedback**

**Manuscript Number: PONE-D-26-20770**

**Title: Do community pharmacies unnecessarily recommend oral emergency contraception? A nationwide mystery caller investigation in Germany**

**General Evaluation**

This is a well-designed national mystery-caller study using a recognised and robust methodology. The study addresses an important aspect of emergency contraception (EC) counselling in community pharmacies and provides useful information about guideline adherence and information gathering by pharmacy staff.

The manuscript could be strengthened by explaining more clearly why this research question is clinically important. The discussion could also place the findings in a broader reproductive health context and provide a more balanced overview of the role of community pharmacists in contraceptive care. In addition to avoiding unnecessary EC use, pharmacy consultations may provide opportunities to improve contraceptive adherence, prevent unintended pregnancy, and discuss long-term contraceptive options.

**We sincerely thank the reviewer for the positive overall assessment of our study. We greatly appreciate the reviewer’s careful evaluation and constructive comments. We have addressed each of the issues raised in detail below and revised the manuscript accordingly where appropriate. We believe that these revisions have further improved the clarity and quality of the manuscript.**

**Major Comments**

I offer these comments as constructive suggestions rather than a criticism of the study. The authors have undertaken a substantial amount of work using a robust methodology to address an important aspect of emergency contraception provision.

My suggestions are to direct the manuscript to more clearly highlight the broader public health relevance of the findings. This may help engage a wider readership, including policy makers, gynaecologists, family planning providers, and others interested in reducing unintended pregnancy rates.

1. Clinical importance of the study

The authors should explain more clearly why they chose to study unnecessary recommendations of emergency contraception rather than failure to provide emergency contraception when it is indicated.

The introduction highlights that 37% of pregnancies in Germany are unintended. Readers may therefore wonder why the main focus is on avoiding unnecessary EC rather than ensuring that women who need EC receive it.

Please explain the clinical importance of this research question and how it complements previous studies in this area.

The authors have previously published work examining situations where EC was indicated. It may be useful to briefly mention this work and explain how the present study investigates a different but complementary aspect of pharmacy practice.

**We thank the reviewer for this helpful comment. We agree that the relationship between the present research question and the complementary issue of ensuring access to oral EC when indicated should be made clear. The Introduction already describes the burden of unintended pregnancies and the importance of timely access to oral EC, while also outlining the financial, physical, and psychosocial consequences that may result from unnecessary recommendations. We therefore aimed to avoid adding repetitive information.**

**However, to make the complementary nature of our previous and present research more explicit, we revised the sentence referring to our previous German SPM visit study as follows:**

**“To date, the only SPM visit study on oral EC in Germany examined the converse scenario, in which UPA dispensing was indicated, and reported incorrect recommendations [Langer et al. 2020].”**

**This clarification emphasizes that our previous study addressed inappropriate recommendations when oral EC was indicated, whereas the present study examines inappropriate recommendations when oral EC is not indicated. Thus, the two studies investigate different but complementary aspects of appropriate oral EC counseling in community pharmacies.**

2. Broader contraceptive counselling

The study scenario involves a woman who has forgotten a combined oral contraceptive pill.

This consultation could be viewed as an opportunity not only to determine whether EC is required, but also to discuss contraceptive adherence and future pregnancy prevention.

The discussion could briefly consider whether pharmacists can use these consultations to:

- improve pill-taking adherence;
- discuss reasons for missed pills;
- promote long-acting reversible contraception (LARC);
- support long-term reproductive health.

**We thank the reviewer for this helpful suggestion. We agree that consultations concerning oral EC may also provide an opportunity to address broader contraceptive needs. We have therefore added a brief statement to the Discussion noting that such consultations may be used to reinforce adherence to regular contraception and, where appropriate, to discuss longer-term contraceptive options (page 25, lines 529-531).**

3. Balance of the discussion

At times, the discussion focuses mainly on deficiencies in pharmacy practice.

While identifying areas for improvement is important, it may also be helpful to acknowledge the positive role pharmacists play in improving access to contraception and emergency contraception.

The authors may wish to consider literature relating to:

- pharmacist-led contraceptive services;
- pharmacist prescribing initiatives;
- patient satisfaction with pharmacy-based contraceptive care;
- improved access to timely emergency contraception through community pharmacies.

This may provide a more balanced perspective.

**Thank you for this helpful suggestion. We have added a brief passage to the Discussion to provide a more balanced perspective by acknowledging the important role of community pharmacies in facilitating access to oral EC and other contraceptive services. We also emphasize that, given this important role, appropriate recommendations and high-quality counseling are essential to avoid unnecessary use. The added discussion is supported by literature already included in the manuscript [Mobark et al. 2019; Stone et al. 2020] (page 25, lines 526-529).**

4. Scope of practice

Some pharmacy staff recommended referral to a physician or gynaecologist.

The discussion mainly attributes these findings to knowledge or training gaps. Could the authors also consider whether some pharmacists may view emergency contraception counselling as outside their professional scope of practice?

Literature on pharmacists' attitudes toward emergency contraception, both in Germany and internationally, may be relevant.

**We thank the reviewer for this valuable comment. We agree that referrals to a physician or gynecologist may not necessarily be attributable solely to knowledge or training gaps. We have therefore expanded the Discussion to consider the possibility that pharmacists may perceive oral EC counseling as falling outside their professional scope of practice and linked this possible explanation to our findings. As the underlying reasons for such recommendations were not assessed, this interpretation is presented cautiously. The corresponding changes have been made on page 26, lines 545–549.**

**Minor Comments**

Abstract

1. Consider explaining more clearly why unnecessary recommendation of emergency contraception is clinically important.

**We thank the reviewer for this helpful comment. To clarify the clinical importance of unnecessary oral EC recommendations, we have revised the Background section of the Abstract to more explicitly highlight unnecessary hormonal exposure and associated side effects (page 3, lines 47–48).**

2. The title focuses on unnecessary recommendation of EC. However, the results are presented as the percentage achieving the appropriate outcome (44.4%).

It may be easier for readers if the authors also report the percentage of consultations in which EC was recommended unnecessarily.

**We thank the reviewer for this helpful comment. We agree that reporting the proportion of unnecessary oral EC recommendations in the Abstract facilitates interpretation of the findings in relation to the study title. We have therefore added that oral EC was unnecessarily recommended in 49.1% (158/322) of mystery calls (page 3, line 63).**

Introduction

3. Lines 98–106

The authors discuss the role of emergency contraception in preventing unintended pregnancy.

It may be useful to briefly discuss whether easier access to EC through community pharmacies has been shown to improve reproductive health outcomes or reduce unintended pregnancy rates.

**We thank the reviewer for this helpful comment. We agree that improved access to oral EC should be distinguished from demonstrated effects on unintended pregnancy. We have therefore added a brief statement to the Introduction noting that, although pharmacy and OTC access may facilitate more timely use of oral EC, available evidence has not demonstrated a clear reduction in unintended pregnancy compared with prescription-only access [Atkins et al. 2022]. The corresponding passage has been revised accordingly to emphasize the increasingly important role of community pharmacies in access to and counseling on oral EC (page 5, lines 125-128).**

4. Line 108

The discussion of the Yuzpe method may not be essential because this method is no longer routinely recommended in many countries due to lower efficacy and a higher rate of side effects.

Consider shortening this section and focusing on currently recommended EC methods.

**We thank the reviewer for this helpful comment. We agree that the Yuzpe method is of limited relevance in the present context. We have therefore shortened this section by removing the reference to the Yuzpe method and now focus on the currently available oral EC preparations in Germany (page 5, lines 111–113).**

It may also be useful to briefly describe the side-effect profile of current EC regimens, as this would help readers understand the rationale for avoiding unnecessary EC use.

**We thank the reviewer for this helpful comment. The side-effect profiles of the currently available oral EC regimens were already briefly addressed in the originally submitted manuscript. In response to your related comment, we have further revised the Introduction by providing frequencies of common side effects of UPA and LNG based on a randomized trial directly comparing both preparations (page 6, lines 160–163).**

Lines 118–119

The increase in over-the-counter EC use is interesting.

The authors may wish to discuss whether this increase is mainly due to improved access, financial factors, or other influences.

**We thank the reviewer for this helpful comment. We have expanded this section to clarify that the increase in oral EC use reflects improved access following the switch to OTC status and has been accompanied by a marked shift from prescription-based dispensing to self-medication [ABDA 2024]. The corresponding ABDA data show a substantial increase in OTC dispensing alongside a marked decline in prescription-based dispensing since 2015. We have also emphasized the resulting importance of CPs not only in counseling but also in providing access to oral EC (page 5, lines 123-129).**

6. Lines 145–148

Consider including the frequency of common adverse effects of levonorgestrel and ulipristal acetate. This would help readers assess the clinical relevance of unnecessary exposure.

**We thank the reviewer for this helpful suggestion. We have revised the Introduction accordingly and now provide the frequencies of common side effects of UPA and LNG based on a randomized trial directly comparing both preparations. Specifically, we report the frequencies of headache, dysmenorrhoea, nausea, and abdominal pain for UPA and LNG, respectively [Glasier et al. 2010] (page 6, lines 160–163). This allows for a more quantitative assessment of the potential clinical relevance of unnecessary exposure.**

Line 158

Please clarify in which country the cited study was conducted.

**Thank you for this helpful comment. We have clarified that the cited study was conducted in Germany by referring to it as a “German study” (page 7, line 174).**

Methods

8. Lines 270–274

This section may require clarification because it appears inconsistent with earlier statements that EC was not indicated in the study scenario.

**We thank the reviewer for this helpful comment. We agree that the original wording may have caused confusion. Oral EC was not indicated in the actual study scenario because the 12-hour window for delayed intake of the combined oral contraceptive had not yet elapsed. Rather, the intention was to construct the scenario so that, if this time window had already elapsed, the other conditions for recommending oral EC according to the BAK guideline would have been met. Specifically, the MC was in the first week of the pill cycle and sexual intercourse had taken place within the previous five days. We have revised the wording to make this distinction clearer (page 11, lines 296–300).**

9. Table 4

Please define "purchasing power" for readers who may not be familiar with this term.

**Thank you for this helpful comment. We have added a definition of purchasing power to the manuscript. Specifically, purchasing power is now defined as the disposable income of the population of a region, comprising net income after taxes and social security contributions, including received transfer payments (page 14, lines 329-336).**

Discussion

10. The discussion would benefit from additional information regarding pharmacists' attitudes toward providing emergency contraception and whether perceived scope-of-practice limitations may influence counselling behaviour.

**Thank you for this helpful comment. This issue has already been addressed in response to Major Comment 4 above. In the revised Discussion, we now discuss reported uncertainties and concerns among pharmacy staff regarding oral EC counseling and explicitly consider the possibility that some pharmacists may perceive oral EC counseling as falling outside their professional scope of practice. We also relate this possible perception to the observed recommendations to consult a physician (page 26, lines 545–549). To avoid redundancy, no additional changes were made in response to this comment.**

11. Is there literature regarding the frequency of missed contraceptive pills?

Pharmacists may be aware that typical-use failure rates are substantially higher than perfect-use failure rates. This may influence counselling behaviour and may partly explain why some pharmacists recommend EC as a precaution.

**We thank the reviewer for this helpful suggestion. We identified relevant literature showing that missed pill intake is common. In a large German survey of 18,521 women, nearly half of oral contraceptive users reported having forgotten the pill at least once during the previous three months [Oppelt et al. 2017].**

**We agree that awareness of adherence problems in everyday contraceptive use may contribute to a precautionary approach by pharmacy staff and may partly explain recommendations of oral EC even when it is not indicated in the specific situation. However, because our mystery caller study did not assess the knowledge or reasoning underlying the recommendations, we have framed this interpretation cautiously as a possible explanation.**

**The corresponding discussion has been added on page 26, lines 549–555.**

12. The discussion on empathetic and non-judgemental counselling is important and should be retained.

The qualitative findings support the need to reduce stigma and judgement during EC consultations.

**Thank you for this positive assessment. We have retained the discussion on empathetic and non-judgmental counseling and the need to reduce stigma and perceived judgment during oral EC consultations (page 27, lines 568–573).**

13. If continuing professional development (CPD) is required for pharmacists in Germany, the authors may wish to discuss whether EC counselling could be included within ongoing professional education programmes.

**Thank you for this helpful suggestion. The Discussion already emphasizes targeted training to improve professional and communication competencies in oral EC counseling. We have now additionally noted that, given that continuing professional education forms part of pharmacists’ professional obligations in Germany, oral EC counseling could also be incorporated into ongoing professional education programs (page 27, lines 576–579).**

Conclusion

14. Future research could explore the role of digital decision-support tools, mobile applications, or AI-assisted counselling systems that use guideline-based checklists.

Such tools may improve consistency of information gathering and adherence to emergency contraception guidelines.

The authors may also wish to discuss whether there is existing evidence regarding eHealth or mHealth interventions for emergency contraception counselling.

**Thank you for this valuable and forward-looking suggestion. We agree that digital decision-support tools may represent an interesting area for future research. Rather than expanding the focused Conclusion, we have added this perspective to the “Interventions for improvement” section of the Discussion (page 28, lines 611–612).**

**We have not expanded the manuscript to include a broader discussion of eHealth or mHealth interventions, as these were not examined in the present study and such a discussion would extend beyond its scope.**

Reviewer #4:

Generally, The study is very relevant with clear and relevant research question, virtuous sample size justification, strong sampling and study design, incredible use of SPM methodology, good statistical analysis and high practical relevance . however, the Author needs to address the issues raised clearly as stipulated on the attached reviewer comments file

**We sincerely thank the reviewer for the positive overall assessment of our study. We greatly appreciate the reviewer’s careful evaluation and constructive comments. We have addressed each of the issues raised in detail below and revised the manuscript accordingly where appropriate. We believe that these revisions have further improved the clarity and quality of the manuscript.**

**Reviewer Comments**

| **S/N** | **SECTION** | **DESCRIPTION** |
| --- | --- | --- |
| 1 | **Introduction** | - The Author puts much details on pharmacology (LNG vs UPA timing), about scenario logic (missed pill, sperm survival, etc.) These could sound better more appropriately in the methodological part especially on the scenario section   **We thank the reviewer for raising this point. We carefully reviewed the allocation of the pharmacological and scenario-specific information. General information on the timing and effectiveness of LNG and UPA has been retained in the Introduction because it provides relevant background on the available oral EC preparations. In contrast, the specific rationale underlying the study scenario, including the missed combined oral contraceptive, timing of sexual intercourse, sperm survival, and the 12-hour window for delayed pill intake, is described in the Scenario and Assessment section of the Methods. We also removed non-essential pharmacological detail from the Methods to further clarify this distinction. We believe that this revised allocation appropriately separates general clinical background from the methodological rationale of the specific scenario.**   - Limited critical engagement with the guidelines as the Author presented the use of **BAK guideline** as the standard but you briefly mention ambiguity when you write “ personal counselling” however, the Author did not explore fully if the guidelines itself has limitation or variability in interpretation   **We thank the reviewer for raising this point. Limitations of the BAK guideline and potential variability in its interpretation are already addressed in both the Introduction and the Discussion. In the Introduction, we note that the term “personal” counseling is insufficiently defined, particularly regarding telephone consultations, and that use of the checklist is neither mandatory nor explicitly recommended. In the Discussion, we critically revisit these issues in light of our findings. We discuss previous evidence that the checklist may be perceived as too extensive, overly complex, or insufficiently practice-oriented and therefore suggest an adaptation of the guideline. More specifically, we argue that the term “personal” counseling should be defined more clearly to indicate that counseling may also take place by telephone, a need for clarification supported by both our quantitative and qualitative findings. Taken together, these passages explicitly acknowledge both limitations of the guideline itself and the potential for variability in its interpretation and practical implementation.**   - There is a limited discussion of the existing SPM in Germany as the Author cited two studies one study conducted in Germany and another conducted in Europe, but it could sound more better if the Author could indicate why the author considers those studies are insufficient and what exactly your study adds methodologically   **We thank the reviewer for raising this point. The Introduction already explains why the available evidence is insufficient. Previous German studies did not examine correct recommendations, while the study by Said et al. was based on self-reported behavior and was therefore susceptible to social desirability bias. Furthermore, the only previous German SPM visit study on oral EC examined incorrect recommendations in situations in which UPA dispensing would have been necessary, whereas the first European MC study on oral EC identified counseling problems but did not address unnecessary recommendations. Methodologically, the present study adds a nationwide mystery caller design assessing actual counseling behavior under a standardized scenario in which oral EC was not indicated. We therefore explicitly state that, to our knowledge, no previous MC study worldwide has quantified the extent of unnecessary oral EC recommendations. Against this background, we believe that the research gap and the specific contribution of the present study are already sufficiently described in the Introduction.**   - The introduction is very rich in information but very dense. Some sentences are very long, heavy referencing and there are multiple ideas per paragraph. All these can reduce clarity for readers and affect readability.   **We thank the reviewer for this helpful comment. We carefully reviewed the Introduction and made minor editorial revisions to improve readability and reduce repetitive wording, particularly regarding the role and importance of counseling. Several longer sentences were also streamlined, and a redundant definition of the abbreviation “CP” was removed. At the same time, we retained the relevant clinical and regulatory details and references because they are important for establishing the background and rationale of the study.**   - Also there are some repetition ( e.g, counseling importance).   **We thank the reviewer for this helpful comment. We agree and addressed this point together with the preceding comment. In particular, repetitive statements concerning the importance of counseling were reduced throughout the Introduction.** |
|  |  |  |
| 2 | **Methods** | Generally on Methods   - The description is well detailed but somehow very dense, therefore consider:   - Breaking long sentences into shorter sentences   **We thank the reviewer for this helpful suggestion. We have carefully reviewed the Methods section and divided several longer sentences into shorter sentences to improve readability and clarity, while retaining the methodological detail necessary for reproducibility.**   - Separating sampling, validation and replacement procedures more clearly   **We thank the reviewer for this helpful suggestion. We have revised the Methods section to more clearly separate the sampling procedure, the preliminary verification of the operational status of sampled CPs, and the replacement procedure (page 9, lines 241-254).**   - Also, some sections are overly detailed especially on pharmacological explanation, which might be better suited for background and not methods.   **We thank the reviewer for this helpful comment. We agree that general pharmacological information is more appropriately presented in the Introduction, where such background information is already provided. We therefore reviewed the corresponding Methods section and removed the detailed information on the active ingredients and dosages of the selected combined oral contraceptive (page 10, lines 278-279). However, we retained the scenario-specific information necessary to justify the choice and plausibility of the scenario and to explain why oral EC was not indicated under the defined conditions. This information is essential for understanding and reproducing the study scenario.**   - The description could be streamlined for clarity and flow   **We thank the reviewer for this helpful suggestion. We have carefully revised the Methods section to improve clarity and flow. In particular, we streamlined several descriptions, shortened longer sentences, more clearly separated the sampling, verification, replacement, and validation procedures, and removed non-essential detail while retaining information necessary to understand and reproduce the study procedures.**   - Avoid heavy abbreviation use early on (e.g., SPM, MCs, CP ) unless clearly defined once and used consistently.   **We thank the reviewer for this helpful comment. We have reviewed the manuscript for abbreviation use and reduced the number of abbreviations in the Abstract. In particular, we now spell out “community pharmacies,” “mystery callers,” and “appropriate outcome” rather than introducing the abbreviations CPs, MCs, and AO in the Abstract. In the main text, abbreviations that are used frequently are defined at first occurrence and subsequently used consistently throughout the manuscript.**   - Ensure consistent formatting of references and guideline names.   **We thank the reviewer for this comment. We carefully reviewed the manuscript for consistent formatting of references and guideline names and corrected minor inconsistencies where necessary. Guideline and checklist names are now used consistently throughout the manuscript.**   - Deficiency of clear limitation discussion   - The Limitations are handled **covertly through design choices,** but not **explicitly acknowledged**   **We thank the reviewer for this important comment. We agree that limitations arising from specific design choices should be explicitly acknowledged rather than only being apparent from the methodological description. We have therefore substantially expanded the “Strengths and limitations” section. The revised manuscript now explicitly discusses, among other aspects, the cross-sectional design, premature termination of mystery calls, the standardized call-attempt procedure, restriction to a single scenario and a defined time window, the characteristics of the mystery callers and incomplete standardization of caller characteristics, potential observer expectancy bias, possible residual within-mystery-caller correlation, limitations of telephone compared with in-person counseling, the involvement of only one pharmacy staff member per call, limitations of the qualitative data collection, and the possibility of undetected recognition of the mystery calls (pages 30-32, lines 671-741).**   - Generally the Methodology is **strong in design and execution**, but:   - Some **important biases are insufficiently addressed or acknowledged**   However, the author need to address the following concern;   - Interviewer (MC) effect   - MC identity significantly affects the outcomes. This is a critical limitation, not just a findings, as it indicates measurement bias but also suggest lack of full standardization as the Author did not indicate clustering adjustment any where. Therefore interviewer effect not adequately controlled   **We thank the reviewer for raising this important point. We agree that the significant association between MC identity and the AO indicates residual between-caller variability despite the extensive standardization procedures used in the study. When multiple MCs are involved, complete standardization of all caller characteristics is virtually impossible. Nevertheless, additional standardization measures could have been considered. For example, the same surname could have been used by all MCs, and voice characteristics could theoretically have been further standardized using technical voice modification. We have therefore expanded the Limitations section to acknowledge these possibilities explicitly (page 31, lines 708-713). At the same time, potential between-caller variability was minimized through standardized responses, extensive training and validation, and random assignment of CPs to MCs, and was additionally examined by including MC identity as an independent variable in the regression analysis. We therefore interpret the observed MC effect as evidence of residual between-caller variability rather than, by itself, as proof of systematic measurement bias. The statistical issue of clustering is addressed separately below.**   - Although CPs were randomly assigned to MCs and each MC handles 47 calls, this is likely to create a risk of systematic differences between callers interms of tone, confidence and communication style. Therefore there is a need for cluster effects adjustement and inter – caller variability analysis   **We thank the reviewer for raising this important point. We agree that systematic between-caller differences in characteristics such as tone, confidence, and communication style cannot be completely excluded when multiple MCs are used. In our study, potential inter-caller variability was explicitly examined by including MC identity as a categorical fixed effect in the regression analysis. Indeed, significant differences were identified for several MCs, demonstrating that between-caller variability was detected rather than ignored. In addition, CPs were randomly assigned to the MCs, and extensive standardization procedures, including standardized responses, training, validation calls, and a dedicated workshop, were used to minimize such differences.**  **Regarding clustering, no separate cluster-robust adjustment was applied. Given that only eight MCs constituted the potential clusters, conventional cluster-robust variance estimation would itself be subject to substantial small-cluster limitations. Nevertheless, we agree that residual within-MC correlation cannot be completely excluded. We have therefore added this aspect to the Limitations section (page 31, lines 715-717). We have also expanded the discussion of MC standardization by acknowledging that additional measures, such as using the same surname for all MCs or further standardizing voice characteristics, could have been considered (page 31, lines 708-713).**   - Limited mystery callers (MC) diversity   - All MCs were female, young 21- 24 years, and students in health - related field. This may introduce potential **systematic bias** and limits generalizability of the results and may influence the responses   **We thank the reviewer for this important comment and agree that the limited sociodemographic diversity of the MCs may restrict the generalizability of our findings. Due to the student research project setting, all MCs were young female university students. We have therefore added this aspect to the Limitations section and now explicitly acknowledge that it remains unclear whether callers of different ages, genders, educational backgrounds, or other sociodemographic characteristics would have received different counseling (page 31, lines 703-707).**   - Single scenario design   - The Author use only one scenario/ clinical case. This limits the scope of inference and the ability to generalize across EC situation.   **We thank the reviewer for this comment and agree that the use of a single scenario limits the generalizability of the findings to other clinical situations involving oral EC. This limitation is already acknowledged in the Limitations section, where we state that “other scenarios […] could lead to different results, even in cases of identical indication” (page 31, lines 698-700). We therefore believe that the scope of inference is appropriately reflected in the manuscript.**   - Replacement and non – response bias   - The Author declared that the “CPs were replaced if unreachable” but the Author did not report any response rate and how many CPs were replaced and Whether unreachable CPs differ systematically   **We thank the reviewer for this comment. The manuscript reports that 23 CPs were replaced and provides the specific reasons for each replacement. Ten CPs could not be reached after three call attempts, seven were permanently closed, two showed an unplanned deviation from the scenario, two were closed at the time of the call, one requested a callback outside the specified time window, and one was duplicated in the sample. A conventional response rate was not calculated because unreachable or otherwise ineligible CPs were replaced by randomly selected CPs from the same stratum until the predefined target sample was achieved. Accordingly, we now distinguish between the target sample achieved (100.0%) and the analysis rate (85.6%) rather than reporting a conventional response rate (page 18, Figure 1). We acknowledge that systematic differences between unreachable and successfully contacted CPs beyond the documented reasons for replacement could not be assessed. However, random replacement within the corresponding stratum was used to minimize the potential for selection bias. We have also explicitly acknowledged this potential source of selection bias in the Limitations section (page 30, lines 682–683).**   - Replacement strategies may introduce selection bias if replacement differ systematically as the author using additional random draws within the strat   **We thank the reviewer for raising this point. As described in the Methods section, each replacement CP was randomly drawn from the corresponding stratum using an additional random draw. Thus, the replacement procedure preserved the stratified random sampling approach and did not systematically favor particular CPs. Nevertheless, we acknowledge that some selection bias cannot be entirely excluded if CPs requiring replacement differed systematically from those ultimately included in the study (page 30, lines 682–683).**   - The author did not report of **how often the replacement occurred** and no analysis of potential bias   **We thank the reviewer for raising this point. The frequency of replacements is already reported in the Results section: 23 of the 376 CPs had to be replaced. Furthermore, as described in the Methods section, replacement CPs were randomly selected within the corresponding stratum, thereby preserving the stratified random sampling approach.**   - Also, the author did not indicate the reasons for non-contact   **We thank the reviewer for raising this point. The reasons for replacement are already reported in the Results section. Specifically, 10 CPs could not be reached after three call attempts, seven were permanently closed, two involved an unplanned deviation from the scenario, two were closed at the time of the call, one requested a callback outside the specified time window, and one was duplicated in the sample.**   - Observer and measurement bias - According to the report the second observer seems were not blinded as they knows the study aims, records data in real time and also has the final decision in disagreements which may leads to systematic bias risk and Potential **observer expectancy bias**   **We thank the reviewer for raising this important point. The second observers were not blinded to the study aims, as knowledge of the standardized scenario and assessment criteria was necessary for accurate real-time assessment of the telephone conversations. This approach also facilitated the correct classification of statements and recommendations according to the predefined assessment items. At the same time, we acknowledge that lack of blinding may have introduced a potential risk of observer expectancy bias. This risk was reduced by the use of a standardized assessment form with predominantly predefined dichotomous items, subsequent cross-checking of the completed assessment form by the MC and second observer, and additional logical consistency checks of the datasets. Nevertheless, observer expectancy bias cannot be completely excluded, and we have added this aspect to the limitations section of the manuscript (page 30, lines 673-677).**   - Lack of reliability metrics   - The Author did not comments anything regarding inter – rater reliability   **We thank the reviewer for raising this point. Regarding inter-rater reliability, each call was monitored by a second observer who documented the assessment in real time. The MC and second observer subsequently cross-checked the completed assessment form and resolved any disagreements. Because ratings were reconciled rather than recorded independently as separate datasets, a formal inter-rater reliability coefficient such as Cohen’s kappa could not be calculated. We have added this aspect to the limitations section of the manuscript (page 30, lines 671-673).**   - and also, on inter – caller consistency measures   **We thank the reviewer for raising this point. Inter-caller consistency was addressed through several procedures already described in the manuscript. All MCs used standardized responses, underwent extensive training including multiple validation calls and a dedicated workshop, and CPs were randomly assigned to the MCs. In addition, potential between-caller variability was explicitly assessed by including MC identity as an independent variable in the regression analysis. Thus, possible caller effects were not only minimized through standardization but also examined statistically.**   - Reliance on google for validation   - This approach may not be fully reliable or standardized as it could introduce information bias, therefore Author may consider to acknowledge this as a limitation   **We thank the reviewer for raising this point. We agree that Google searches used to verify the operational status of sampled CPs may not always provide up-to-date information. However, these searches were used solely for preliminary verification and were not used to assess counseling or classify study outcomes. Moreover, CPs that were unexpectedly closed or unreachable despite this verification were replaced by randomly selected CPs from the corresponding stratum. Nevertheless, we have added this aspect to the limitations (pages 30-31, lines 690-694).**   - Limited calling window   - The author restrict calls 12.00pm – 6.00pm which is justified clinically, but; it may exclude morning staff, with difference experience levels and late hours practices which may limit generalizability across working hours   **We thank the reviewer for this helpful comment. The calling window from 12:00 p.m. to 6:00 p.m. was deliberately selected to ensure the clinical consistency of the standardized scenario, as explained in the Methods section. Nevertheless, we agree that this restricts the generalizability of our findings to counseling outside this time window. We have therefore added this aspect to the limitations (page 31, lines 700-703).**  The author declared that “ if CP was unreachable the MCs made up three calls attempt at 5 minutes interval on the same day. (three – attempt rule). This may not fully reflect real patient behavior and reduce natural variability in help – seeking behavior  **We thank the reviewer for this comment. We agree that the standardized three-attempt rule may not fully reflect the variability of real-world help-seeking behavior. However, standardization of caller behavior was necessary to ensure comparability across CPs and represents an inherent feature of the simulated patient methodology. Moreover, 88.6% of CPs were reached on the first attempt, while only 9.8% and 1.6% required a second or third attempt, respectively. In addition, the number of mystery call attempts was explicitly included as a potential influencing factor in the regression analysis and was not significantly associated with the AO. Nevertheless, we have added this aspect to the limitations section (page 31, lines 695-697).**   - Insufficient details in qualitative analysis   - The author mention Inductive category formation but information regarding: coding procedure (number of coders), reliability ( e.g intercoder agreement, and software (if used) are missing leads to weakens the trustworthiness of the qualitative findings   **Thank you for this helpful comment. We have expanded the Methods section to provide further details on the qualitative analysis, including the number of coders, the coding and category development process, the resolution of disagreements, additional validation, and the use of qualitative data analysis software (page 17, lines 405-413).**   - No clear handling of clustering in statistical analysis   - The data are clustered but analysis assumes independent observation, which may leads to underestimate standard errors and Inflate statistical significance   - Clustering not addressed statistically as the data are nested ( calls within MCs) if ignored may lead to bias standard error and affect significance testing   **We thank the reviewer for raising this important point. Regarding clustering, no separate cluster-robust adjustment was applied. Given that only eight MCs constituted the potential clusters, conventional cluster-robust variance estimation would itself be subject to substantial small-cluster limitations. Nevertheless, we agree that residual within-MC correlation cannot be completely excluded. We have therefore added this aspect to the Limitations section (page 31, lines 715–717).**   - Detection bias   - The author declared some mitigation but no discussion of whether calls were suspected   **We thank the reviewer for raising this point. No formal procedure was used to assess whether pharmacy staff suspected that the calls were part of a mystery caller study. Such an assessment would have required post-call contact with the CPs, which was incompatible with the covert, no-opt-out study design. Although none of the pharmacy staff explicitly indicated suspicion during the calls, undetected recognition cannot be excluded. This issue was already addressed in the Limitations section of the manuscript. However, we have revised the wording to make the potential risk of detection bias more explicit (page 32, lines 735-741).**   - Inconsistency in sample size reporting   - The author state “ 376 CPs are sampled but only 322 CPs included in analysis and you do not explain the drop either non-response or exclusion which raises the question about selection bias and data completeness   **We thank the reviewer for pointing this out. We agree that the reason for the difference between the 376 mystery calls conducted and the 322 calls included in the final analysis should be made fully transparent. All 376 planned mystery calls were successfully conducted. However, 54 calls (14.4%) were terminated prematurely by pharmacy staff (“exits”), which prevented assessment of the primary outcome in these cases and resulted in a final analytic sample of 322 calls (85.6%). This is now explicitly described in the Results section and illustrated in the newly added flow diagram (page 18, Figure 1). In addition, we now acknowledge in the Limitations section that, because these premature terminations may not have occurred at random, some selection of the final analytic sample cannot be completely excluded (page 30, lines 682-683).**   - Slight over interpretation in conclusions   - The author recommend the use of checklist and training, these are reasonable, but; the study is observational (cross-sectional), therefore causal should be cautious - Improve neutrality and precision in interpretation   **We thank the reviewer for this comment. We agree that, given the cross-sectional design of our study, causal interpretations should be made cautiously. We have therefore explicitly added to the Limitations section that temporal and causal relationships between the studied variables cannot be established (page 30, lines 678-679).**  **In addition, we have moderated the wording of the recommendations in the Abstract (page 4, lines 71-72) and Discussion (page 27, line 565) to improve neutrality and precision. The recommendations regarding checklist use and training are intended as potential measures for improving counseling, supported by the observed findings and previous literature, rather than as causal effects demonstrated by the present study.** |
| 3 | **RESULTS** | Generally, this is a comprehensive and data-rich results section with:   - Clear reporting of sample flow and exclusions - Robust descriptive statistics - Appropriate regression analysis - Inclusion of qualitative findings   **Thank you very much for your positive assessment.**  However, the needs to clarify on the some aspects stipulated below:   - Critical inconsistency “100% call completion rate versus replacements. “ “376 mystery calls were successfully conducted (call completion rate: 100.0%)”   - The Author reported earlier that 23 CPs replaced, 54 exits, duplication and deviations. This makes 100% completion rate misleading   **We thank the reviewer for this helpful comment. We agree that the term “call completion rate” could be misunderstood, particularly in light of the prematurely terminated calls. We have therefore replaced this term with “target sample achieved,” indicating that all 376 planned mystery calls were successfully conducted after replacements. We have also clarified that 54 calls (14.4%) were classified as exits, resulting in a final analytic sample of 322 calls (85.6%). In addition, we have added a flow diagram (Figure 1) showing the target population, stratified random sample, replaced CPs, successfully conducted mystery calls, exits, and final analytic sample (page 18, Figure 1).**   - Minor inconsistency in denominators   Such as:   - 70.1% (227/324) - 71.8% (234/326) - This indicates varying missing data per item but not clearly explained   **We thank the reviewer for raising this point. The varying denominators result from premature termination of some telephone conversations (exits), meaning that not all assessment items could be completed in all mystery calls. This is already explicitly stated in the Results section: “Some interlocutors terminated the telephone conversation prematurely (exit) (14.4%, 54/376), which meant that not all items of the assessment form could be completed for all 376 mystery calls. Consequently, the sample sizes for items 1 to 21 differ.”**   - Qualitative results are too compressed. The Author summarize 97 statement to 4 categories but; there is no proportion of positive versus negative categories and also no weighting of the themes   **We thank the reviewer for this helpful comment. During the revision, we identified that the originally reported total of 97 statements was incorrect. Rechecking the coded qualitative data confirmed that the correct total is 99 statements, and this value has been corrected throughout the revised manuscript. We have also expanded the presentation of the qualitative results. Specifically, we now provide a comprehensive presentation of the statements reflecting positive and problematic counseling aspects. Of the 99 statements, 35 (35.4%) reflected positive counseling aspects, whereas 64 (64.6%) reflected problematic counseling aspects. In addition, we have added absolute and relative frequencies for each of the 11 subcategories to Table 7 to indicate their frequency distribution. The corresponding Results section has been revised accordingly (page 23, lines 494–501; page 24, Table 7).** |
|  |  |  |
| 4 | **General comments** | - Clear and relevant research question - Good sample size justification - Strong sampling and study design - Incredible use of SPM methodology - Good statistical analysis - High practical relevance   **Thank you very much for your positive assessment.** |
|  |  |  |
