# Supplementary material for: Do community pharmacies unnecessarily recommend oral emergency contraception? A nationwide mystery caller investigation in Germany
Source: PLoS One. 2026 Oct 23;21(10):e0361027. doi: 10.1371/journal.pone.0361027 (PMC13693899; doi:10.1371/journal.pone.0361027)
Supplement: Peer review history

# Peer review: Decision Letter 0

**DOI:** 10.1371/journal.pone.0361027.r001

**Authors:** Arupendra Mozumdar

21 Jul 2026

PONE-D-26-20770

Do community pharmacies unnecessarily recommend oral emergency contraception? A nationwide mystery caller investigation in Germany

PLOS One?

Dear Dr. Langer,

Thank you for submitting your manuscript to PLOS ONE. After careful consideration, we feel that it has merit but does not fully meet PLOS ONE’s publication criteria as it currently stands. Therefore, we invite you to submit a revised version of the manuscript that addresses the points raised during the review process.

Please submit your revised manuscript by Sep 04 2026 11:59PM. If you will need more time than this to complete your revisions, please reply to this message or contact the journal office at plosone@plos.org. When you're ready to submit your revision, log on to https://www.editorialmanager.com/pone/ and select the 'Submissions Needing Revision' folder to locate your manuscript file.

- A letter that responds to each point raised by the academic editor and reviewer(s). You should upload this letter as a separate file labeled 'Response to Reviewers'.
- A marked-up copy of your manuscript that highlights changes made to the original version. You should upload this as a separate file labeled 'Revised Manuscript with Track Changes'.
- An unmarked version of your revised paper without tracked changes. You should upload this as a separate file labeled 'Manuscript'.

If you would like to make changes to your financial disclosure, please include your updated statement in your cover letter. Guidelines for resubmitting your figure files are available below the reviewer comments at the end of this letter.

If applicable, we recommend that you deposit your laboratory protocols in protocols.io to enhance the reproducibility of your results. Protocols.io assigns your protocol its own identifier (DOI) so that it can be cited independently in the future. For instructions see: https://journals.plos.org/plosone/s/submission-guidelines#loc-laboratory-protocols. Additionally, PLOS ONE offers an option for publishing peer-reviewed Lab Protocol articles, which describe protocols hosted on protocols.io. Read more information on sharing protocols at https://plos.org/protocols?utm_medium=editorial-email&utm_source=authorletters&utm_campaign=protocols.

As the corresponding author, your ORCID iD is verified in the submission system and will appear in the published article. PLOS supports the use of ORCID, and we encourage all coauthors to register for an ORCID iD and use it as well. Please encourage your coauthors to verify their ORCID iD within the submission system before final acceptance, as unverified ORCID iDs will not appear in the published article. *Only*  the individual author can complete the verification step; PLOS staff *cannot*  verify ORCID iDs on behalf of authors.

We look forward to receiving your revised manuscript.

Kind regards,

Arupendra Mozumdar, Ph.D., M.Sc.

Academic Editor

PLOS One

Journal Requirements:

When submitting your revision, we need you to address these additional requirements.

1. Please ensure that your manuscript meets PLOS ONE's style requirements, including those for file naming. The PLOS ONE style templates can be found at

https://journals.plos.org/plosone/s/file?id=wjVg/PLOSOne_formatting_sample_main_body.pdf and

https://journals.plos.org/plosone/s/file?id=ba62/PLOSOne_formatting_sample_title_authors_affiliations.pdf.

2.Please provide additional details regarding participant consent. In the ethics statement in the Methods and online submission information, please ensure that you have specified (1) whether consent was informed and (2) what type you obtained (for instance, written or verbal, and if verbal, how it was documented and witnessed). If your study included minors, state whether you obtained consent from parents or guardians. If the need for consent was waived by the ethics committee, please include this information.

If you are reporting a retrospective study of medical records or archived samples, please ensure that you have discussed whether all data were fully anonymized before you accessed them and/or whether the IRB or ethics committee waived the requirement for informed consent. If patients provided informed written consent to have data from their medical records used in research, please include this information.

3. We note that your Data Availability Statement is currently as follows: [All relevant data are within the manuscript. ]

Please confirm at this time whether or not your submission contains all raw data required to replicate the results of your study. Authors must share the “minimal data set” for their submission. PLOS defines the minimal data set to consist of the data required to replicate all study findings reported in the article, as well as related metadata and methods (https://journals.plos.org/plosone/s/data-availability#loc-minimal-data-set-definition).

For example, authors should submit the following data:

- The values behind the means, standard deviations and other measures reported;

- The values used to build graphs;

- The points extracted from images for analysis.

Authors do not need to submit their entire data set if only a portion of the data was used in the reported study.

If your submission does not contain these data, please either upload them as Supporting Information files or deposit them to a stable, public repository and provide us with the relevant URLs, DOIs, or accession numbers. For a list of recommended repositories, please see https://journals.plos.org/plosone/s/recommended-repositories.

If there are ethical or legal restrictions on sharing a de-identified data set, please explain them in detail (e.g., data contain potentially sensitive information, data are owned by a third-party organization, etc.) and who has imposed them (e.g., an ethics committee). Please also provide contact information for a data access committee, ethics committee, or other institutional body to which data requests may be sent. If data are owned by a third party, please indicate how others may request data access.

4. Please be informed that funding information should not appear in the Acknowledgments section or other areas of your manuscript. We will only publish funding information present in the Funding Statement section of the online submission form. Please remove any funding-related text from the manuscript.

5. If the reviewer comments include a recommendation to cite specific previously published works, please review and evaluate these publications to determine whether they are relevant and should be cited. There is no requirement to cite these works unless the editor has indicated otherwise.

6. Please review your reference list to ensure that it is complete and correct. If you have cited papers that have been retracted, please include the rationale for doing so in the manuscript text, or remove these references and replace them with relevant current references. Any changes to the reference list should be mentioned in the rebuttal letter that accompanies your revised manuscript. If you need to cite a retracted article, indicate the article’s retracted status in the References list and also include a citation and full reference for the retraction notice.

Additional Editor Comments (if provided):

Requesting authors to address comments and suggestions from the reviewer and submit a revised version of the manuscript.

[Note: HTML markup is below. Please do not edit.]

Reviewers' comments:

Reviewer's Responses to Questions

**Comments to the Author**

1. Is the manuscript technically sound, and do the data support the conclusions?

Reviewer #1: Yes

Reviewer #2: Yes

Reviewer #3: Yes

Reviewer #4: Yes

**********

2. Has the statistical analysis been performed appropriately and rigorously?

Reviewer #1: Yes

Reviewer #2: Yes

Reviewer #3: Yes

Reviewer #4: Yes

**********

3. Have the authors made all data underlying the findings in their manuscript fully available?

The PLOS Data policy

Reviewer #1: Yes

Reviewer #2: Yes

Reviewer #3: Yes

Reviewer #4: Yes

**********

4. Is the manuscript presented in an intelligible fashion and written in standard English?

Reviewer #1: Yes

Reviewer #2: Yes

Reviewer #3: Yes

Reviewer #4: Yes

**********

Reviewer #1: This manuscript addresses an important public health and pharmacy practice question: whether German community pharmacies recommend oral emergency contraception when it is not indicated.

Find below comments to improve the manuscript

1. Sample size calculation used P=0.5 (maximum variance), which is appropriate for an unknown proportion. However, with an observed AO of 44%, the margin of error around this estimate should be reported.

2. The study reports 376 completed mystery calls, but the primary outcome analysis is based on 322 calls, as 54 were terminated prematurely. This is a major analytic decision and requires clearer justification.

The authors should explain whether excluding these 54 “exit” calls could bias the estimate of the appropriate outcome. In real-world care, premature refusal or inability to counsel is itself a clinically relevant counseling outcome, especially for emergency contraception. Excluding these cases may overestimate the proportion of appropriate recommendations.

The manuscript reports that 376 mystery calls were conducted successfully and that the call completion rate was 100.0%. Yet 54 calls ended prematurely, and 23 pharmacies had to be replaced. This could be misunderstood. Please define “completion rate” precisely. A flow diagram would greatly improve transparency: sampled pharmacies, excluded/replaced pharmacies, attempted calls, reached pharmacies, premature exits, and the final analytic sample.

3. Similarly, oral EC recommendation, physician referral, and in-person visit recommendation should be clearly distinguished between “not asked/not reached because call ended” and true “No.” A sensitivity analysis treating exits as inappropriate outcomes would be useful.

4. The ethical reporting requires more detail. The study used covert mystery calls without opt-out, although pharmacies were notified in general terms. This may be ethically acceptable, but the authors should provide more detail on safeguards.

5. The manuscript reports 97 statements and presents categories and anchor examples, but the qualitative analysis is underdescribed. It is unclear who coded the data, whether more than one coder was involved, how disagreements were resolved, whether the categories were developed from transcripts/field notes, and whether any effort was made to assess coding consistency.

Because the qualitative data were not systematically collected and covered only noteworthy events, the authors should avoid language that implies prevalence or comprehensive qualitative coverage. The table is useful, but the analysis should be explicitly labeled as exploratory and illustrative.

6. The scenario hinges on a single missed combined oral contraceptive within the 12-hour window, with sexual intercourse the prior evening. The authors cite BAK guidance that oral EC is not required when the pill is taken less than 12 hours late. However, the scenario could be misunderstood by pharmacy staff if timing is not elicited precisely.

The authors should clarify whether the caller had not yet taken the missed pill at the time of the call, and whether the time of the call always ensured the 12-hour window had not elapsed. Since calls occurred between 12:00 and 18:00 and the missed pill was due at 07:00, calls near 18:00 occurred very close to the 12-hour threshold. The manuscript says calls after 18:00 were avoided because the 12-hour window was nearly over, but at 18:00, the elapsed time is already 11 hours. This should be stated more explicitly, including how calls at exactly or near 18:00 were handled.

7. Use one term consistently: “oral emergency contraception,” “oral EC,” or “morning-after pill.” The manuscript moves between these terms; this is understandable, but should be standardized.

8. The finding that specific mystery callers (MC 2-5) had significantly higher odds of achieving the appropriate outcome (AORs ranging from 6.31 to 24.19) is striking, but the explanation remains speculative. The authors discuss voice characteristics and last names, but no objective voice measurements were collected.

Reviewer #2: I would appreciate clarification regarding the following points:

Conceptual concern: The study appears to interpret the failure to follow a checklist as indicative of inappropriate practice. However, as stated in Line 133, the checklist items are neither mandatory nor explicitly recommended by the relevant guideline. Therefore, the rationale for considering non-adherence to this checklist as evidence of inappropriate recommendations requires further justification. Please clarify the basis for this assumption and provide supporting evidence from the literature or professional standards.

Page 7, Lines 170–173: The statement “The first MC study … but did not address unnecessary recommendations” is not sufficiently clear. Please clarify what is meant by “unnecessary recommendations” and provide examples to help readers better understand this concept.

Page 8, Line 221: The sample size obtained using the Raosoft calculator represents the minimum required sample size. In survey based studies, it is common practice to inflate the calculated sample size (e.g., by 10–20%) to account for anticipated non-response or incomplete responses. As indicated on Page 17 (Line 398), non-response appears to have affected the sample size available for Items 1–29. Please justify the sampling strategy adopted and discuss the potential implications of the reduced sample size on the study findings.

Page 15, Line 342: The statement “These checks demonstrated a very high level of data quality” requires further support. Please provide the evidence, criteria, or results that substantiate this conclusion.

Page 17, Table 4: The variable “Purchasing Power Characteristic” requires further explanation. Please clarify how this variable was calculated or categorized, what it represents, and its relevance to the study objective of investigating unnecessary recommendations of oral emergency contraception.

Reviewer #3: General Evaluation

This is a well-designed national mystery-caller study using a recognised and robust methodology. The study addresses an important aspect of emergency contraception (EC) counselling in community pharmacies and provides useful information about guideline adherence and information gathering by pharmacy staff.

The manuscript could be strengthened by explaining more clearly why this research question is clinically important. The discussion could also place the findings in a broader reproductive health context and provide a more balanced overview of the role of community pharmacists in contraceptive care. In addition to avoiding unnecessary EC use, pharmacy consultations may provide opportunities to improve contraceptive adherence, prevent unintended pregnancy, and discuss long-term contraceptive options.

See full review attached

Reviewer #4: Generally, The study is very relevant with clear and relevant research question, virtuous sample size justification, strong sampling and study design, incredible use of SPM methodology, good statistical analysis and high practical relevance . however, the Author needs to address the issues raised clearly as stipulated on the attached reviewer comments file

**********

what does this mean?). If published, this will include your full peer review and any attached files.

If you choose “no”, your identity will remain anonymous but your review may still be made public.

**Do you want your identity to be public for this peer review?**  For information about this choice, including consent withdrawal, please see our Privacy Policy

Reviewer #1: No

Reviewer #2: No

Reviewer #3: No

Reviewer #4: No

**********

[NOTE: If reviewer comments were submitted as an attachment file, they will be attached to this email and accessible via the submission site. Please log into your account, locate the manuscript record, and check for the action link "View Attachments". If this link does not appear, there are no attachment files.]

To ensure your figures meet our technical requirements, please review our figure guidelines: https://journals.plos.org/plosone/s/figures

You may also use PLOS’s free figure tool, NAAS, to help you prepare publication quality figures: https://journals.plos.org/plosone/s/figures#loc-tools-for-figure-preparation.

NAAS will assess whether your figures meet our technical requirements by comparing each figure against our figure specifications.

Attachment Submitted filename: Reviewer Comments JUNE-2026.docx

Attachment Submitted filename: Reviewer feeedback final JK.docx

Attachment Submitted filename: PONE-D-26-20770 REVISED MAY 2026.pdf

# Author comment: Author response to Decision Letter 1

**DOI:** 10.1371/journal.pone.0361027.r002

15 Sep 2026

Please see the attached Word file for our detailed point-by-point responses to the editor and reviewers.

Attachment Submitted filename: PONE-D-26-20770 Editor Reviewer Comments Response Letter.docx

# Editor report: Decision Letter 1

**DOI:** 10.1371/journal.pone.0361027.r003

**Authors:** Arupendra Mozumdar

6 Oct 2026

Do community pharmacies unnecessarily recommend oral emergency contraception? A nationwide mystery caller investigation in Germany

PONE-D-26-20770R1

Dear Dr. Langer,

We’re pleased to inform you that your manuscript has been judged scientifically suitable for publication and will be formally accepted for publication once it meets all outstanding technical requirements.

Within one week, you’ll receive an e-mail detailing the required amendments. When these have been addressed, you’ll receive a formal acceptance letter and your manuscript will be scheduled for publication.

An invoice will be generated when your article is formally accepted. Please note, if your institution has a publishing partnership with PLOS and your article meets the relevant criteria, all or part of your publication costs will be covered. Please make sure your user information is up-to-date by logging into Editorial Manager at Editorial Manager® and clicking the ‘Update My Information' link at the top of the page. For questions related to billing, please contact billing support.

If your institution or institutions have a press office, please notify them about your upcoming paper to help maximize its impact. If they’ll be preparing press materials, please inform our press team as soon as possible -- no later than 48 hours after receiving the formal acceptance. Your manuscript will remain under strict press embargo until 2 pm Eastern Time on the date of publication. For more information, please contact onepress@plos.org.

Kind regards,

Arupendra Mozumdar, Ph.D., M.Sc.

Academic Editor

PLOS One

Additional Editor Comments (optional):

It is my immense pleasure to inform you that I have recommended your manuscript to be accepted for publication in PLOS ONE. We have completed two rounds of the peer review process, and I believe this process has significantly enhanced the quality of your manuscript. Your findings bring to light the critical need for better counseling for clients seeking credible information on contraceptive use in private, unbiased settings. This study will contribute significantly to the existing literature on family planning and contraceptive use.

Reviewers' comments:

# Editor report: Acceptance letter

**DOI:** 10.1371/journal.pone.0361027.r004

**Authors:** Arupendra Mozumdar

PONE-D-26-20770R1

PLOS One

Dear Dr. Langer,

I'm pleased to inform you that your manuscript has been deemed suitable for publication in PLOS One. Congratulations! Your manuscript is now being handed over to our production team.

At this stage, our production department will prepare your paper for publication. This includes ensuring the following:

* All references, tables, and figures are properly cited

* All relevant supporting information is included in the manuscript submission,

* There are no issues that prevent the paper from being properly typeset

You will receive further instructions from the production team, including instructions on how to review your proof when it is ready. Please keep in mind that we are working through a large volume of accepted articles, so please give us a few days to review your paper and let you know the next and final steps.

Lastly, if your institution or institutions have a press office, please let them know about your upcoming paper now to help maximize its impact. If they'll be preparing press materials, please inform our press team within the next 48 hours. Your manuscript will remain under strict press embargo until 2 pm Eastern Time on the date of publication. For more information, please contact onepress@plos.org.

You will receive an invoice from PLOS for your publication fee after your manuscript has reached the completed accept phase. If you receive an email requesting payment before acceptance or for any other service, this may be a phishing scheme. Learn how to identify phishing emails and protect your accounts at https://explore.plos.org/phishing.

If we can help with anything else, please email us at customercare@plos.org.

Thank you for submitting your work to PLOS One and supporting open access.

Kind regards,

PLOS One Editorial Office Staff

on behalf of

Dr. Arupendra Mozumdar

Academic Editor

PLOS One
